# Supplementary figures and images for: Genetic inactivation of zinc transporter SLC39A5 improves liver function and hyperglycemia in obesogenic settings
Source: eLife. 2024 Dec 13;12:RP90419. doi: 10.7554/eLife.90419 (PMC11648992; doi:10.7554/eLife.90419)

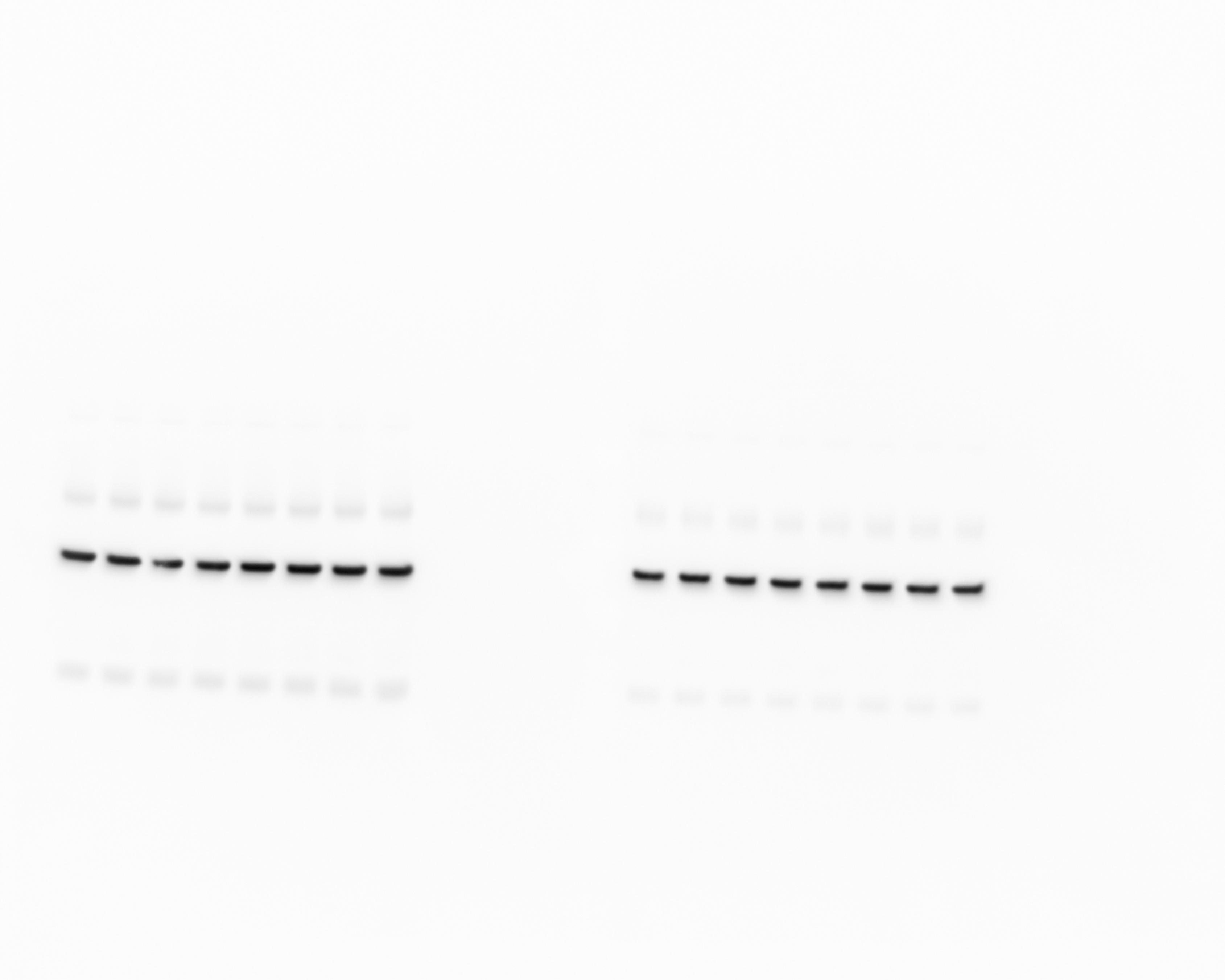

Supplement: Figure 2—figure supplement 1—source data 1. [file elife-90419-fig2-figsupp1-data1.zip › Figure 2-figure supplement 1_raw images/Fig2s1C Bactin.jpg]

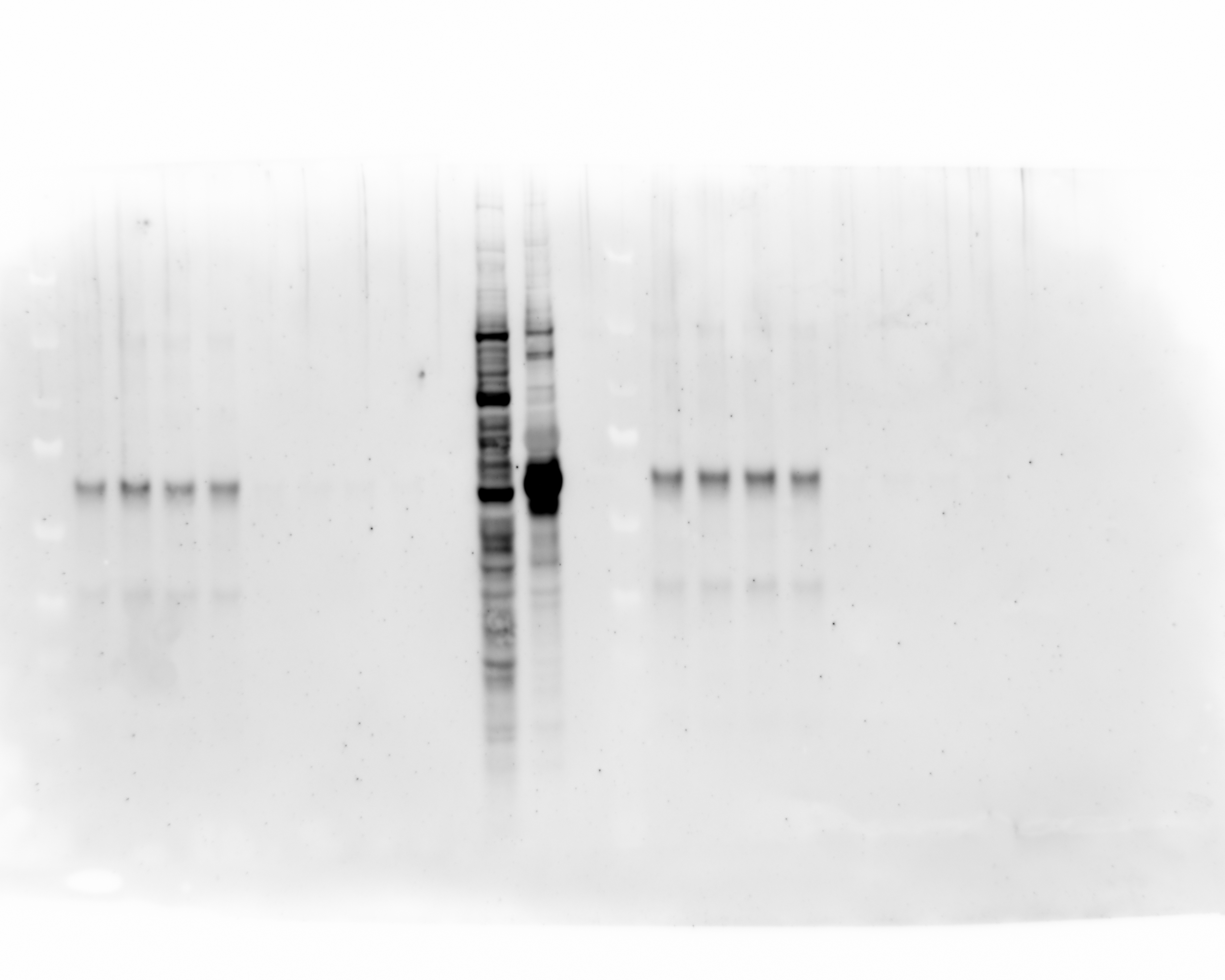

Supplement: Figure 2—figure supplement 1—source data 1. [file elife-90419-fig2-figsupp1-data1.zip › Figure 2-figure supplement 1_raw images/Fig2s1C SLC39A5.jpg]

Figure 2-figure supplement 1

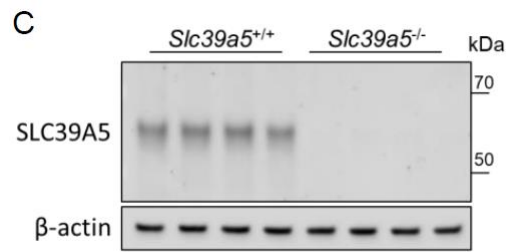

SLC39A5

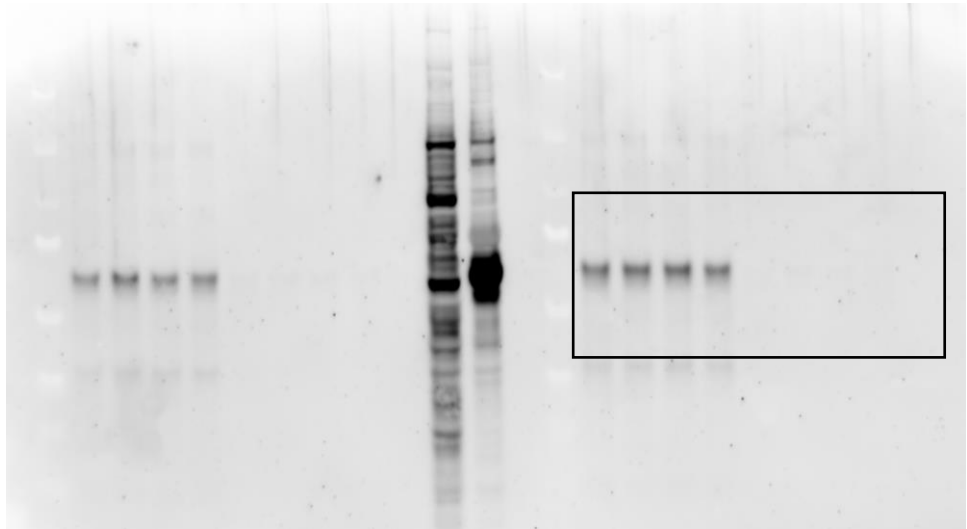

Bactin

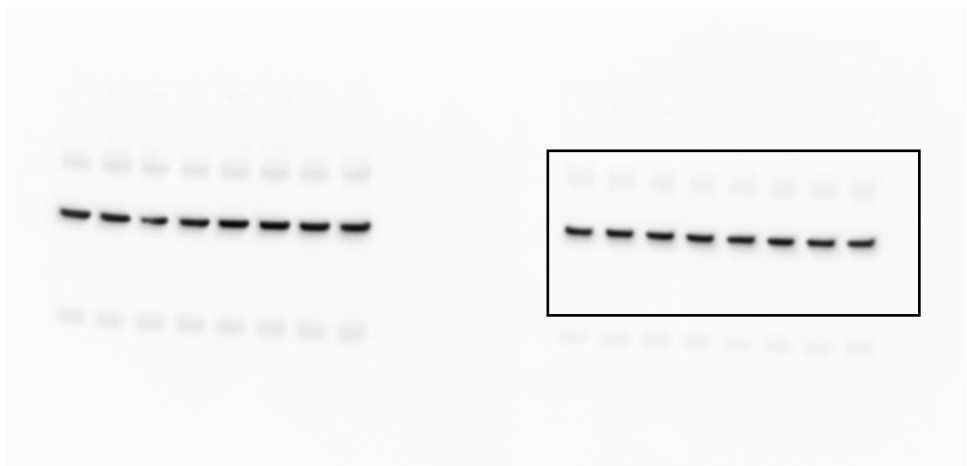

Supplement: Figure 2—figure supplement 1—source data 2. [file elife-90419-fig2-figsupp1-data2.zip › Figure 2-figure supplement 1_uncropped_labelled_images/Fig2s1_uncropped_labelled_images.pdf]

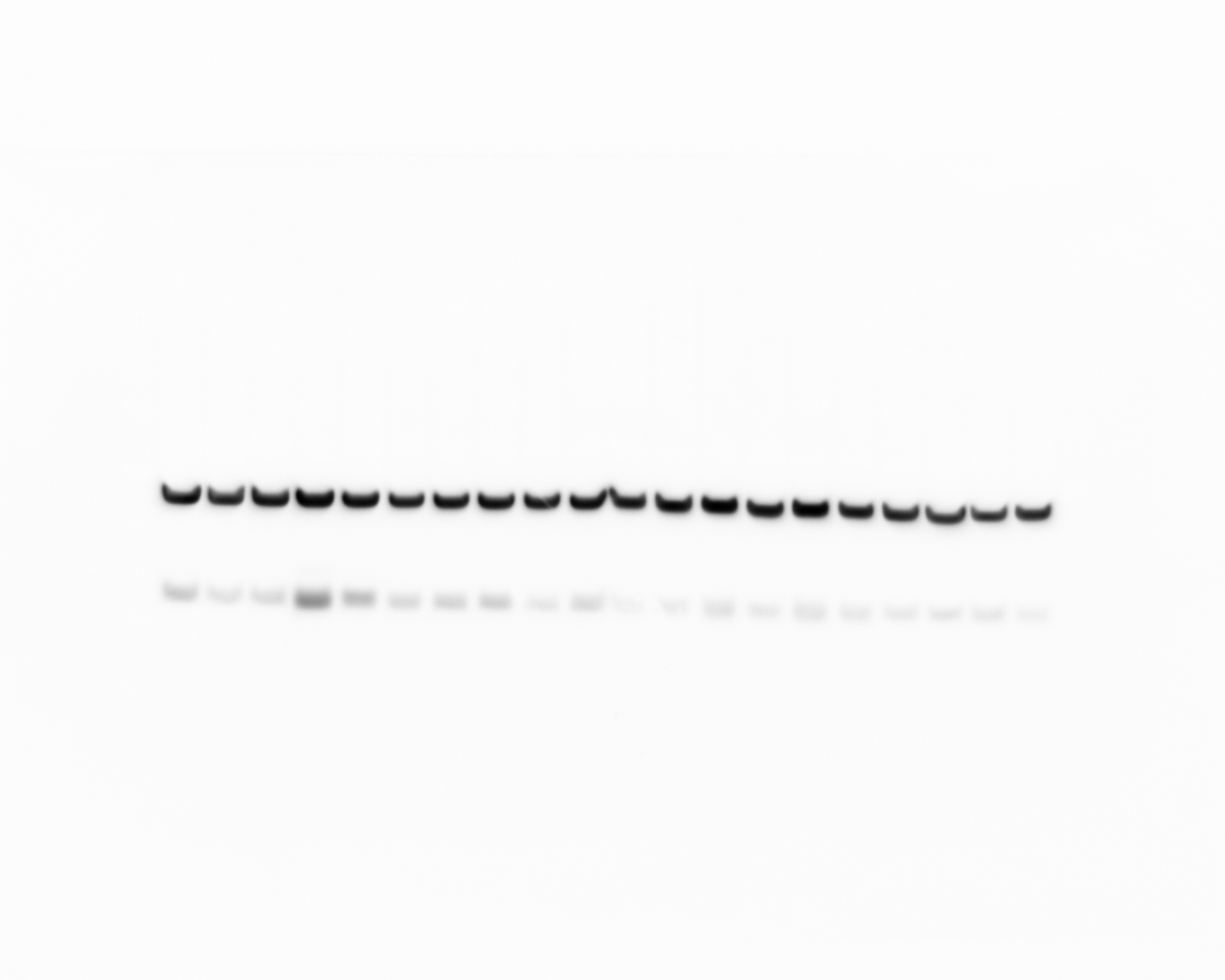

Supplement: Figure 3—figure supplement 1—source data 1. [file elife-90419-fig3-figsupp1-data1.zip › Figure 3-figure supplement 1_raw images/Fig3s1 Bactin.jpg]

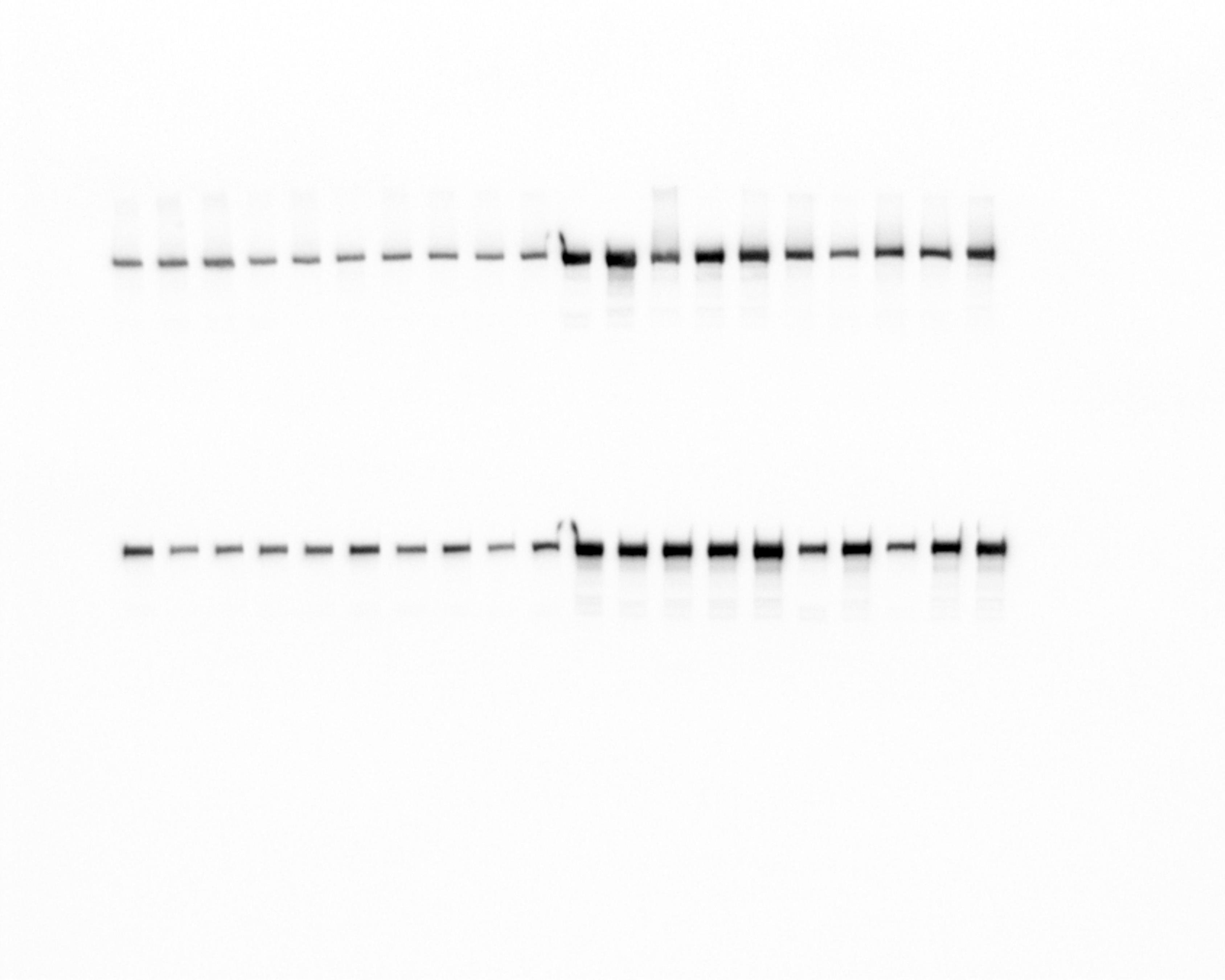

Supplement: Figure 3—figure supplement 1—source data 1. [file elife-90419-fig3-figsupp1-data1.zip › Figure 3-figure supplement 1_raw images/Fig3s1 FASN.jpg]

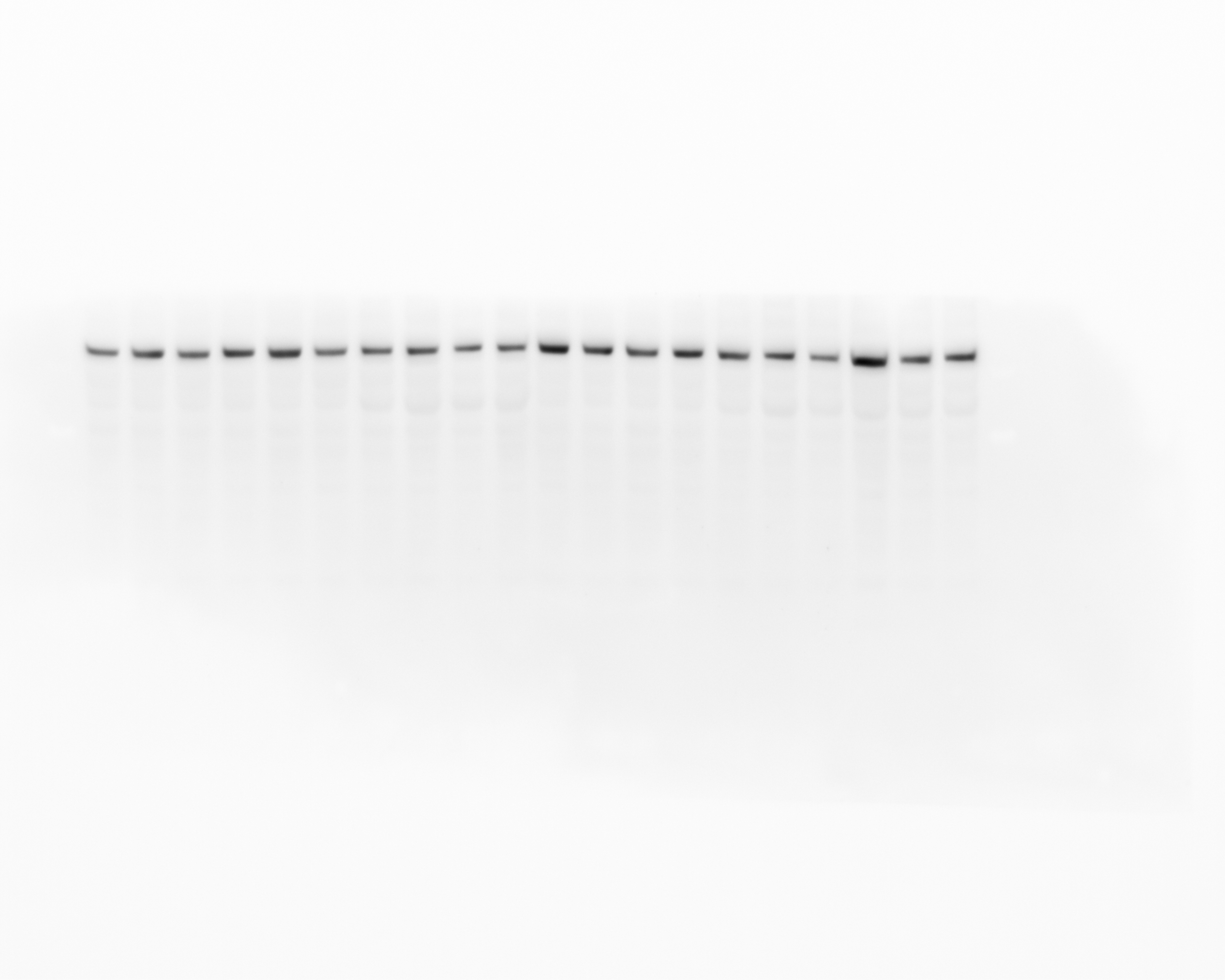

Supplement: Figure 3—figure supplement 1—source data 1. [file elife-90419-fig3-figsupp1-data1.zip › Figure 3-figure supplement 1_raw images/Fig3s1 G6PC.jpg]

Figure 3-figure supplement 1

C

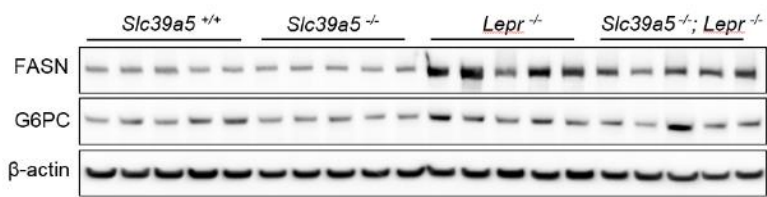

FASN

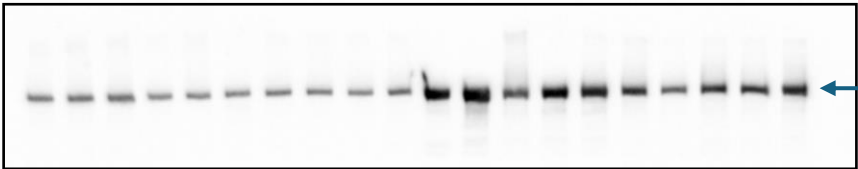

Female

G6PC

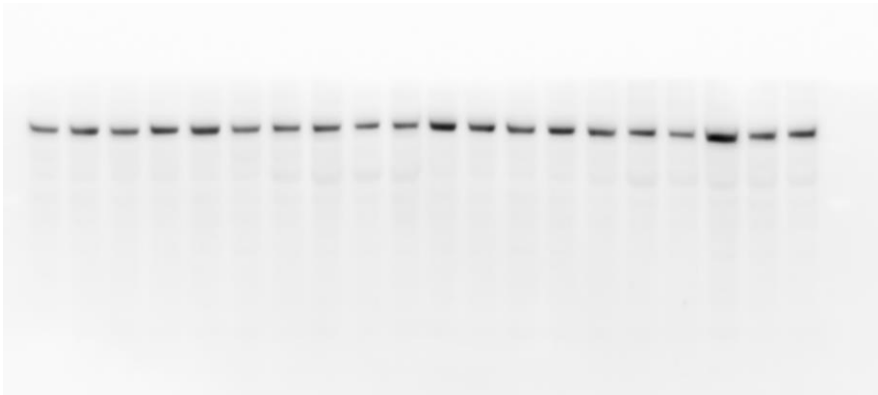

Bactin

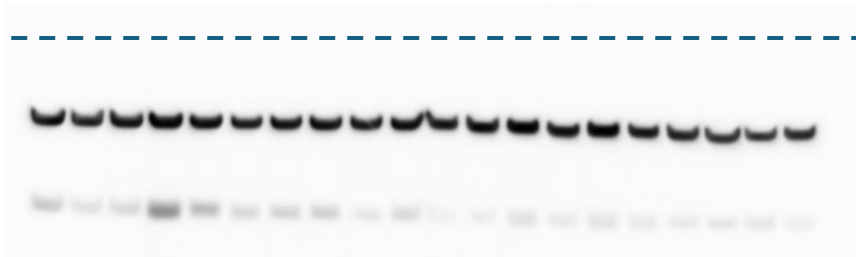

Supplement: Figure 3—figure supplement 1—source data 2. [file elife-90419-fig3-figsupp1-data2.zip › Figure 3-figure supplement 1_uncropped_labelled_images/Fig3s1_uncropped_labelled_images.pdf]

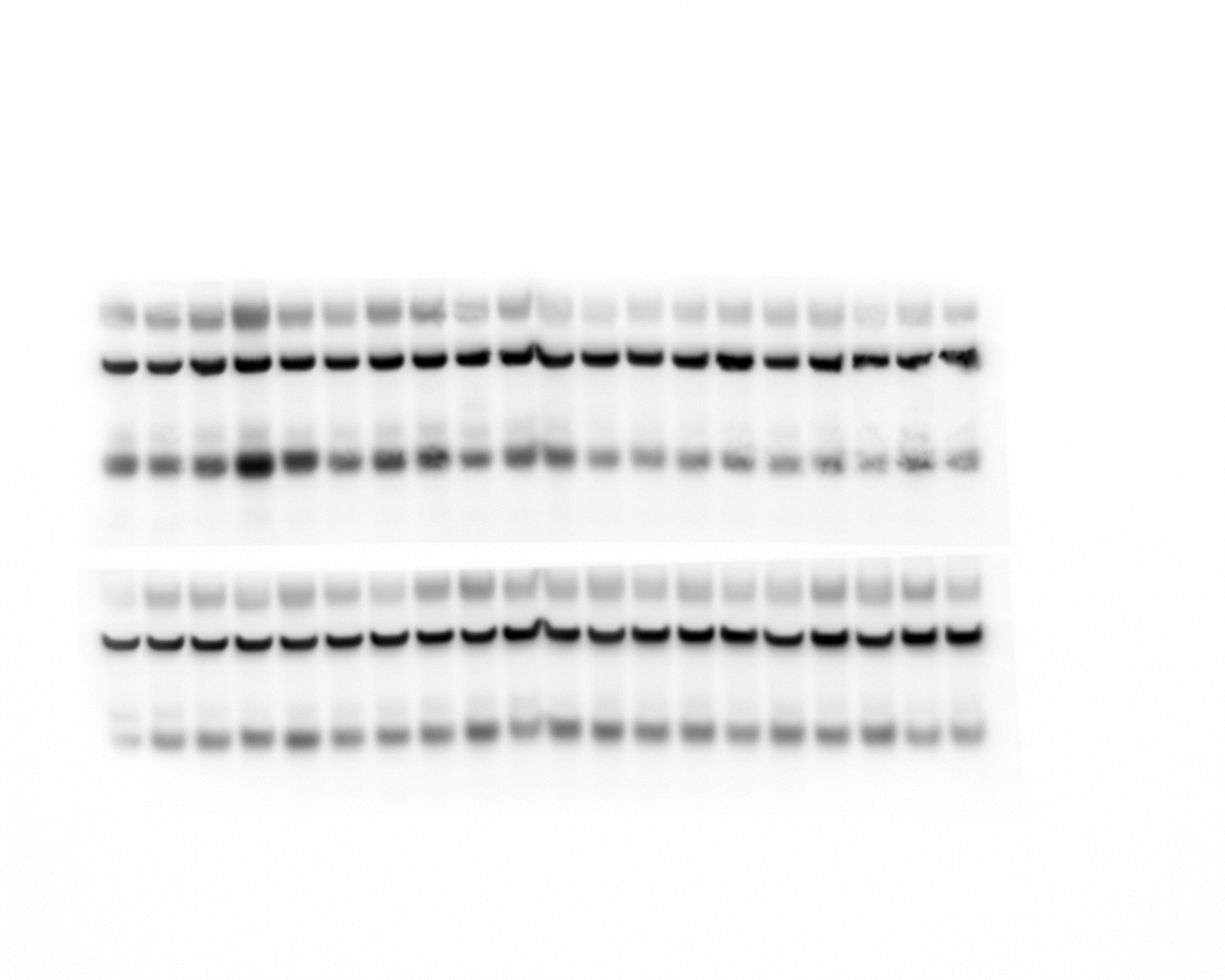

Supplement: Figure 3—figure supplement 2—source data 1. [file elife-90419-fig3-figsupp2-data1.zip › Figure 3-figure supplement 2_raw images/Fig3s2 Bactin.jpg]

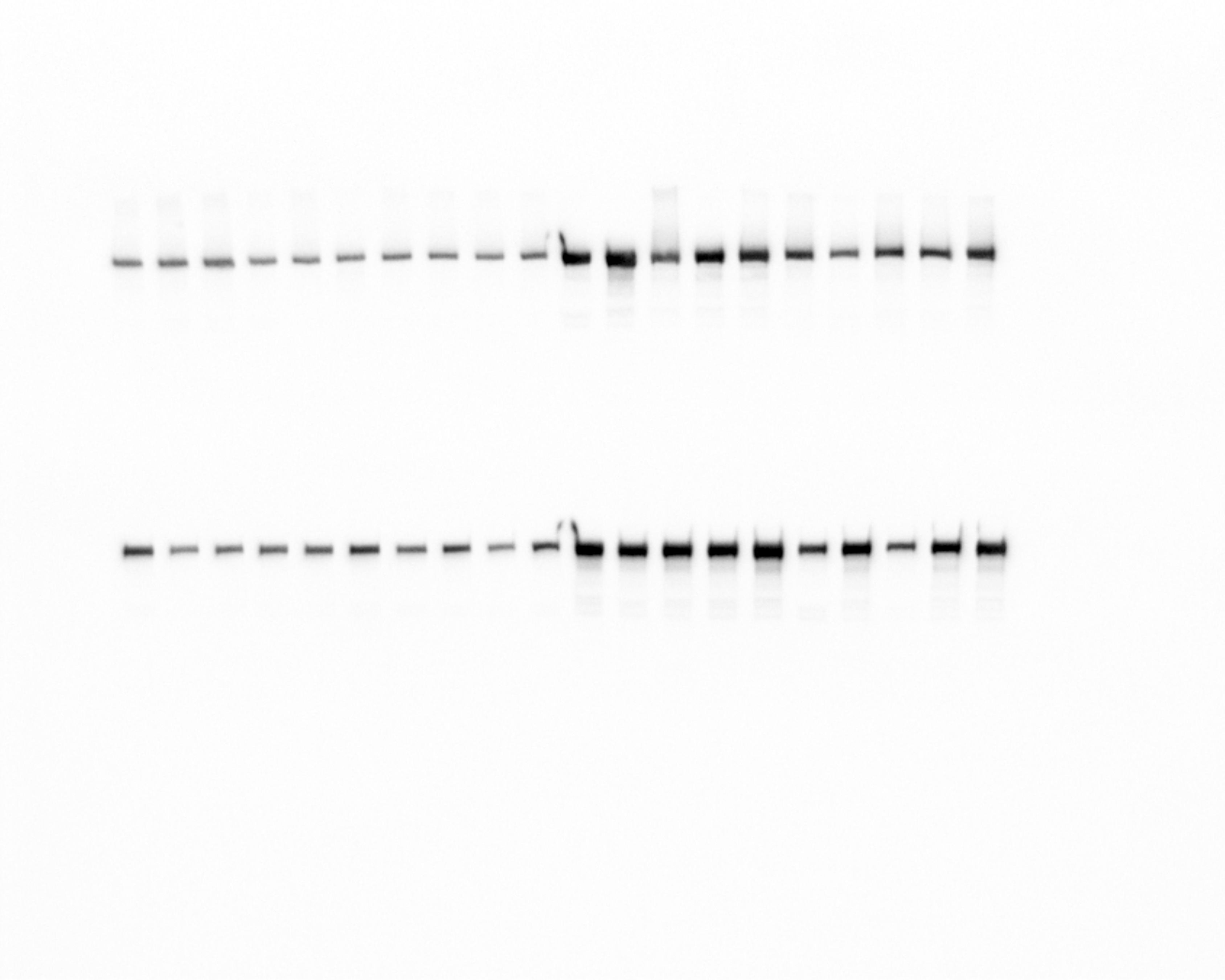

Supplement: Figure 3—figure supplement 2—source data 1. [file elife-90419-fig3-figsupp2-data1.zip › Figure 3-figure supplement 2_raw images/Fig3s2 FASN.jpg]

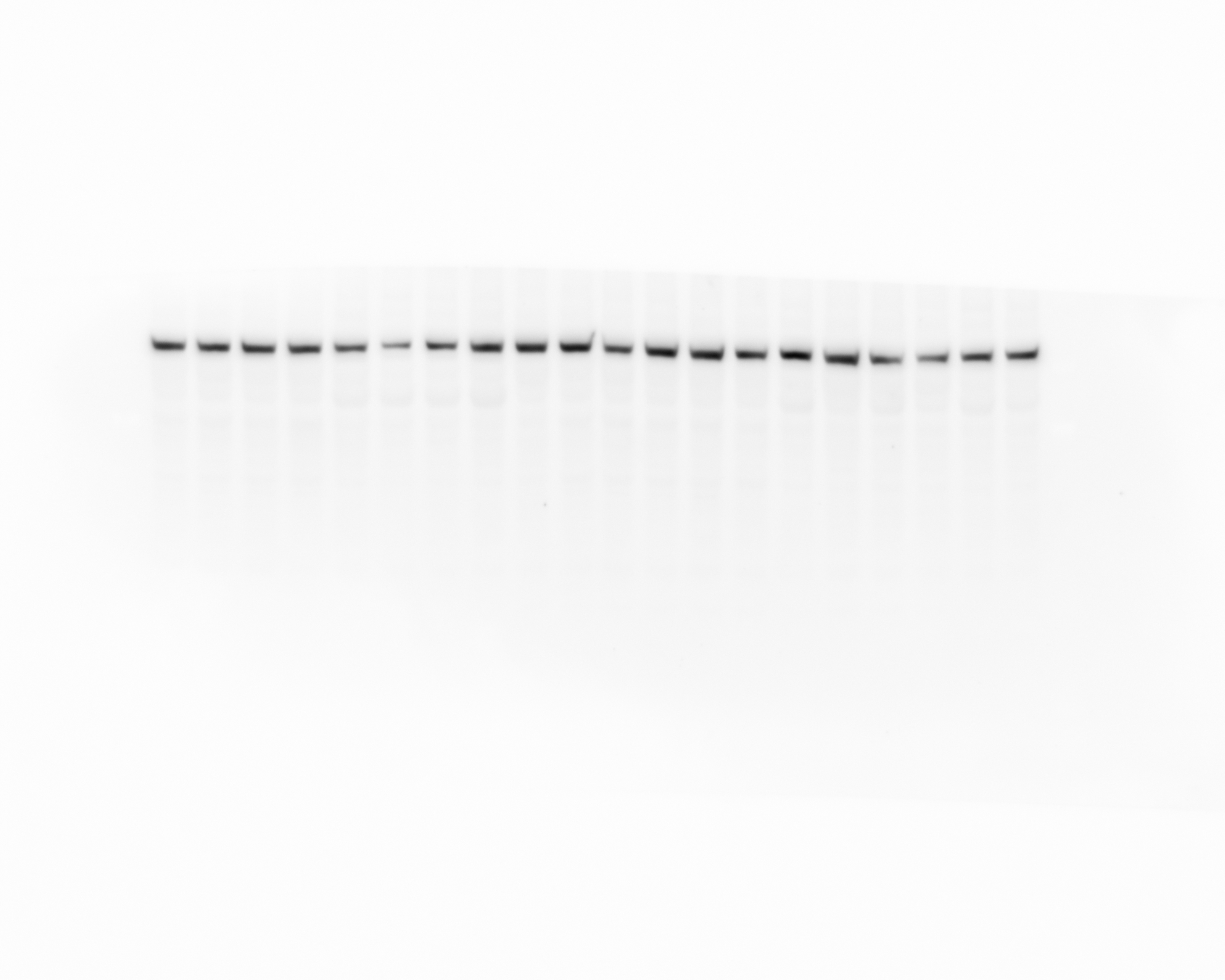

Supplement: Figure 3—figure supplement 2—source data 1. [file elife-90419-fig3-figsupp2-data1.zip › Figure 3-figure supplement 2_raw images/Fig3s2 G6PC.jpg]

Figure 3-figure supplement 2

C

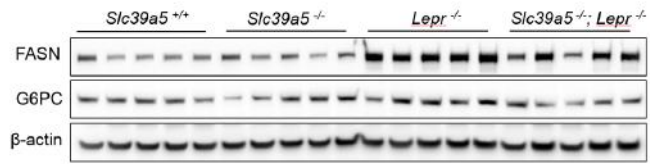

FASN

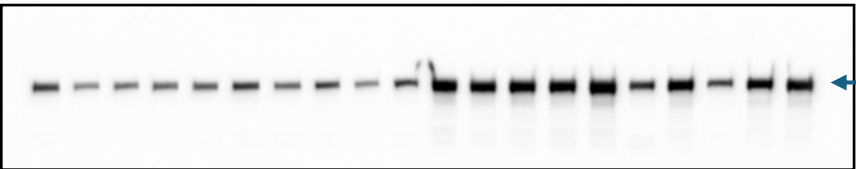

Male

G6PC

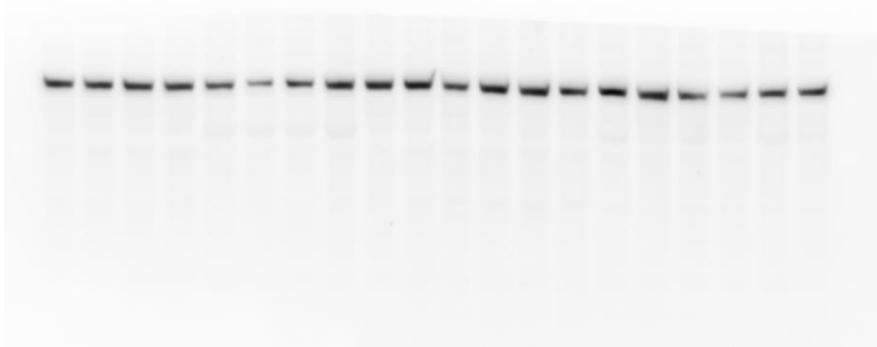

Bactin

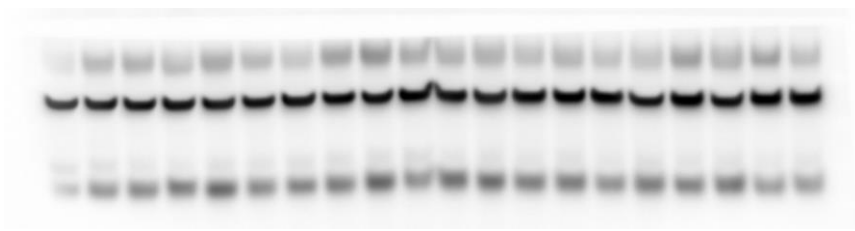

Supplement: Figure 3—figure supplement 2—source data 2. [file elife-90419-fig3-figsupp2-data2.zip › Figure 3-figure supplement 2_uncropped_labelled_images/Fig3s2_uncropped_labelled_images.pdf]

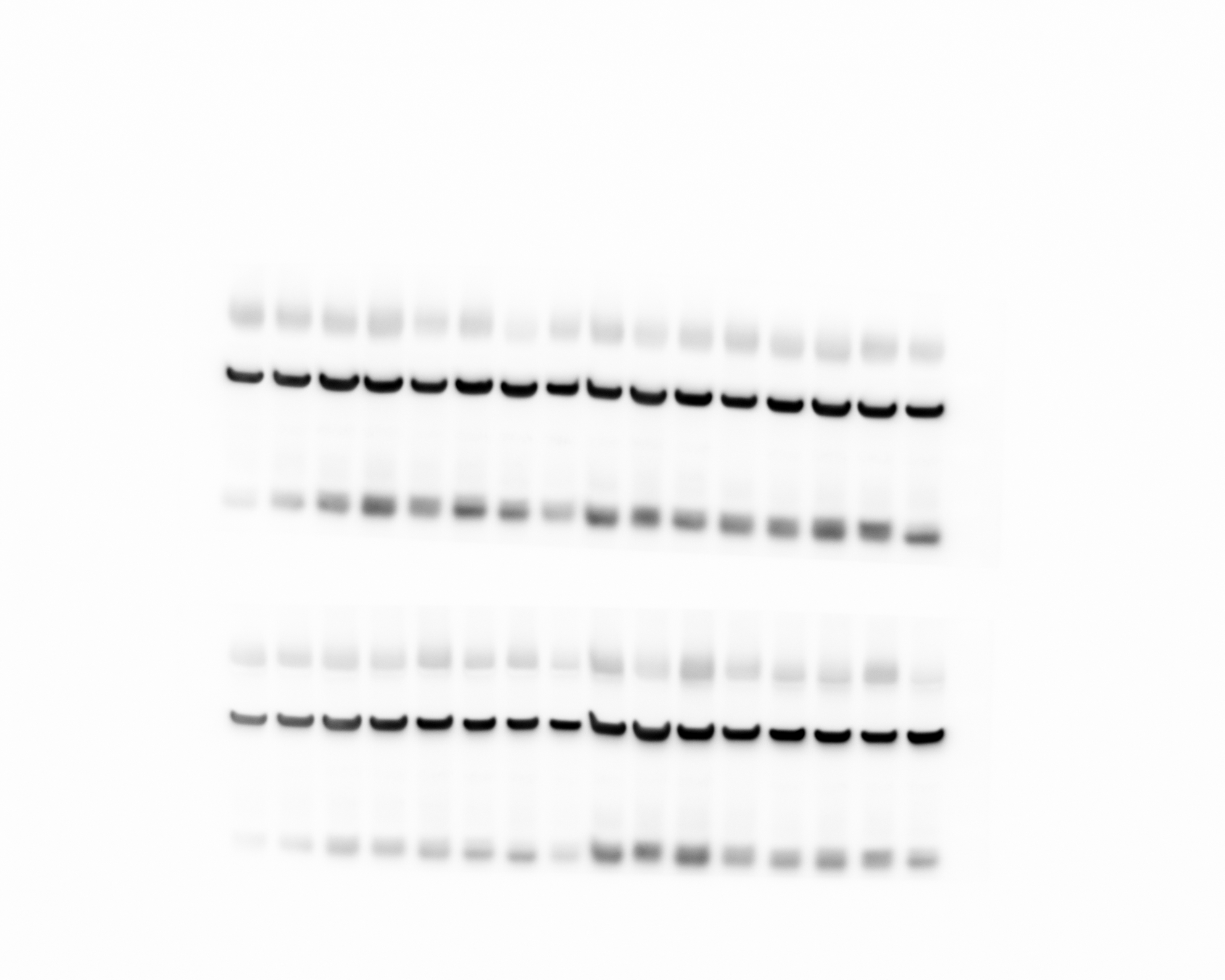

Supplement: Figure 3—figure supplement 3—source data 1. [file elife-90419-fig3-figsupp3-data1.zip › Figure 3-figure supplement 3_raw images/Fig3s3 Bactin.jpg]

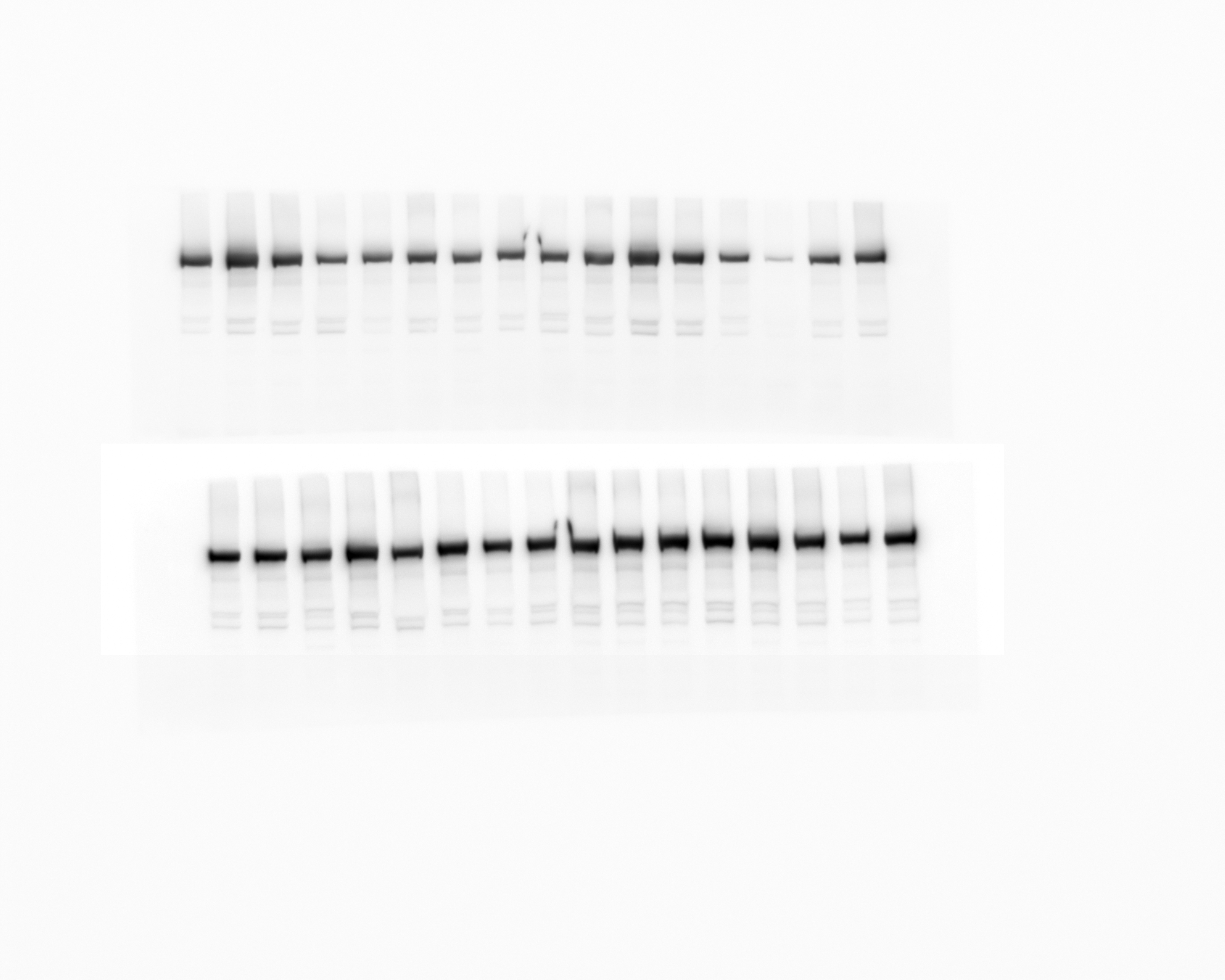

Supplement: Figure 3—figure supplement 3—source data 1. [file elife-90419-fig3-figsupp3-data1.zip › Figure 3-figure supplement 3_raw images/Fig3s3 FASN.jpg]

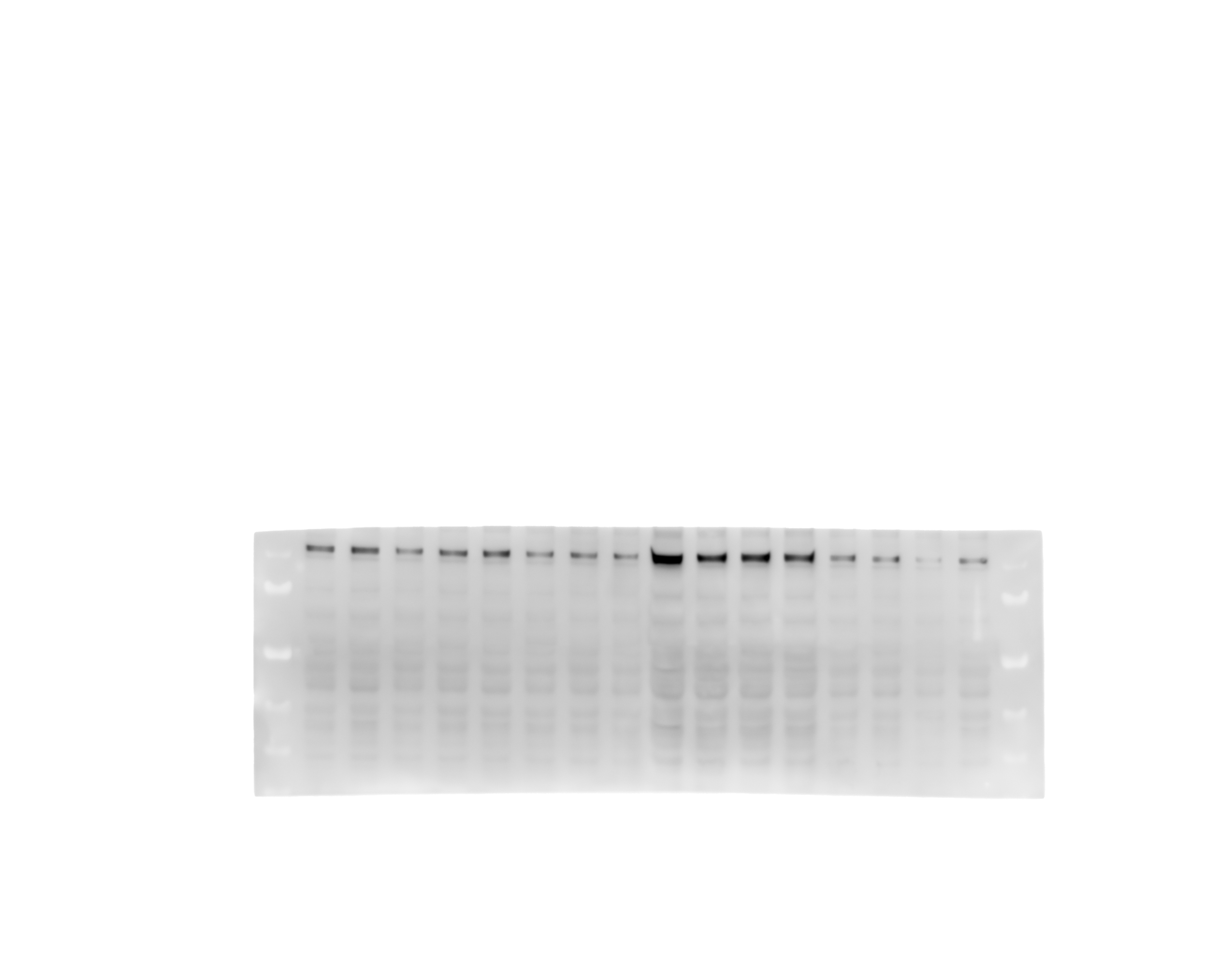

Supplement: Figure 3—figure supplement 3—source data 1. [file elife-90419-fig3-figsupp3-data1.zip › Figure 3-figure supplement 3_raw images/Fig3s3 G6PC.jpg]

Figure 3-figure supplement 3

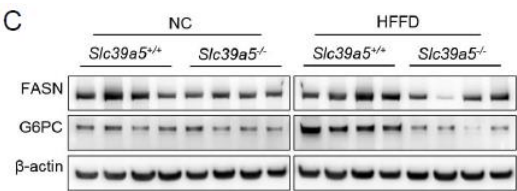

FASN

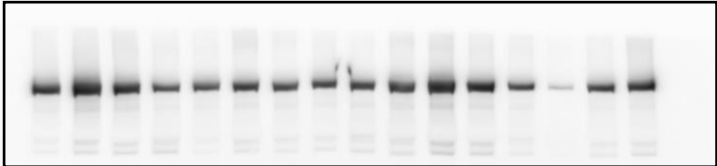

G6PC

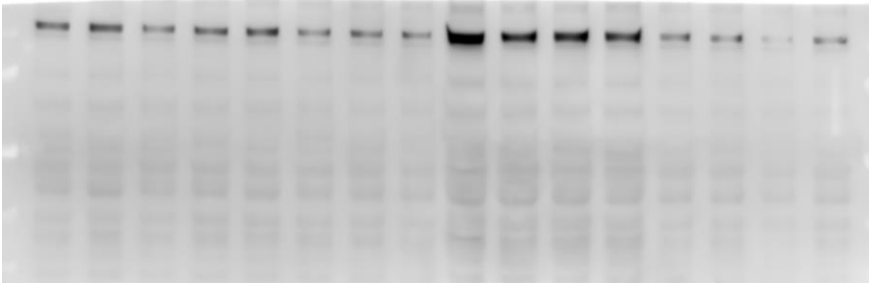

Bactin

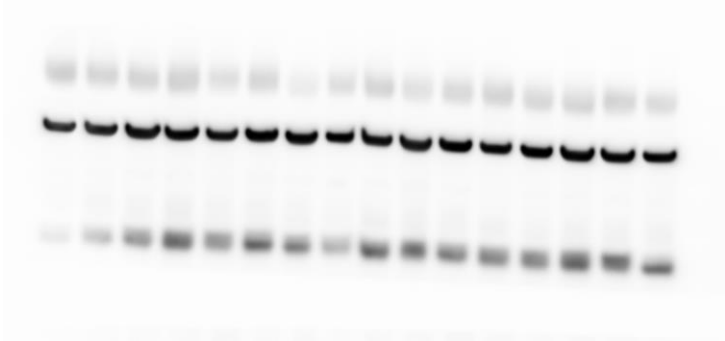

Supplement: Figure 3—figure supplement 3—source data 2. [file elife-90419-fig3-figsupp3-data2.zip › Figure 3-figure supplement 3_uncropped_labelled_images/SFig6_uncropped_labelled_images.pdf]

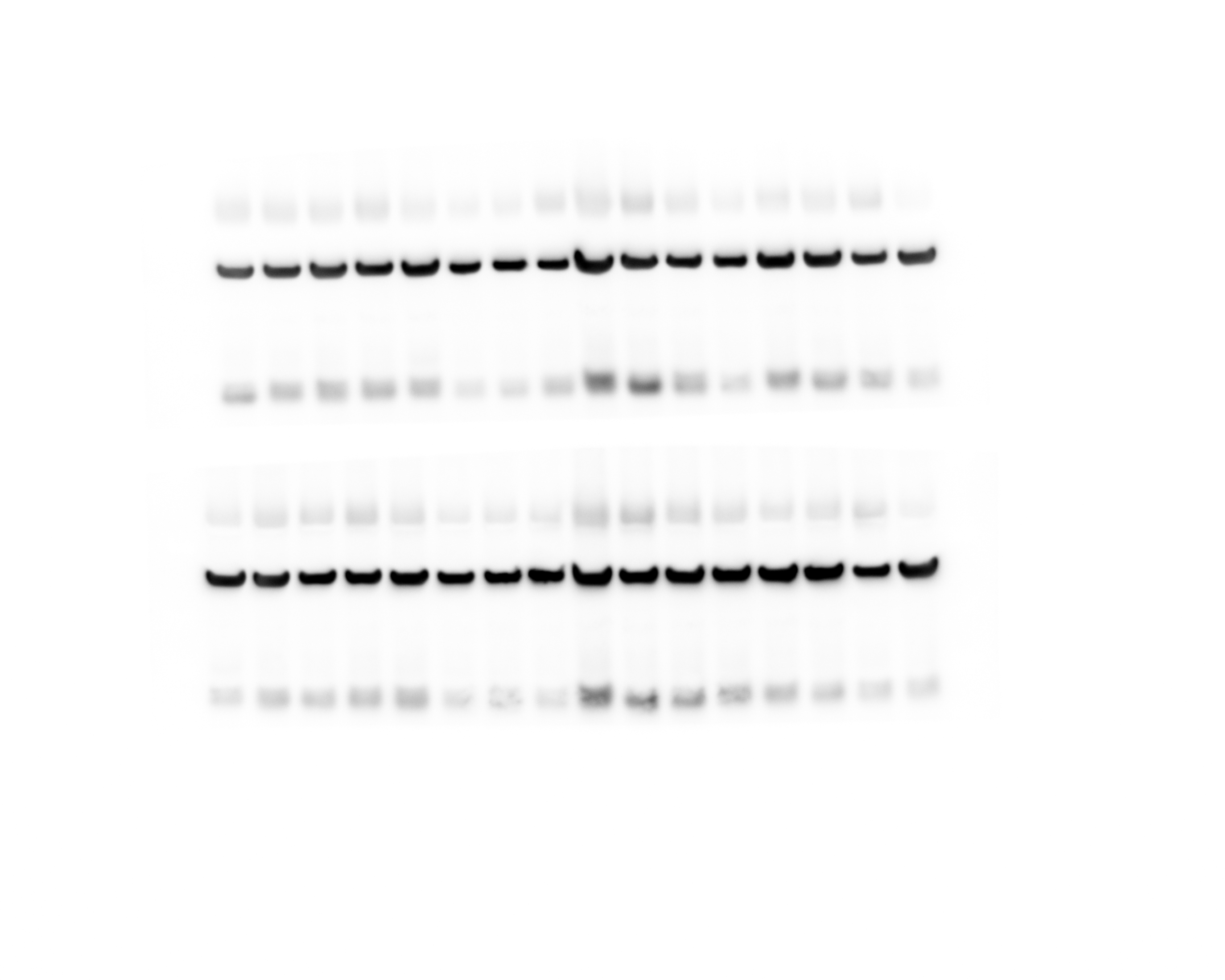

Supplement: Figure 3—figure supplement 4—source data 1. [file elife-90419-fig3-figsupp4-data1.zip › Figure 3-figure supplement 4_raw images/Fig3s4 Bactin.jpg]

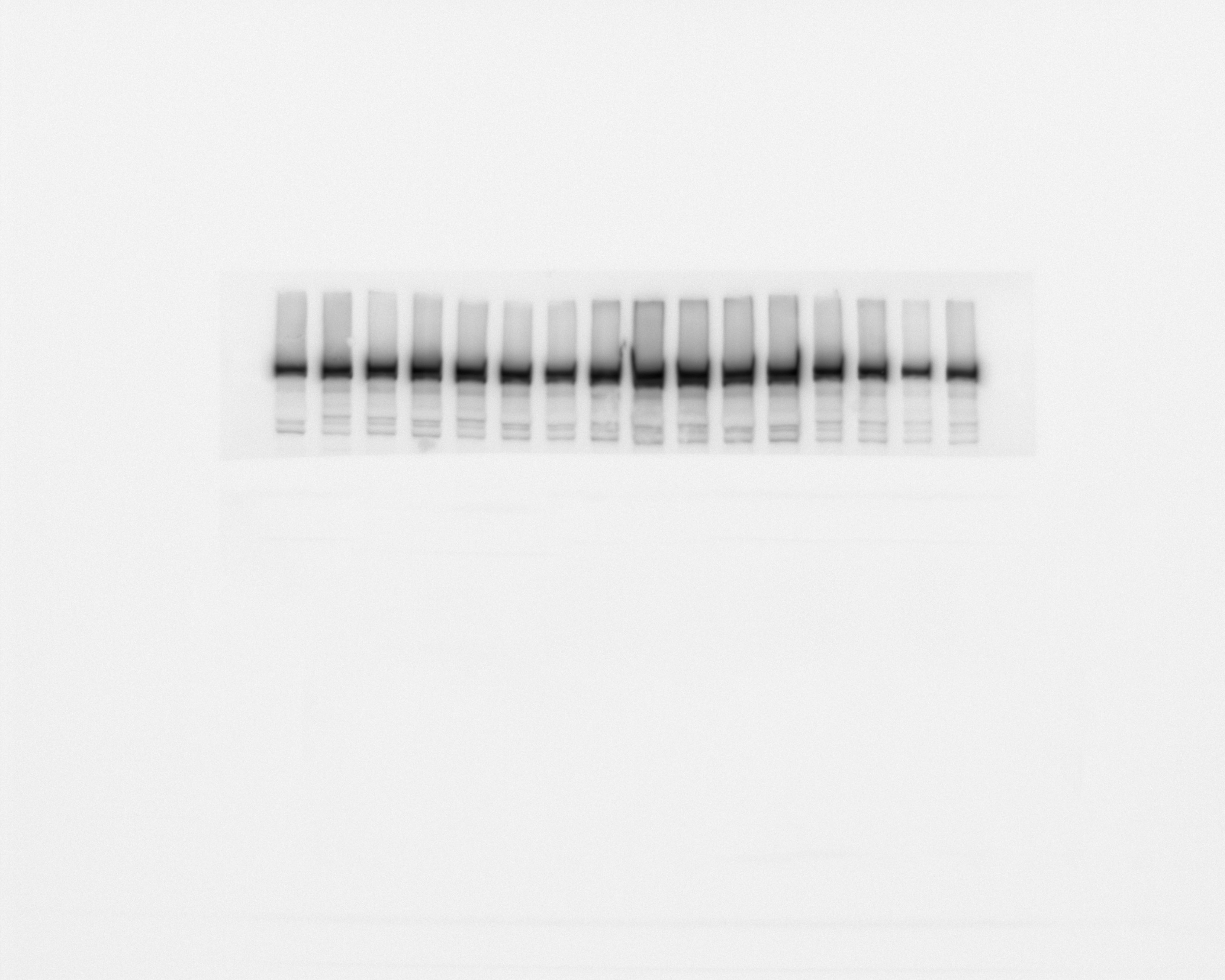

Supplement: Figure 3—figure supplement 4—source data 1. [file elife-90419-fig3-figsupp4-data1.zip › Figure 3-figure supplement 4_raw images/Fig3s4 FASN.jpg]

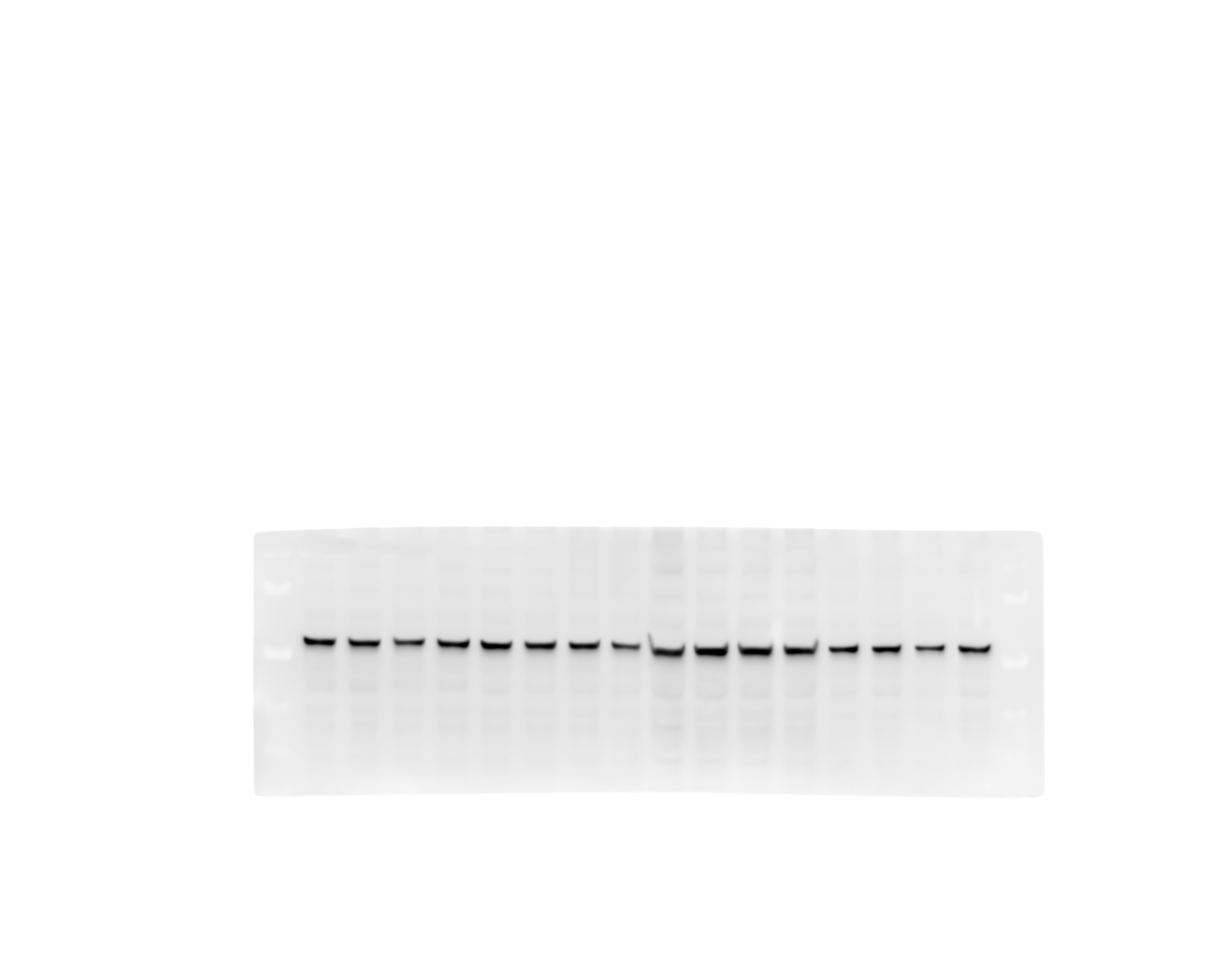

Supplement: Figure 3—figure supplement 4—source data 1. [file elife-90419-fig3-figsupp4-data1.zip › Figure 3-figure supplement 4_raw images/Fig3s4 G6PC.jpg]

Figure 3-figure supplement 4

C

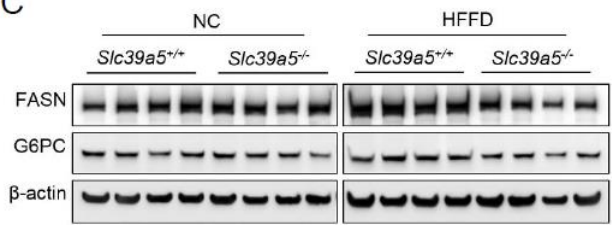

FASN

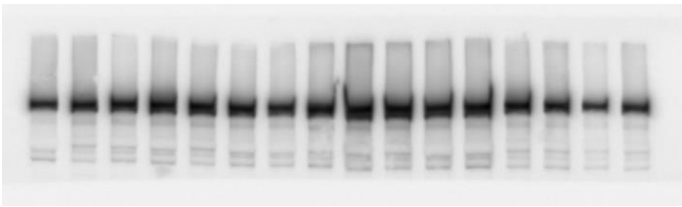

G6PC

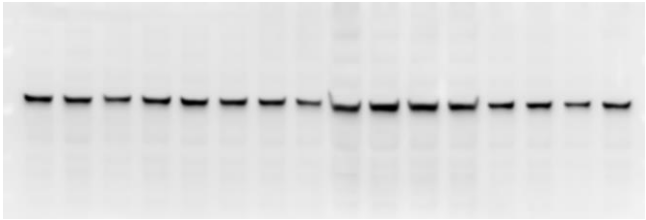

Bactin

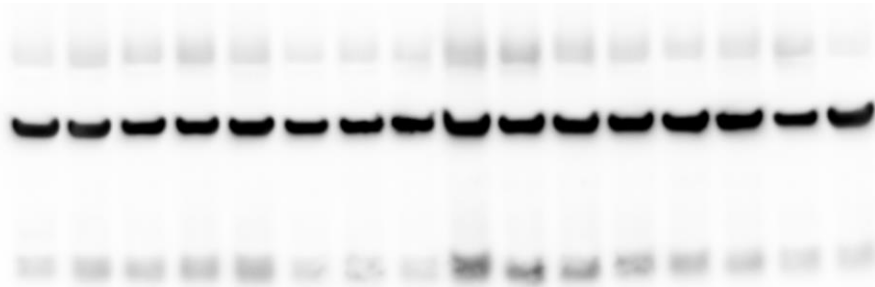

Supplement: Figure 3—figure supplement 4—source data 2. [file elife-90419-fig3-figsupp4-data2.zip › Figure 3-figure supplement 4_uncropped_labelled_images/Fig3s4_uncropped_labelled_images.pdf]

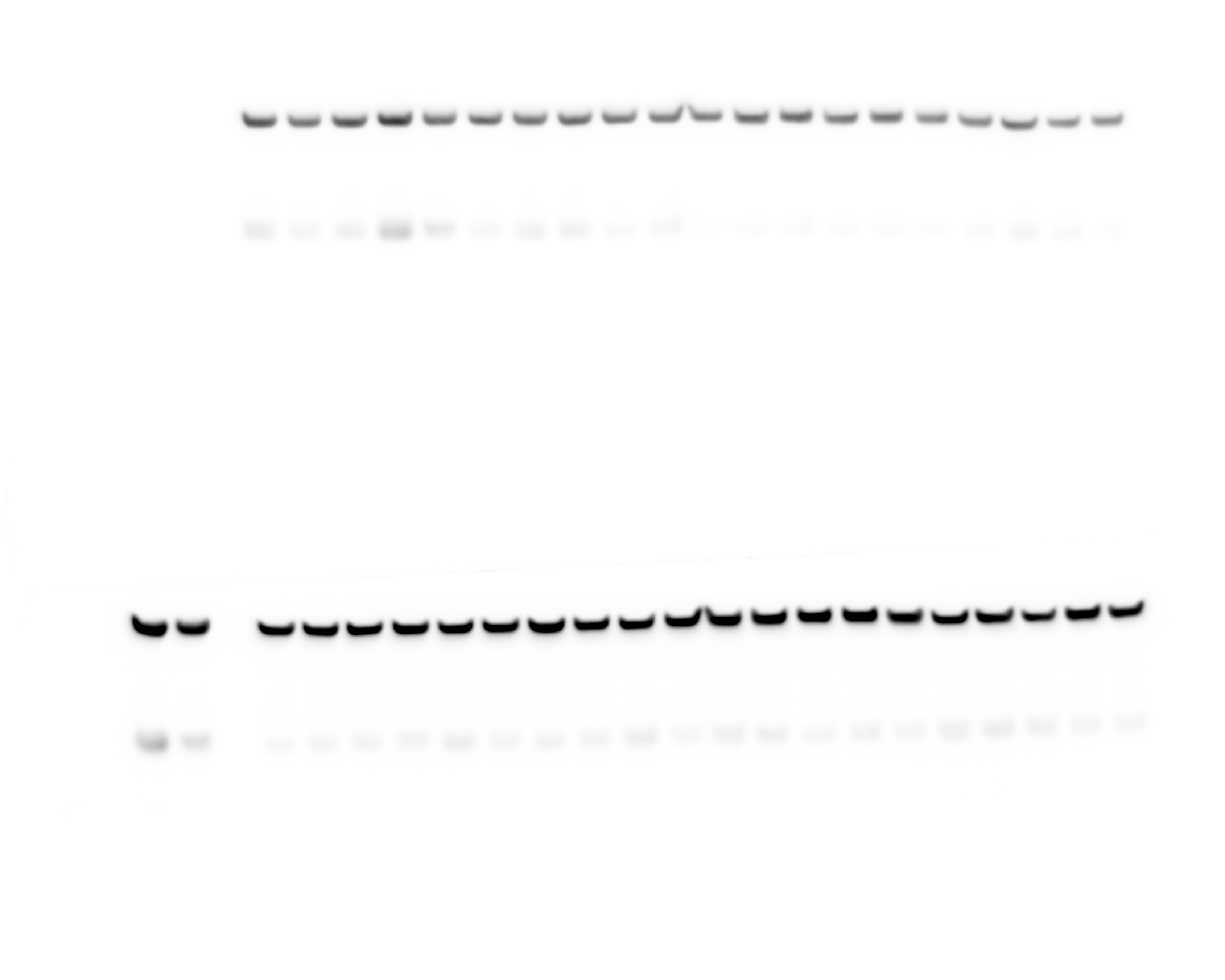

Supplement: Figure 5—source data 1. [file elife-90419-fig5-data1.zip › Fig5_raw images/Fig5A dKO F M Bactin.jpg]

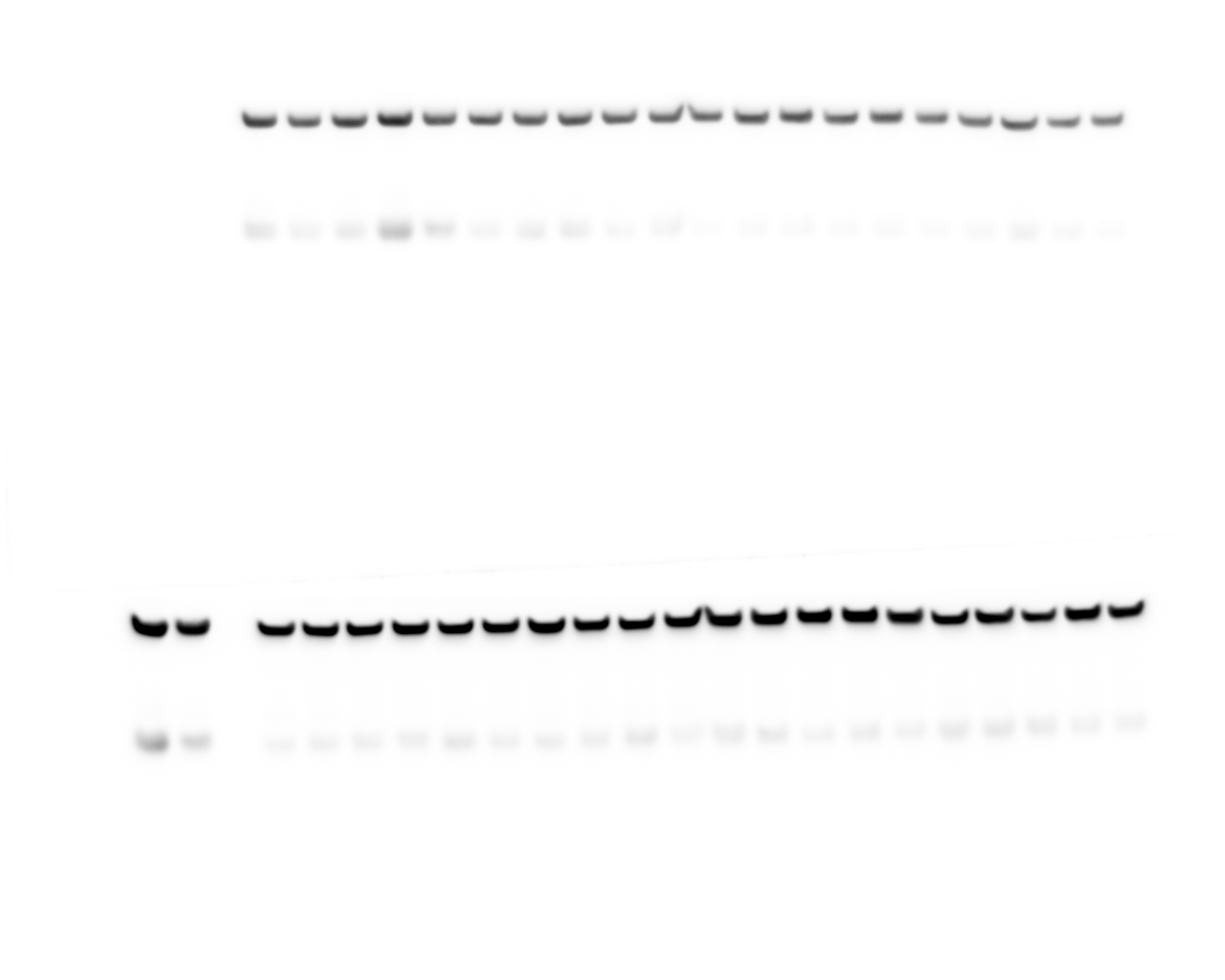

Supplement: Figure 5—source data 1. [file elife-90419-fig5-data1.zip › Fig5_raw images/Fig5A dKO F M Bactin_1.jpg]

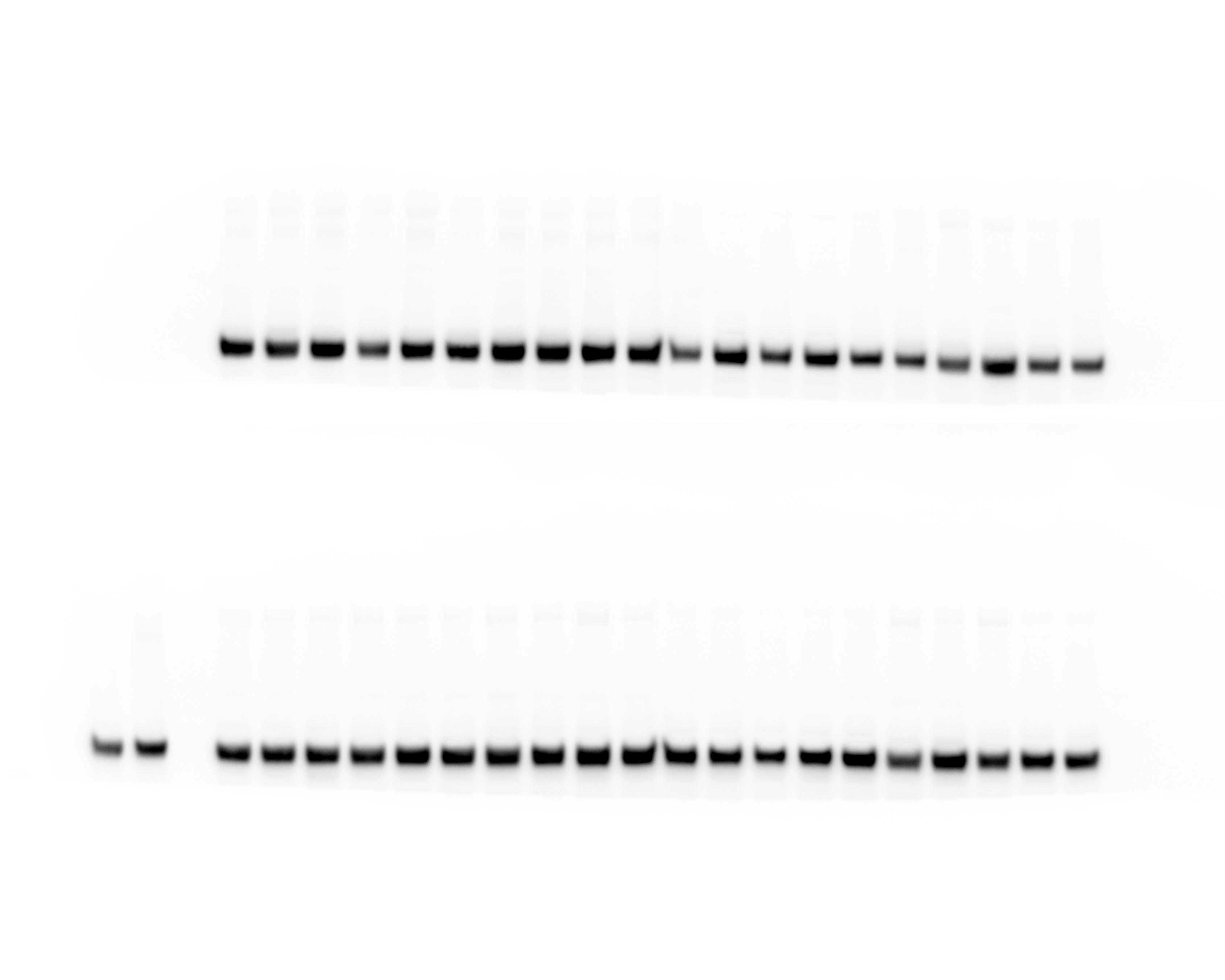

Supplement: Figure 5—source data 1. [file elife-90419-fig5-data1.zip › Fig5_raw images/Fig5A dKO F M tAKT.jpg]

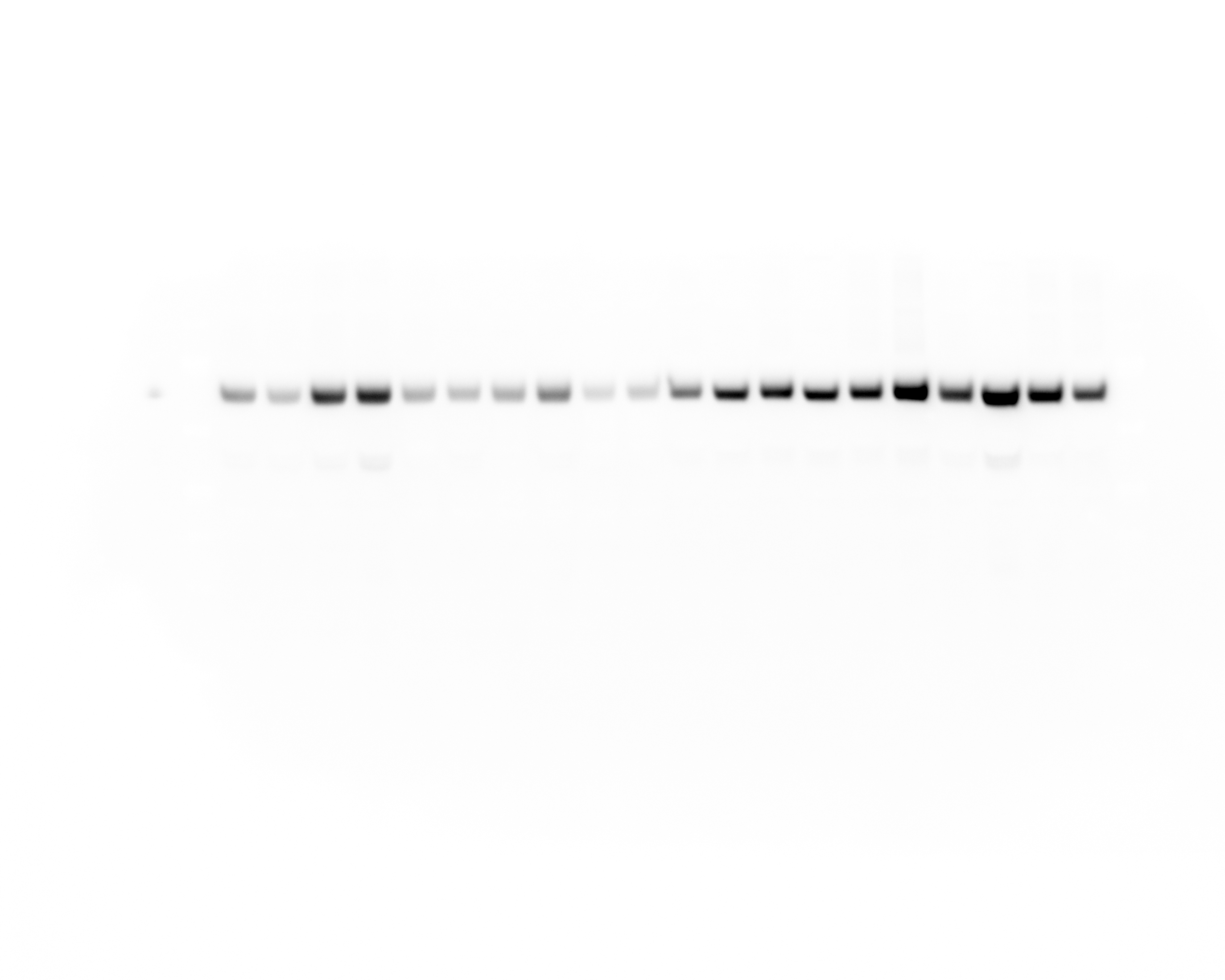

Supplement: Figure 5—source data 1. [file elife-90419-fig5-data1.zip › Fig5_raw images/Fig5A dKO F pAKT.jpg]

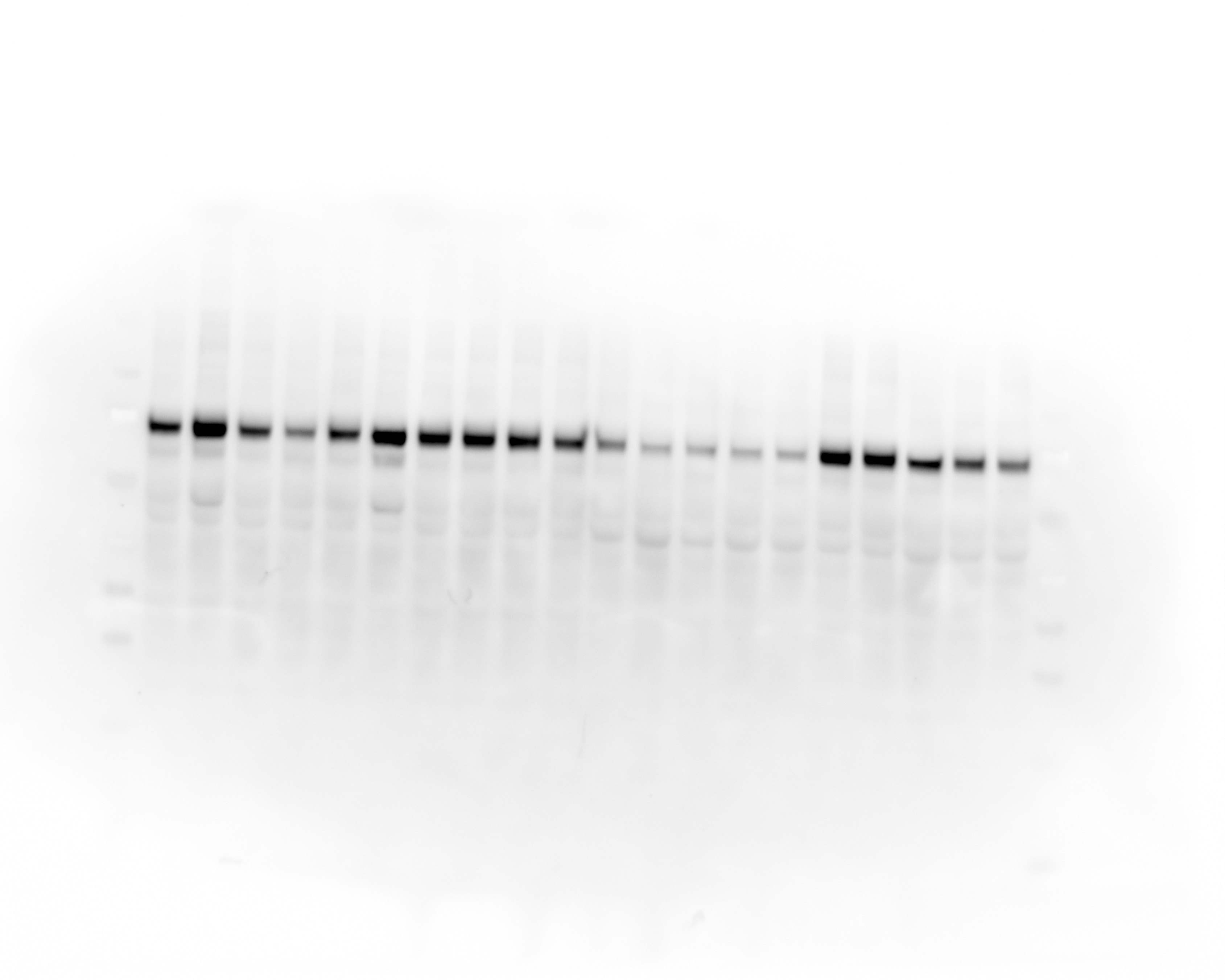

Supplement: Figure 5—source data 1. [file elife-90419-fig5-data1.zip › Fig5_raw images/Fig5A dKO F pAMPK.jpg]

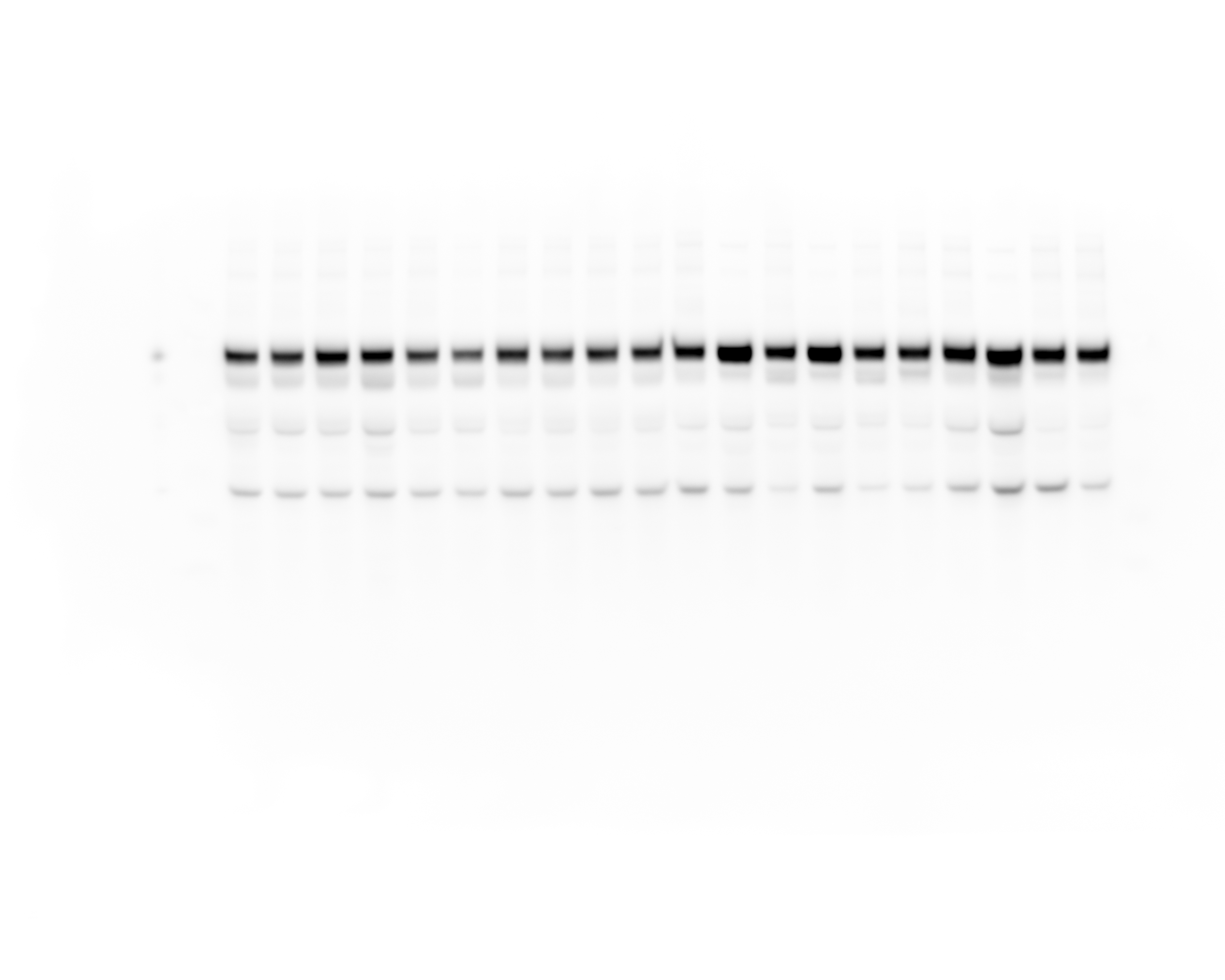

Supplement: Figure 5—source data 1. [file elife-90419-fig5-data1.zip › Fig5_raw images/Fig5A dKO F tAMPK.jpg]

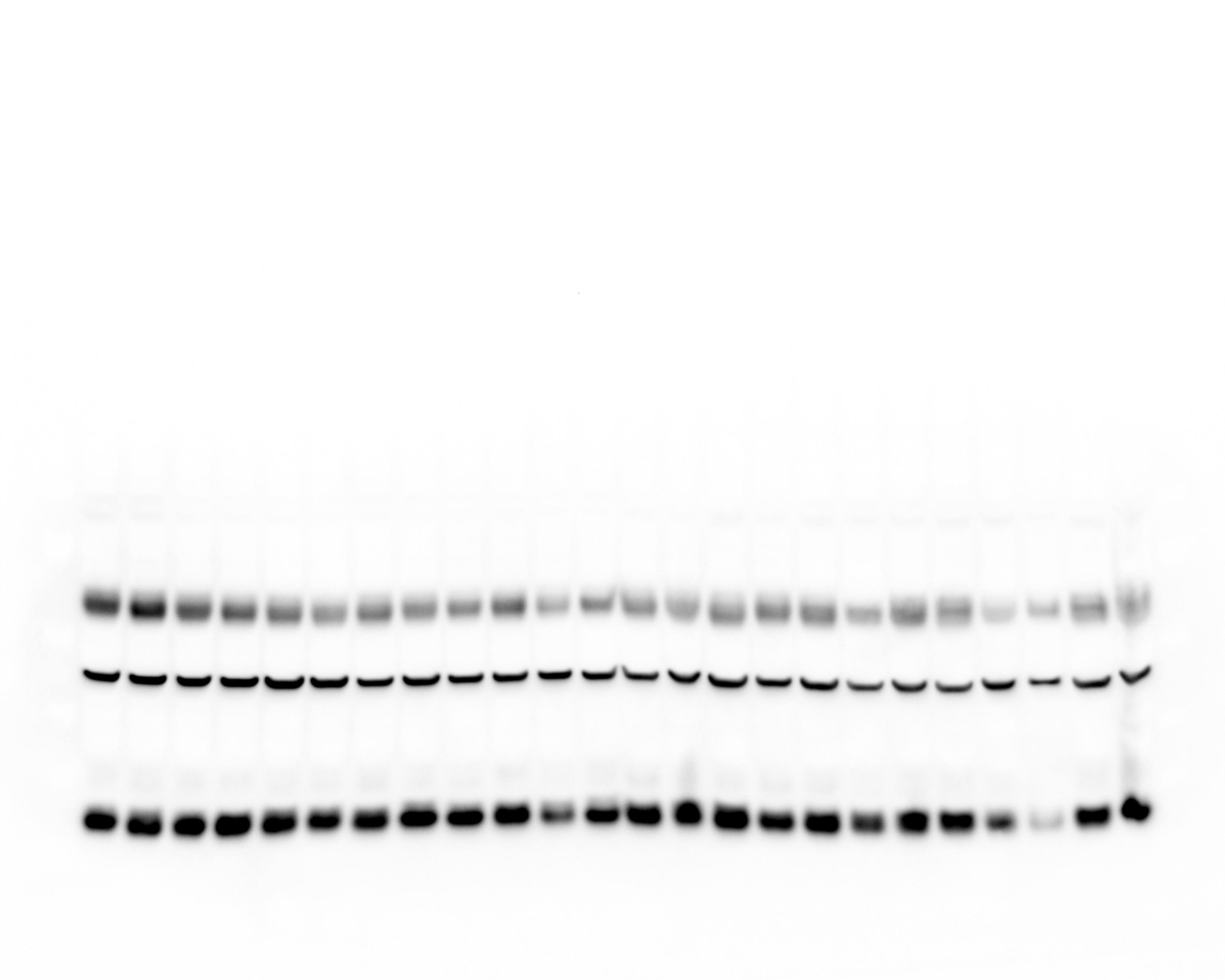

Supplement: Figure 5—source data 1. [file elife-90419-fig5-data1.zip › Fig5_raw images/Fig5D HFFD F Bactin.jpg]

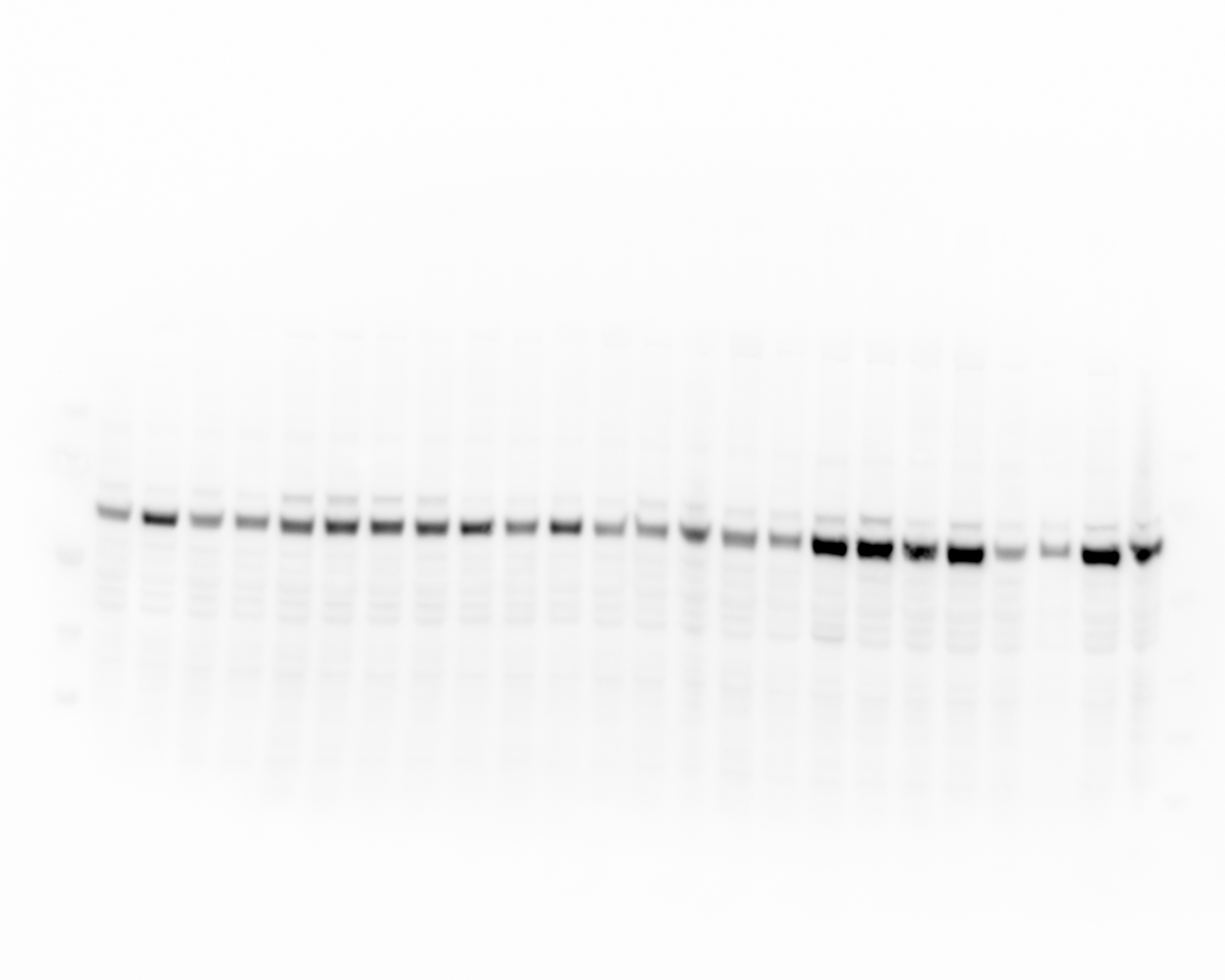

Supplement: Figure 5—source data 1. [file elife-90419-fig5-data1.zip › Fig5_raw images/Fig5D HFFD F pAKT.jpg]

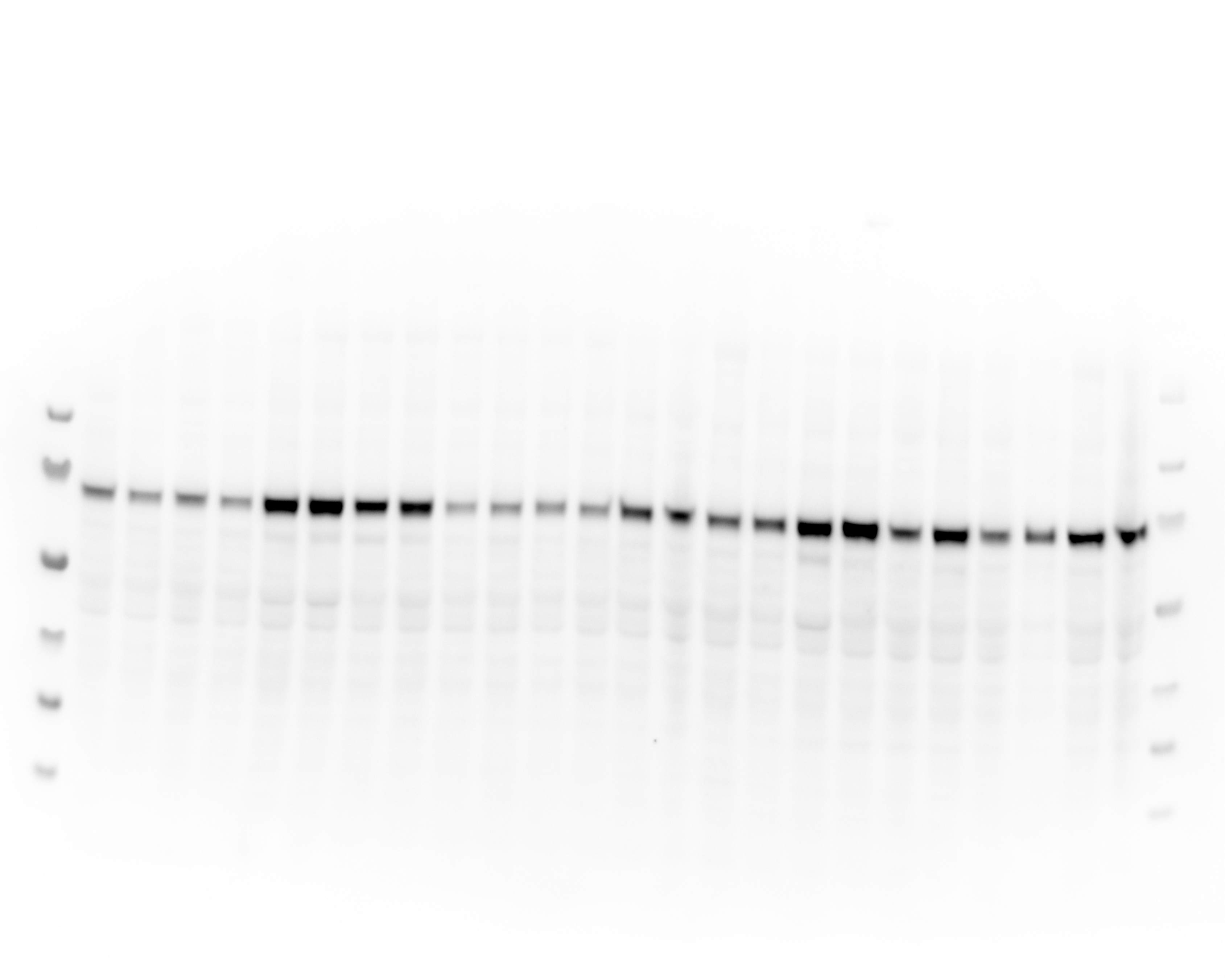

Supplement: Figure 5—source data 1. [file elife-90419-fig5-data1.zip › Fig5_raw images/Fig5D HFFD F pAMPK.jpg]

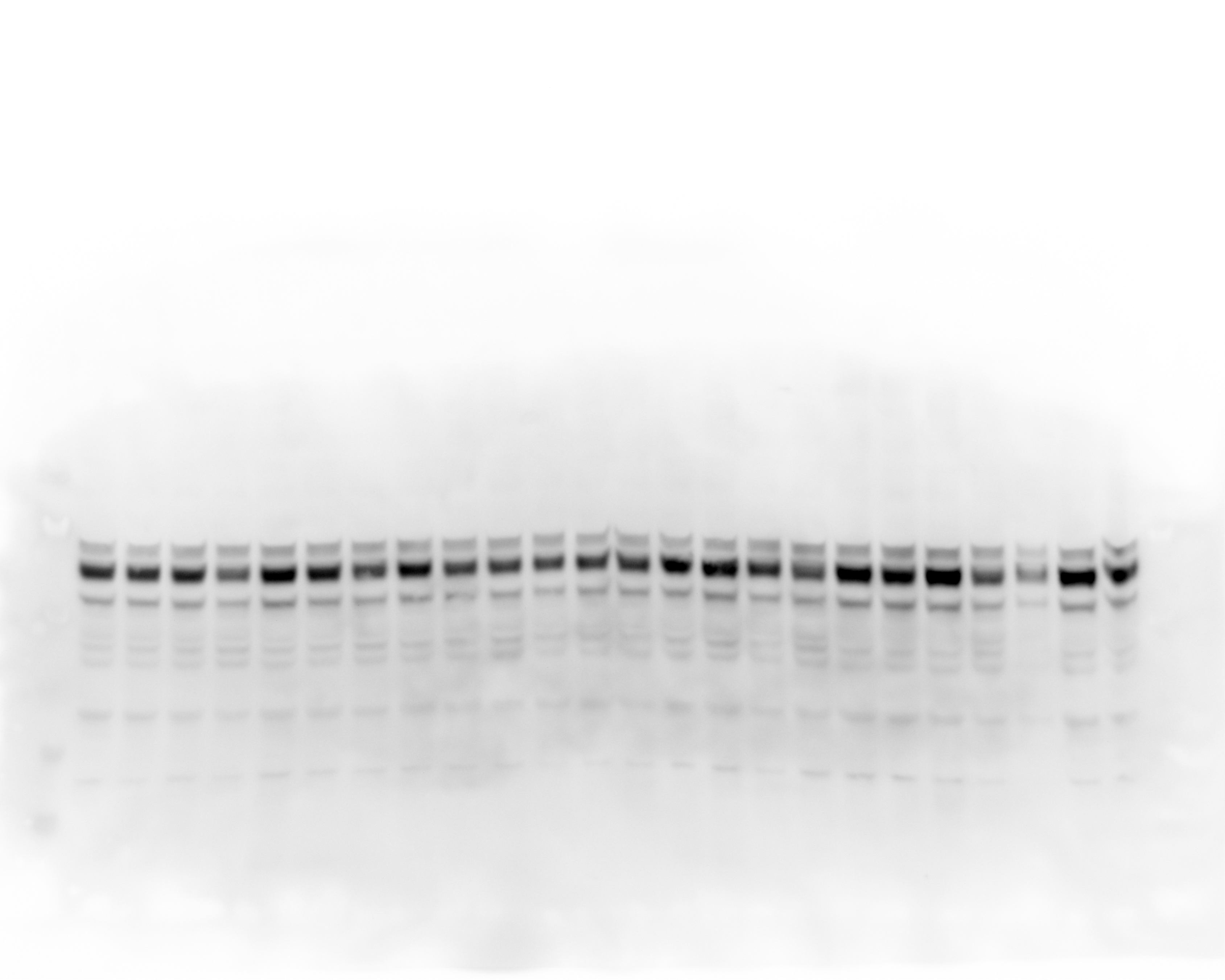

Supplement: Figure 5—source data 1. [file elife-90419-fig5-data1.zip › Fig5_raw images/Fig5D HFFD F tAKT.jpg]

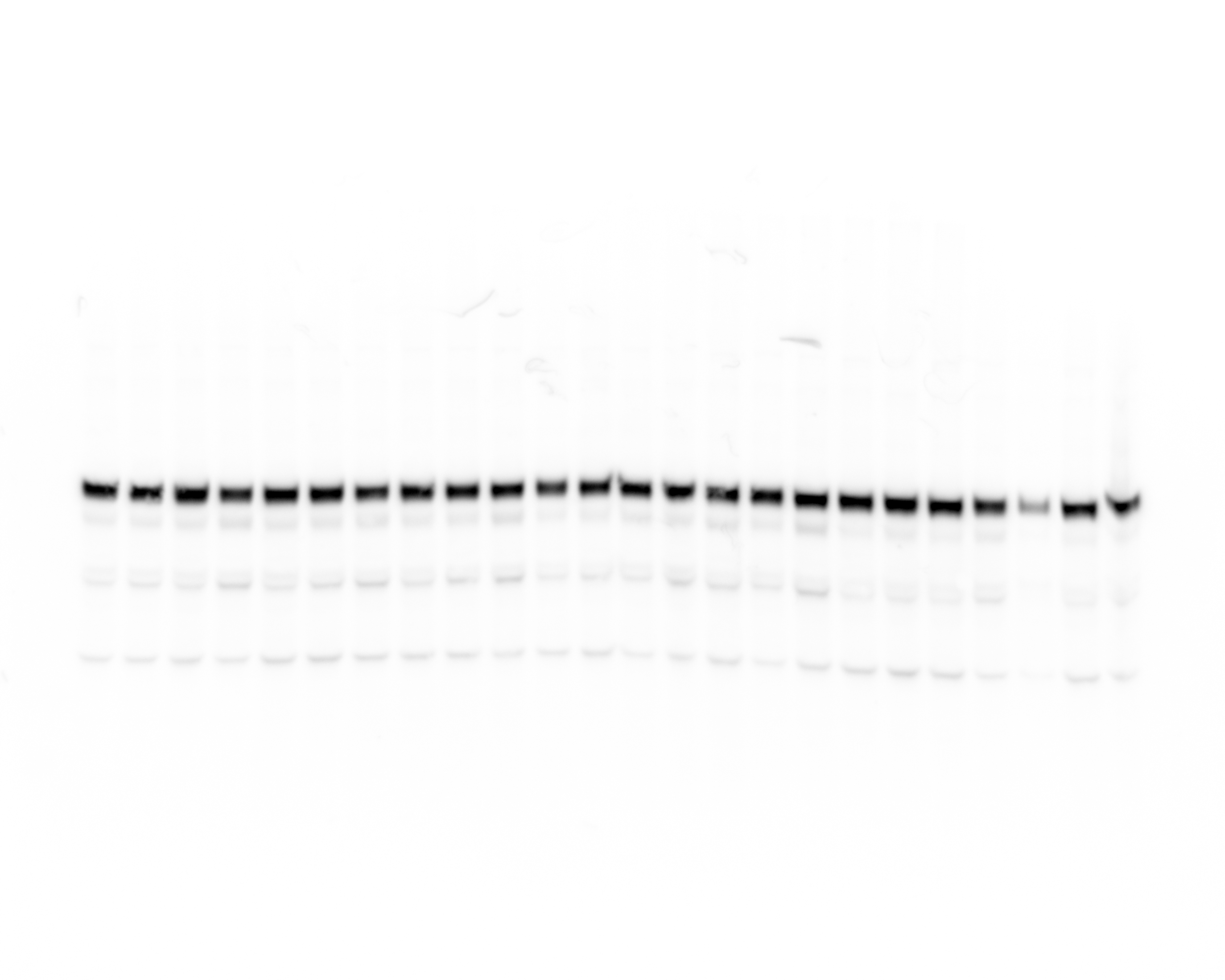

Supplement: Figure 5—source data 1. [file elife-90419-fig5-data1.zip › Fig5_raw images/Fig5D HFFD F tAMPK.jpg]

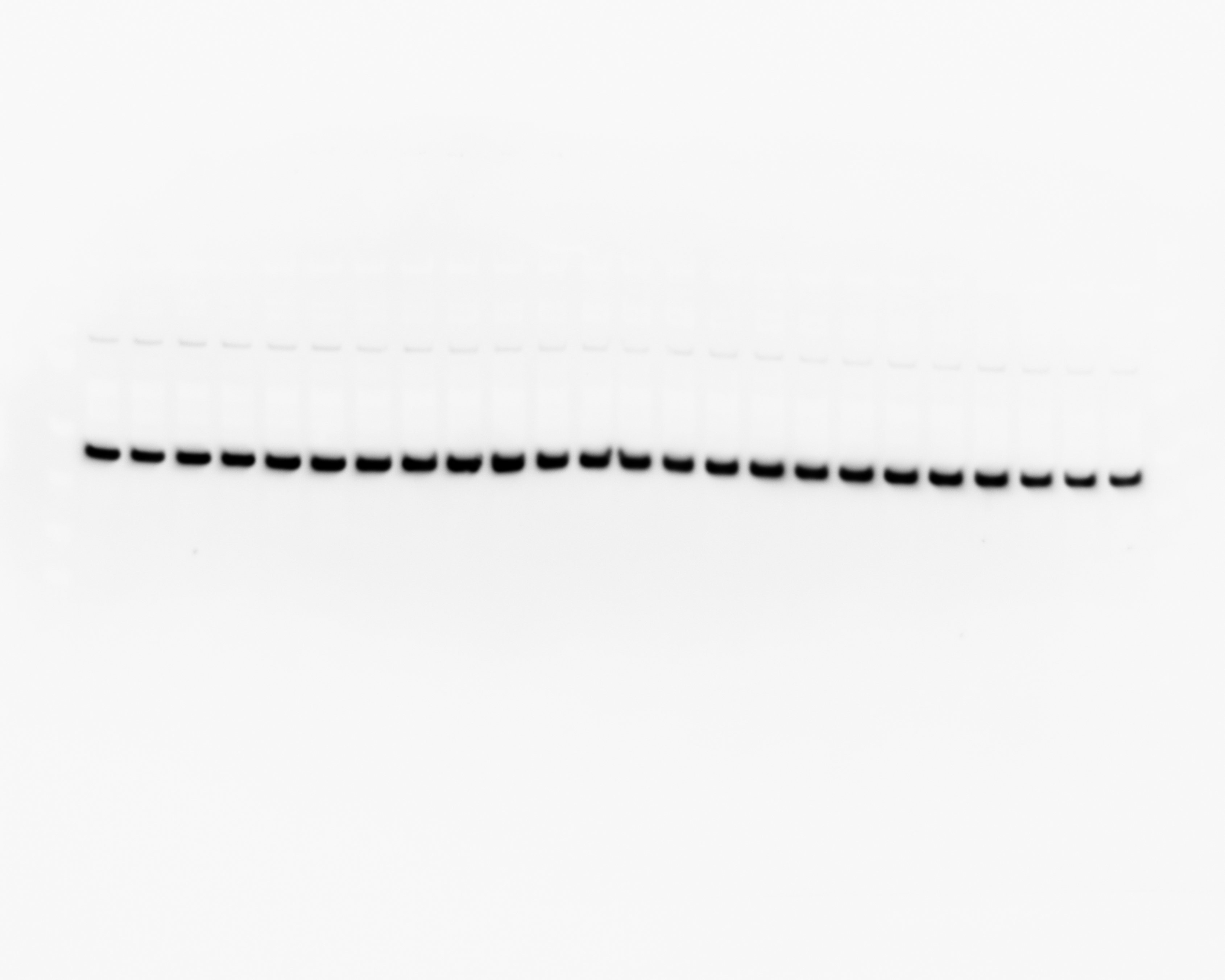

Supplement: Figure 5—source data 1. [file elife-90419-fig5-data1.zip › Fig5_raw images/Fig5G Bactin.jpg]

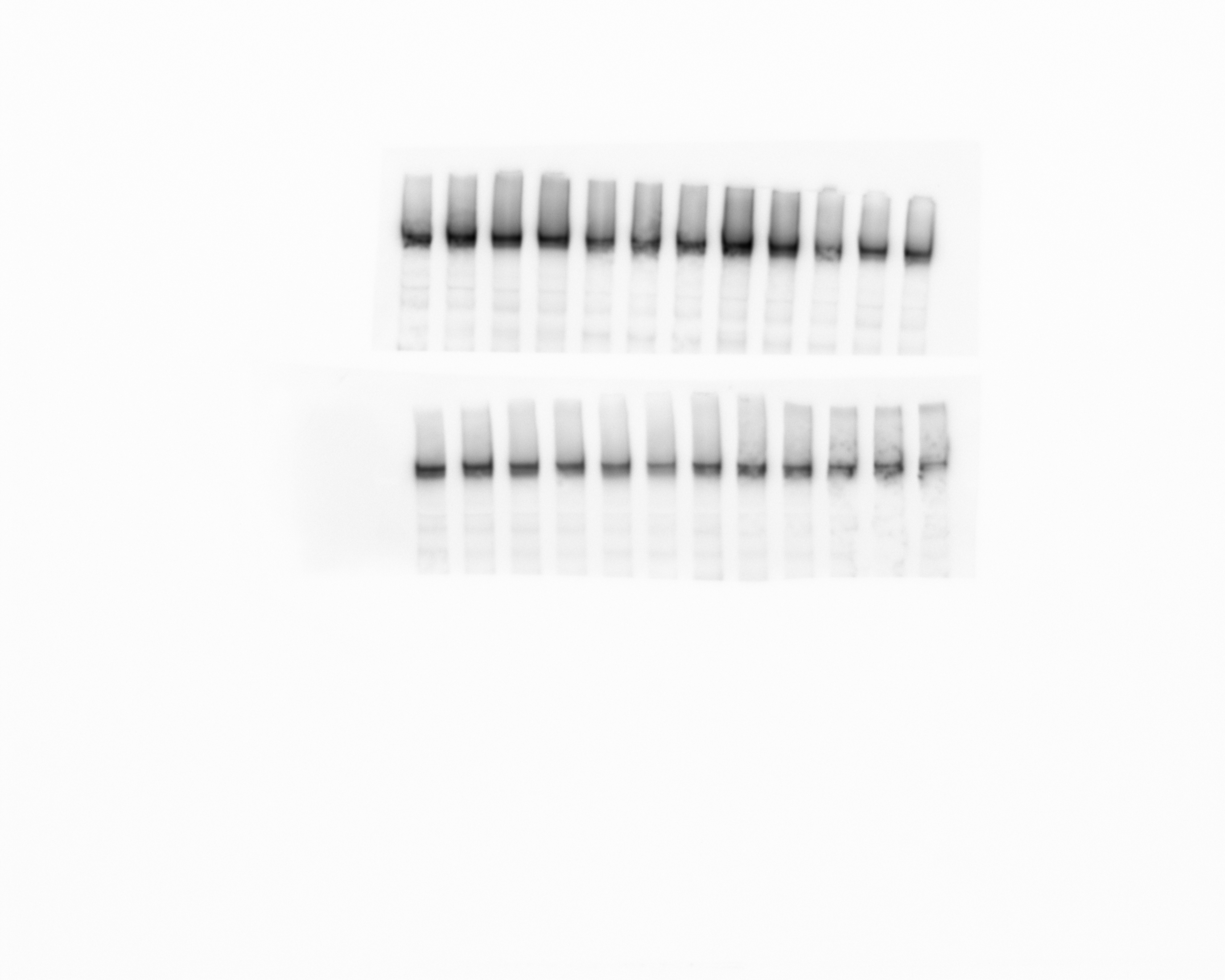

Supplement: Figure 5—source data 1. [file elife-90419-fig5-data1.zip › Fig5_raw images/Fig5G pACC.jpg]

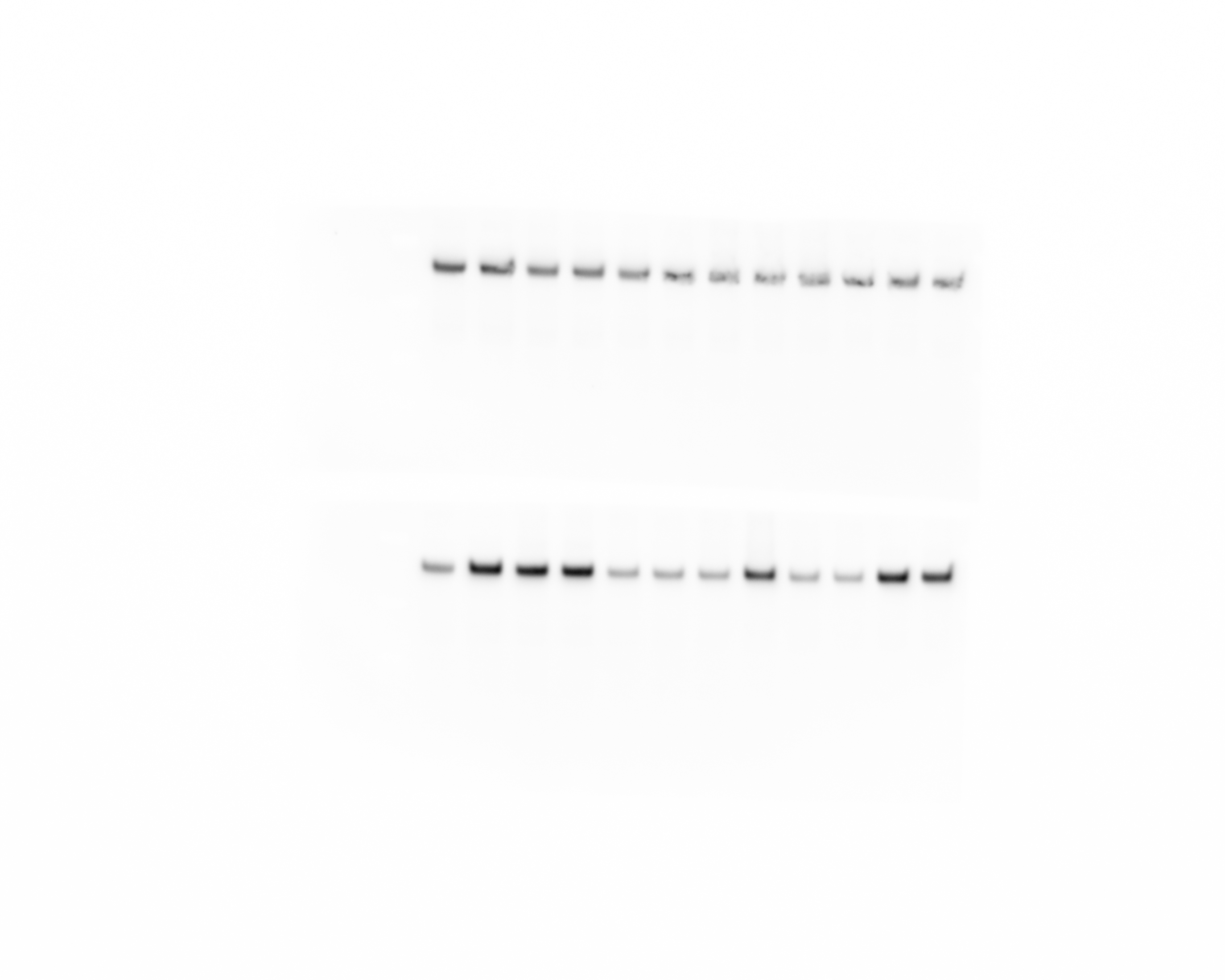

Supplement: Figure 5—source data 1. [file elife-90419-fig5-data1.zip › Fig5_raw images/Fig5G pAKT.jpg]

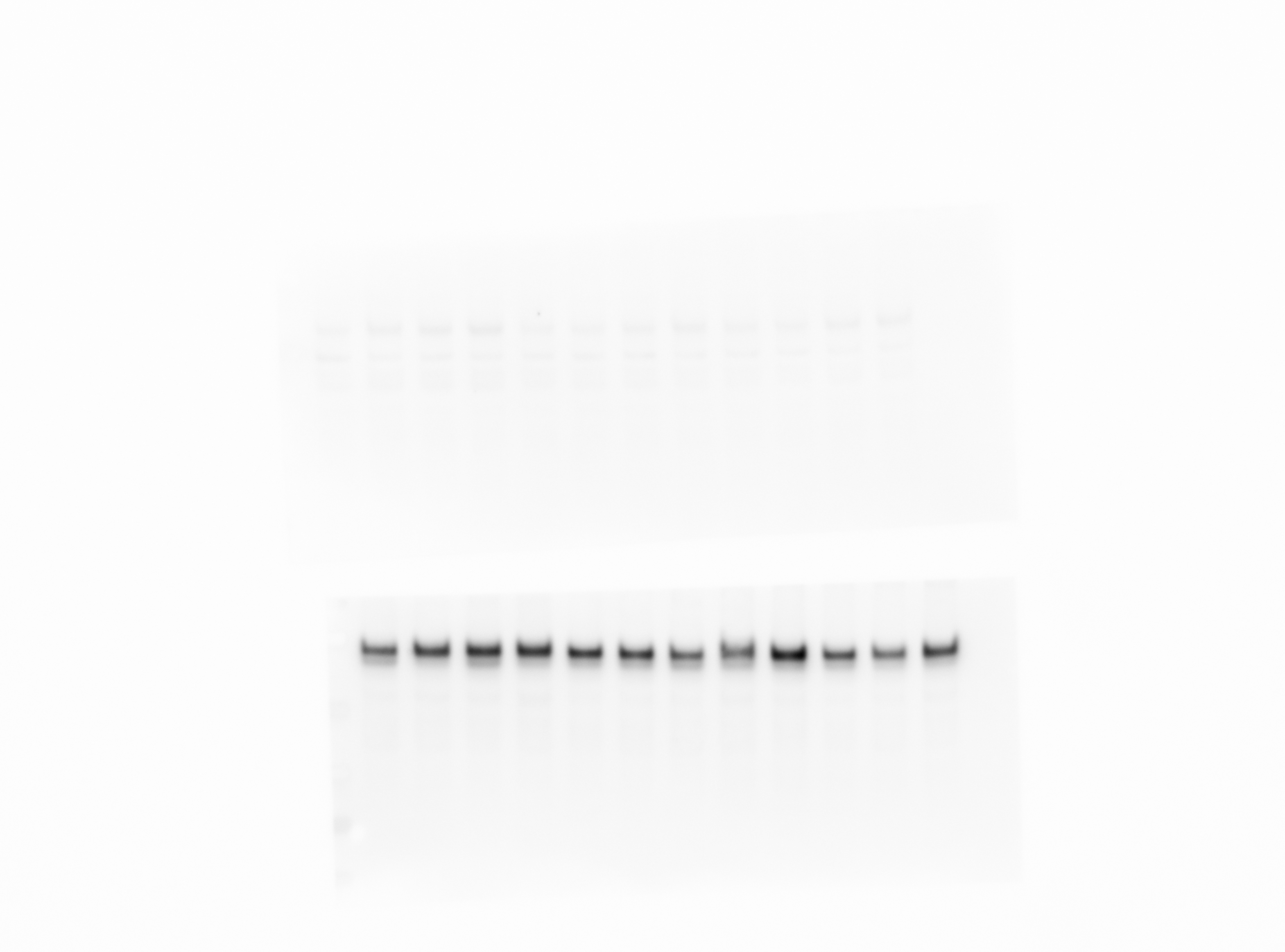

Supplement: Figure 5—source data 1. [file elife-90419-fig5-data1.zip › Fig5_raw images/Fig5G pAMPK.jpg]

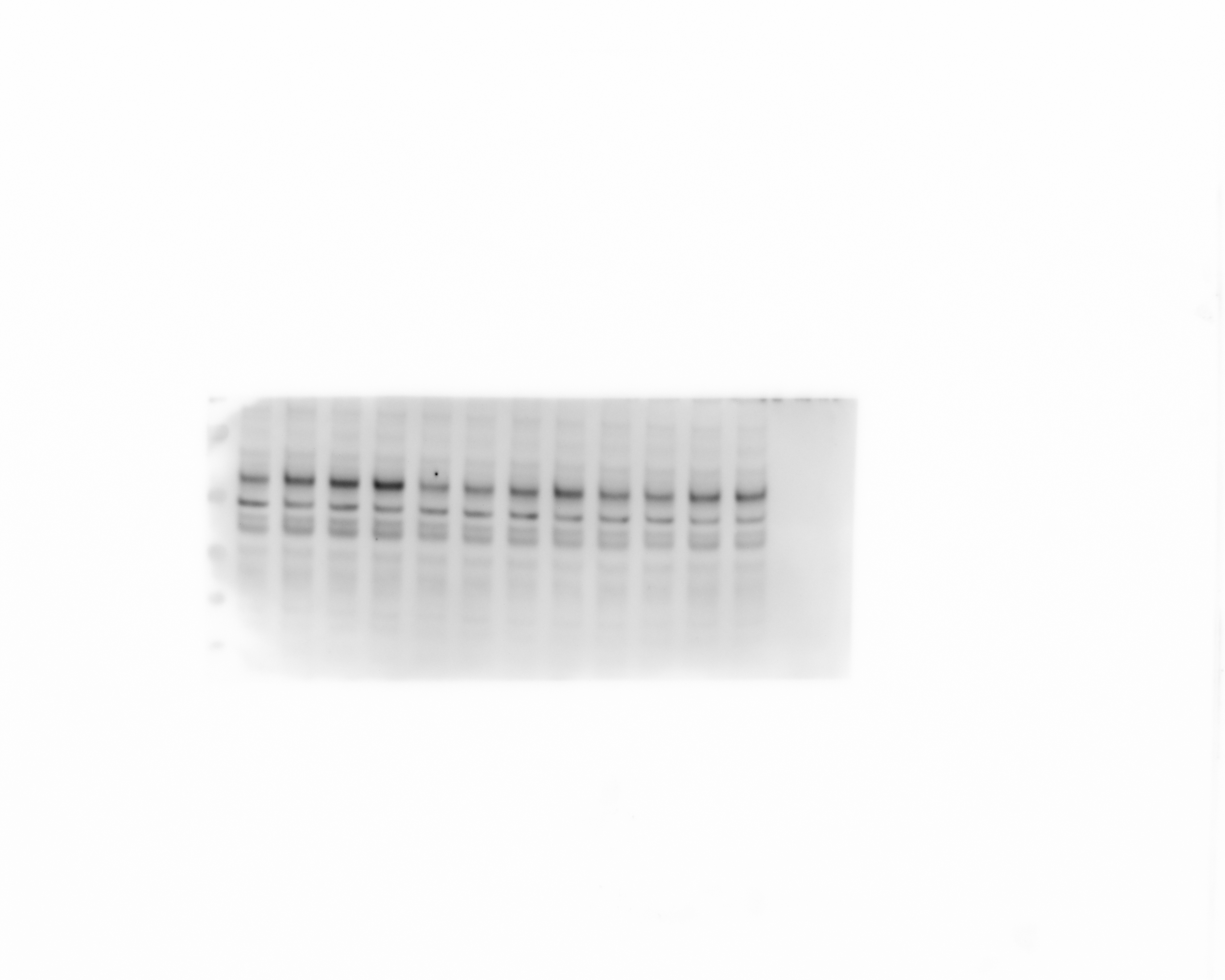

Supplement: Figure 5—source data 1. [file elife-90419-fig5-data1.zip › Fig5_raw images/Fig5G pLKB1.jpg]

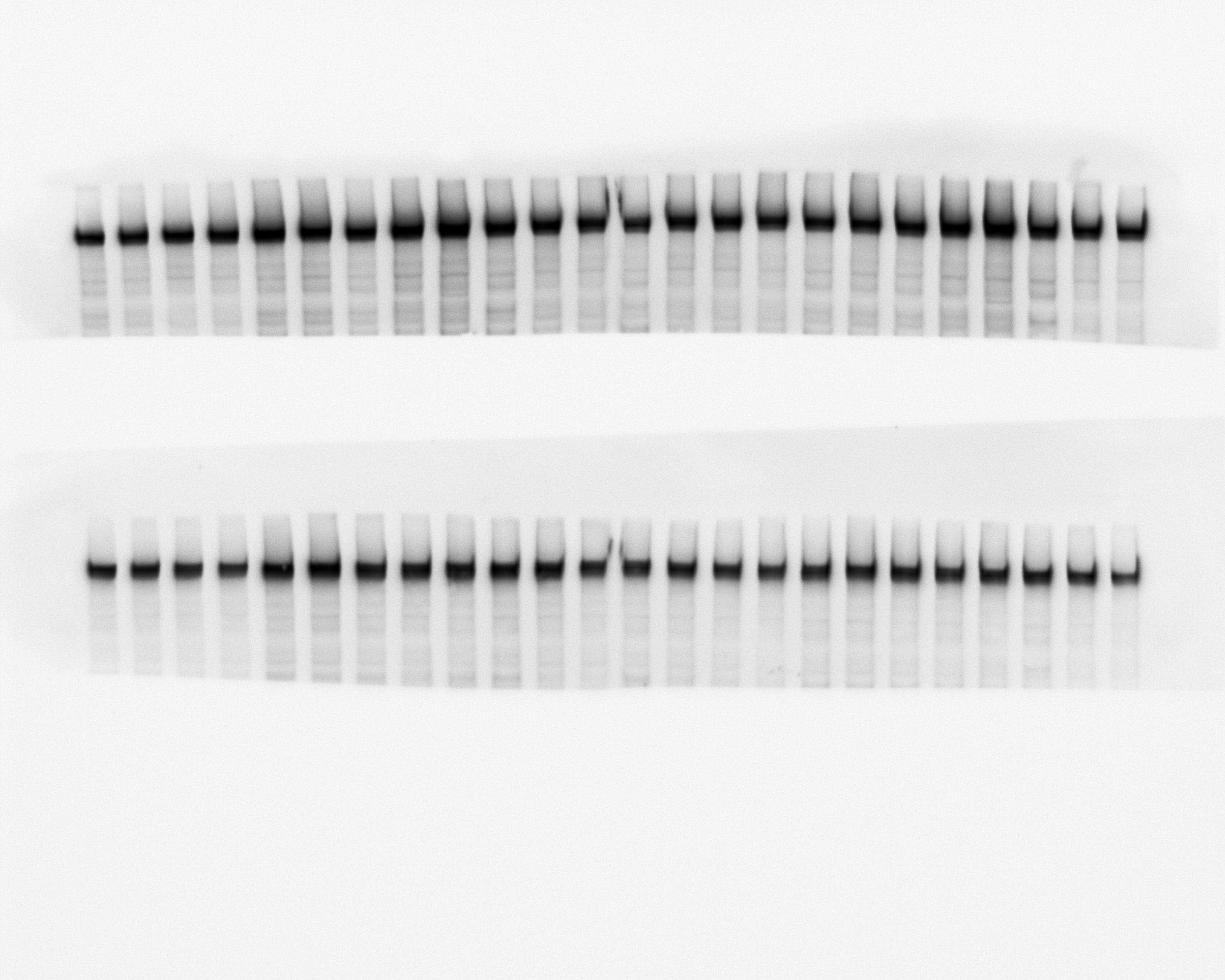

Supplement: Figure 5—source data 1. [file elife-90419-fig5-data1.zip › Fig5_raw images/Fig5G tACC.jpg]

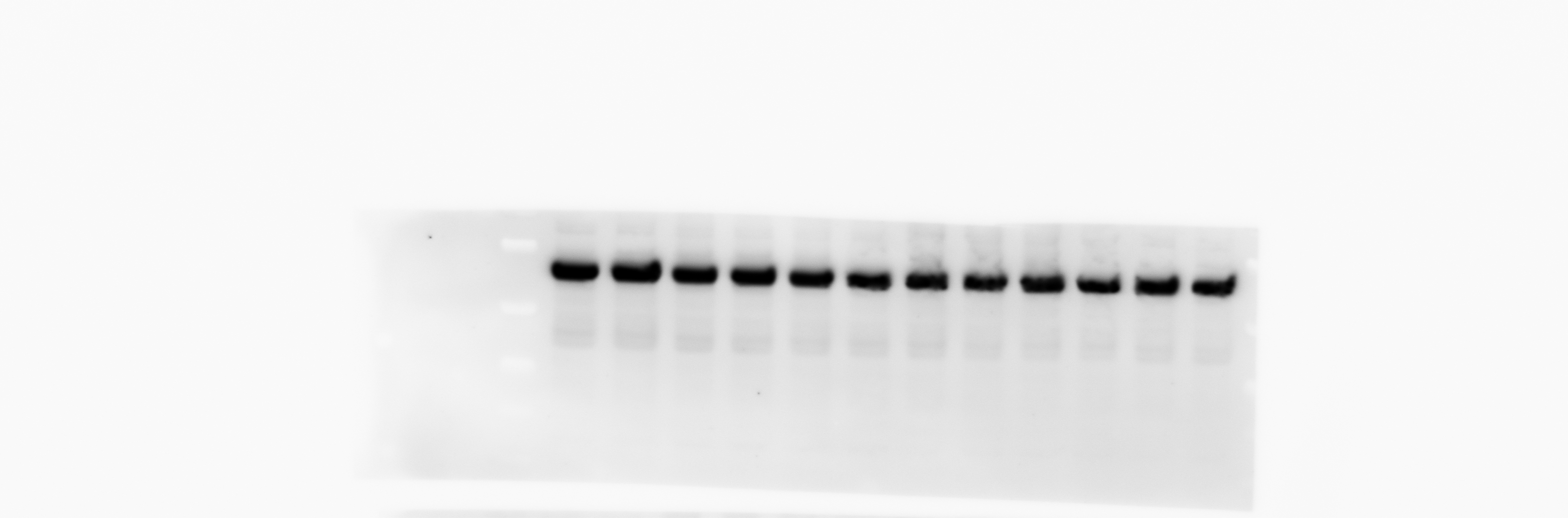

Supplement: Figure 5—source data 1. [file elife-90419-fig5-data1.zip › Fig5_raw images/Fig5G tAKT.jpg]

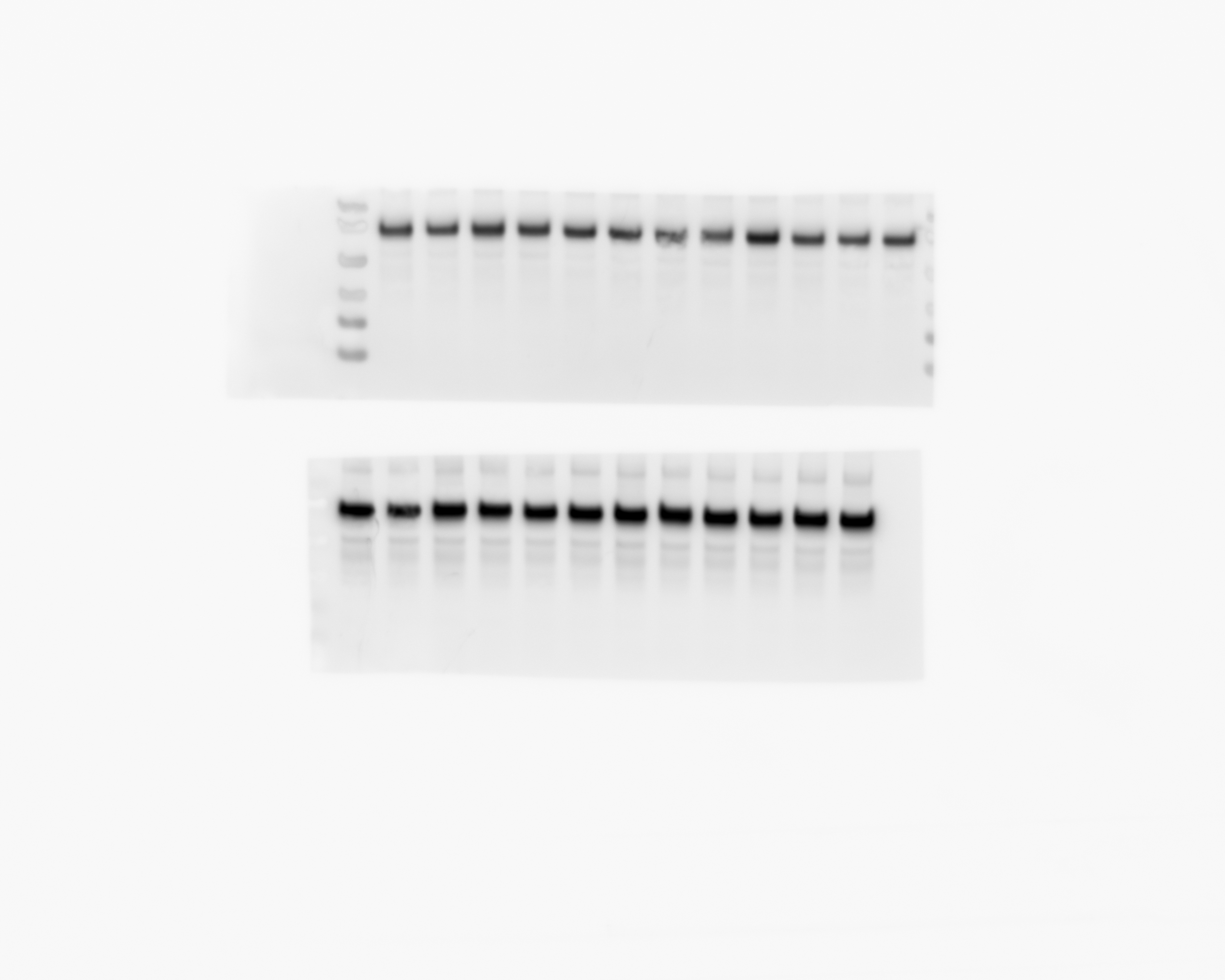

Supplement: Figure 5—source data 1. [file elife-90419-fig5-data1.zip › Fig5_raw images/Fig5G tAMPK.jpg]

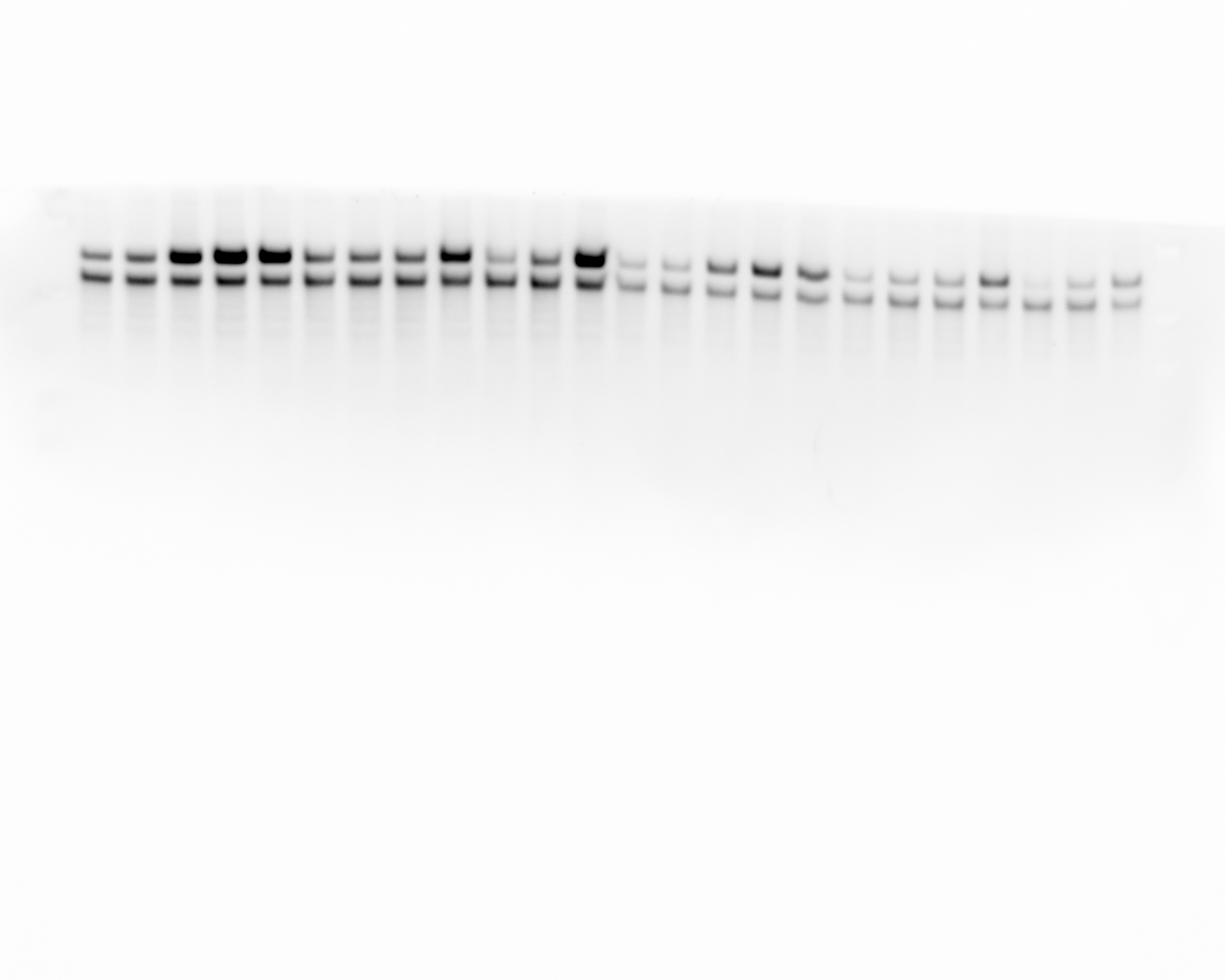

Supplement: Figure 5—source data 1. [file elife-90419-fig5-data1.zip › Fig5_raw images/Fig5G tLKB1.jpg]

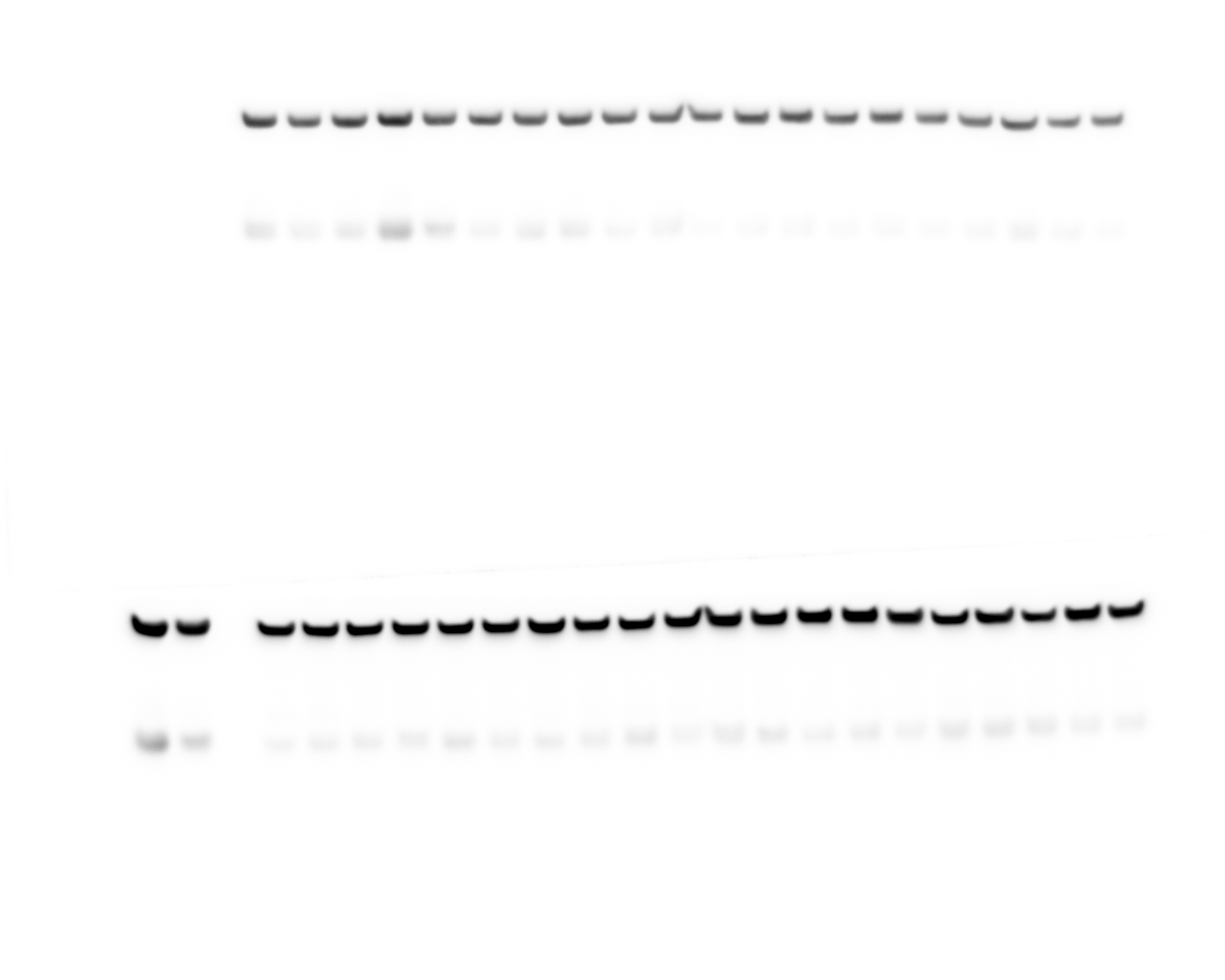

Supplement: Figure 5—figure supplement 1—source data 1. [file elife-90419-fig5-figsupp1-data1.zip › Figure 5-figure supplement 1_raw images/Fig5s1 dKO F M Bactin.jpg]

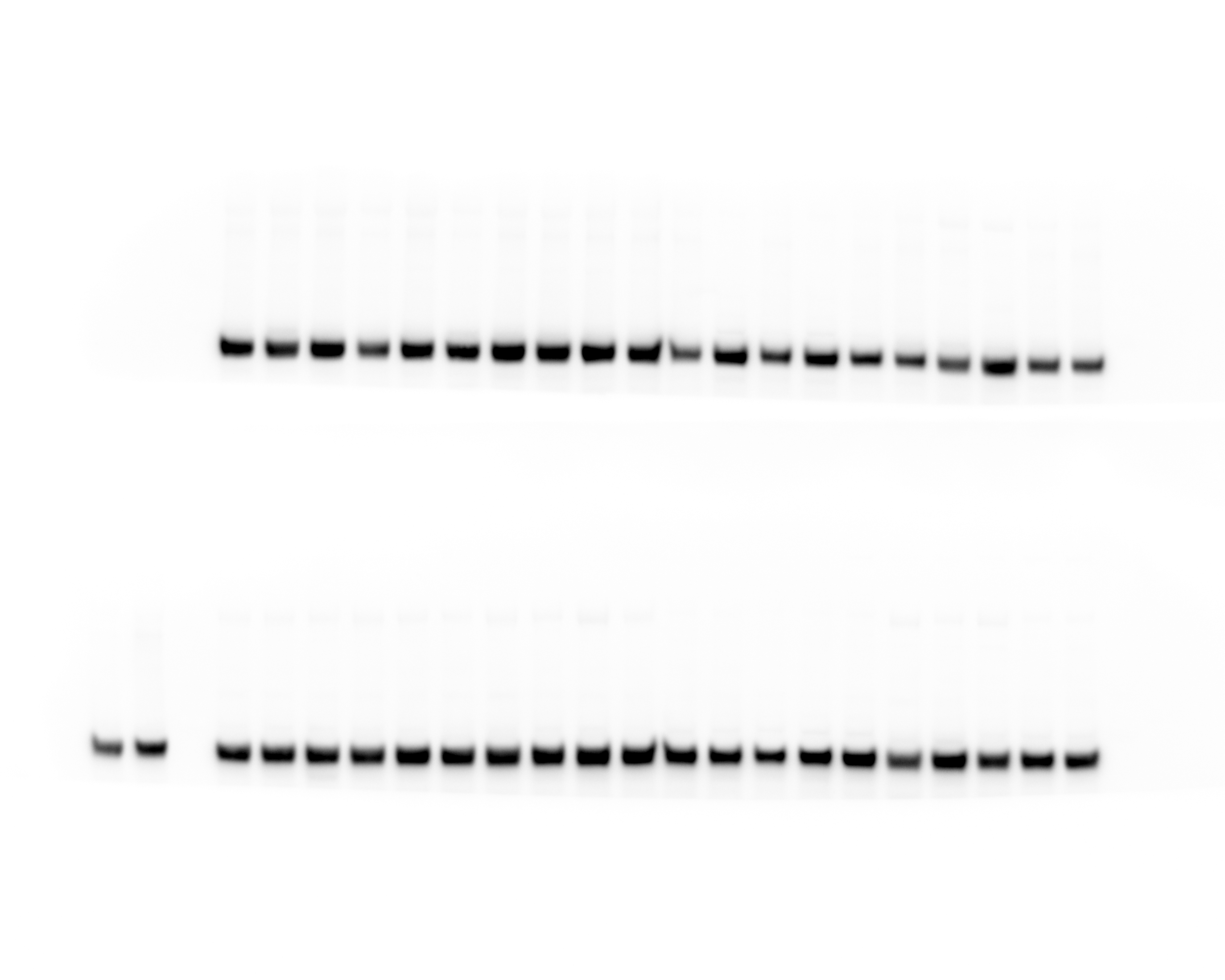

Supplement: Figure 5—figure supplement 1—source data 1. [file elife-90419-fig5-figsupp1-data1.zip › Figure 5-figure supplement 1_raw images/Fig5s1 dKO F M tAKT.jpg]

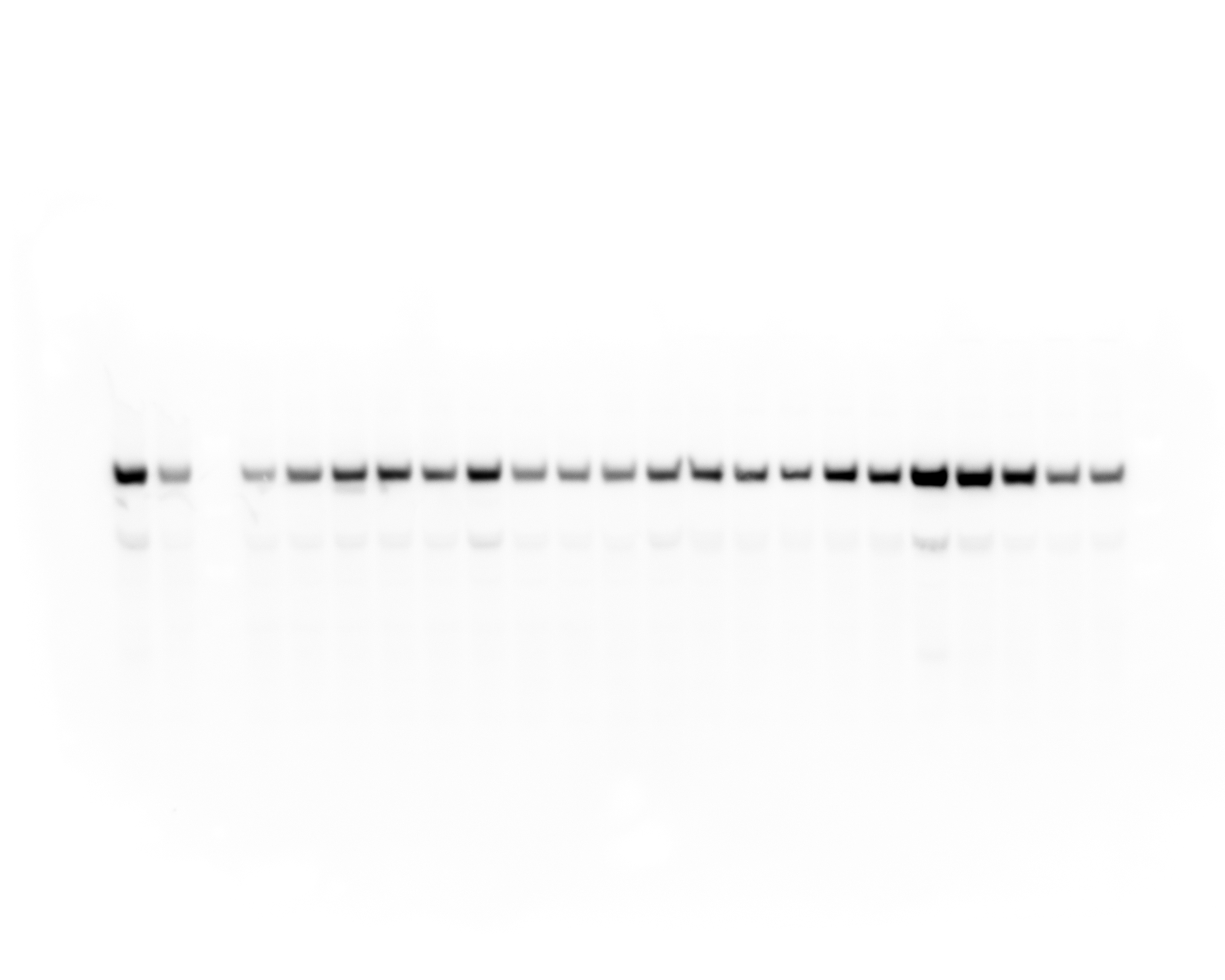

Supplement: Figure 5—figure supplement 1—source data 1. [file elife-90419-fig5-figsupp1-data1.zip › Figure 5-figure supplement 1_raw images/Fig5s1 dKO M pAKT.jpg]

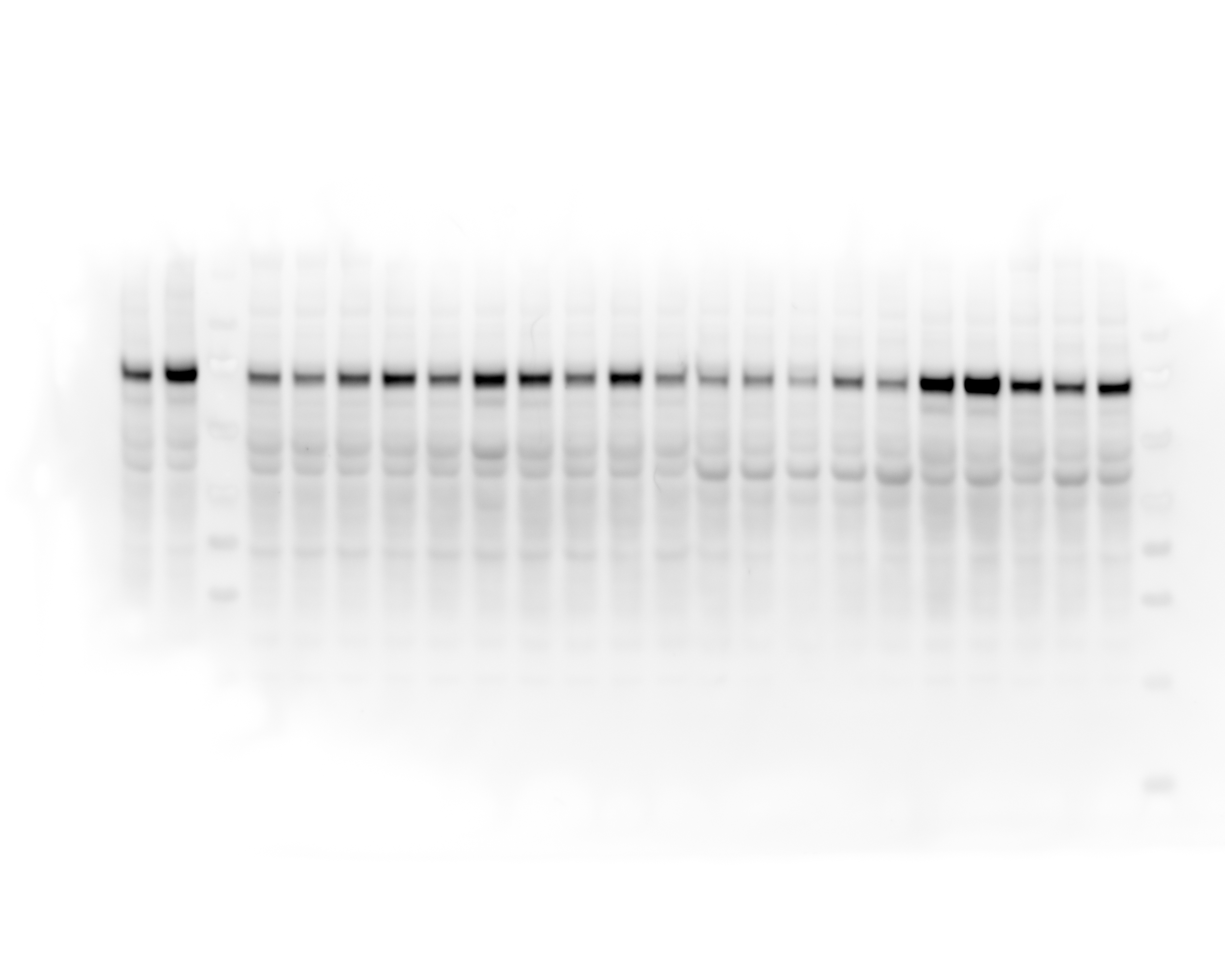

Supplement: Figure 5—figure supplement 1—source data 1. [file elife-90419-fig5-figsupp1-data1.zip › Figure 5-figure supplement 1_raw images/Fig5s1 dKO M pAMPK.jpg]

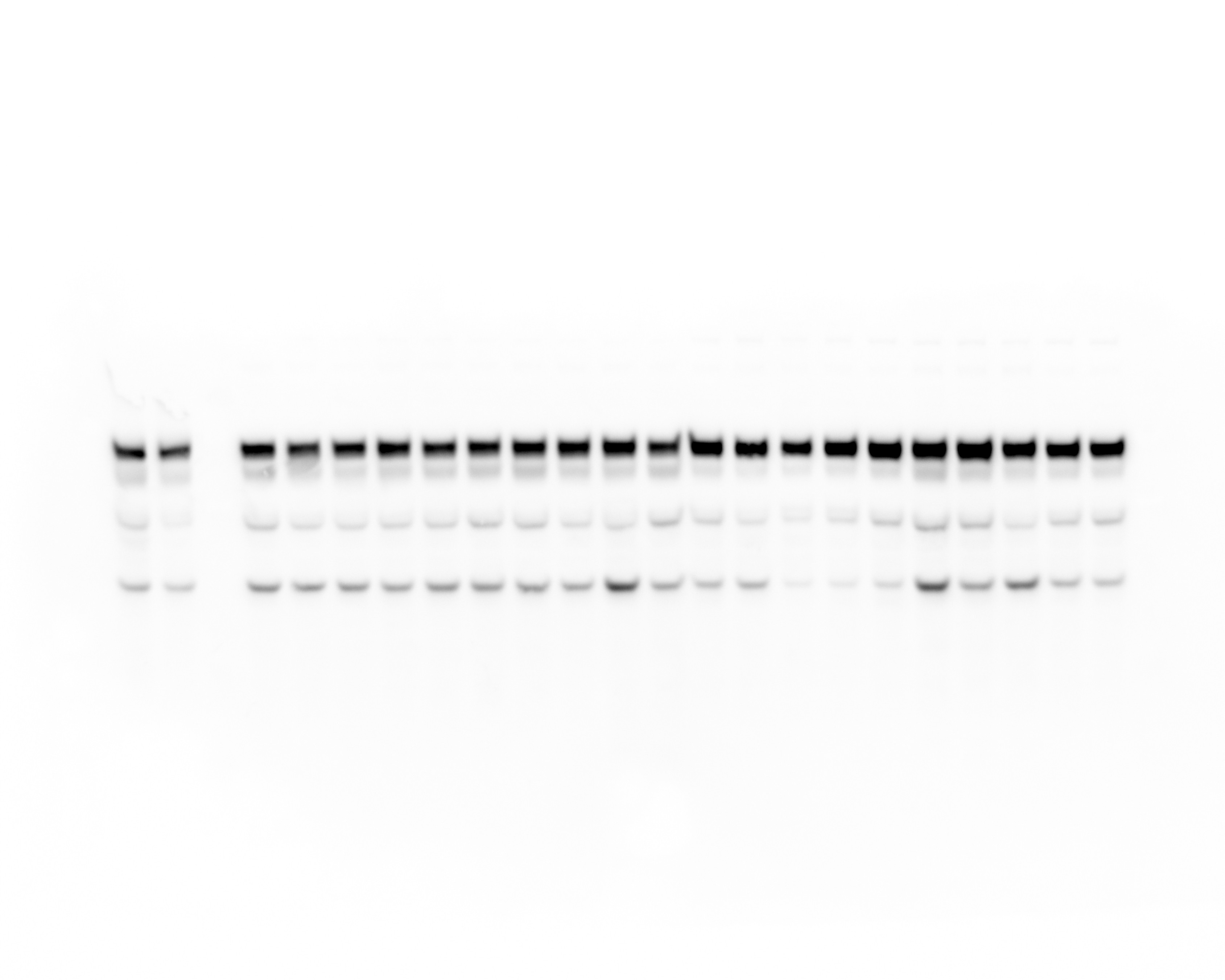

Supplement: Figure 5—figure supplement 1—source data 1. [file elife-90419-fig5-figsupp1-data1.zip › Figure 5-figure supplement 1_raw images/Fig5s1 dKO M tAMPK.jpg]

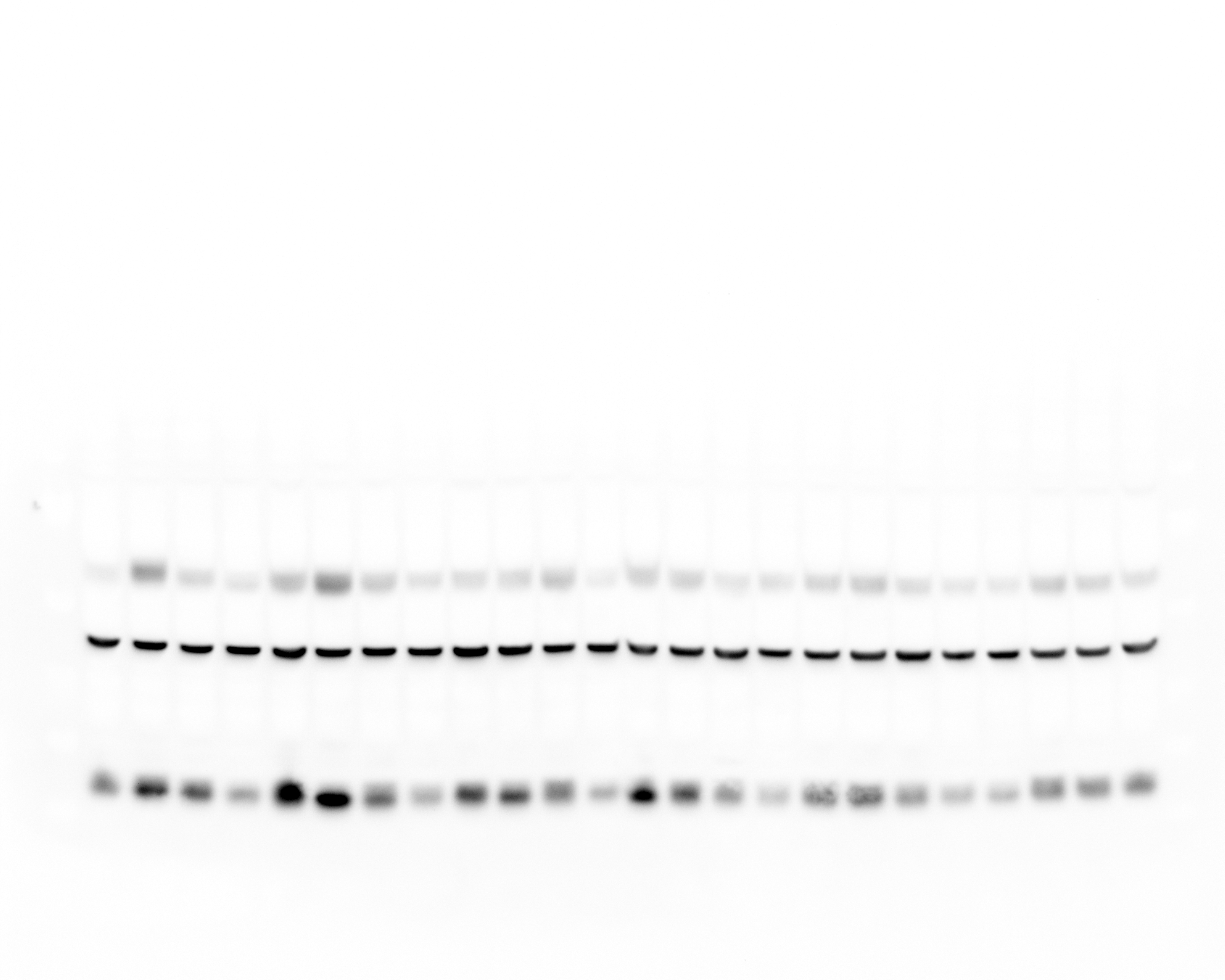

Supplement: Figure 5—figure supplement 1—source data 1. [file elife-90419-fig5-figsupp1-data1.zip › Figure 5-figure supplement 1_raw images/Fig5s1 HFFD M Bactin.jpg]

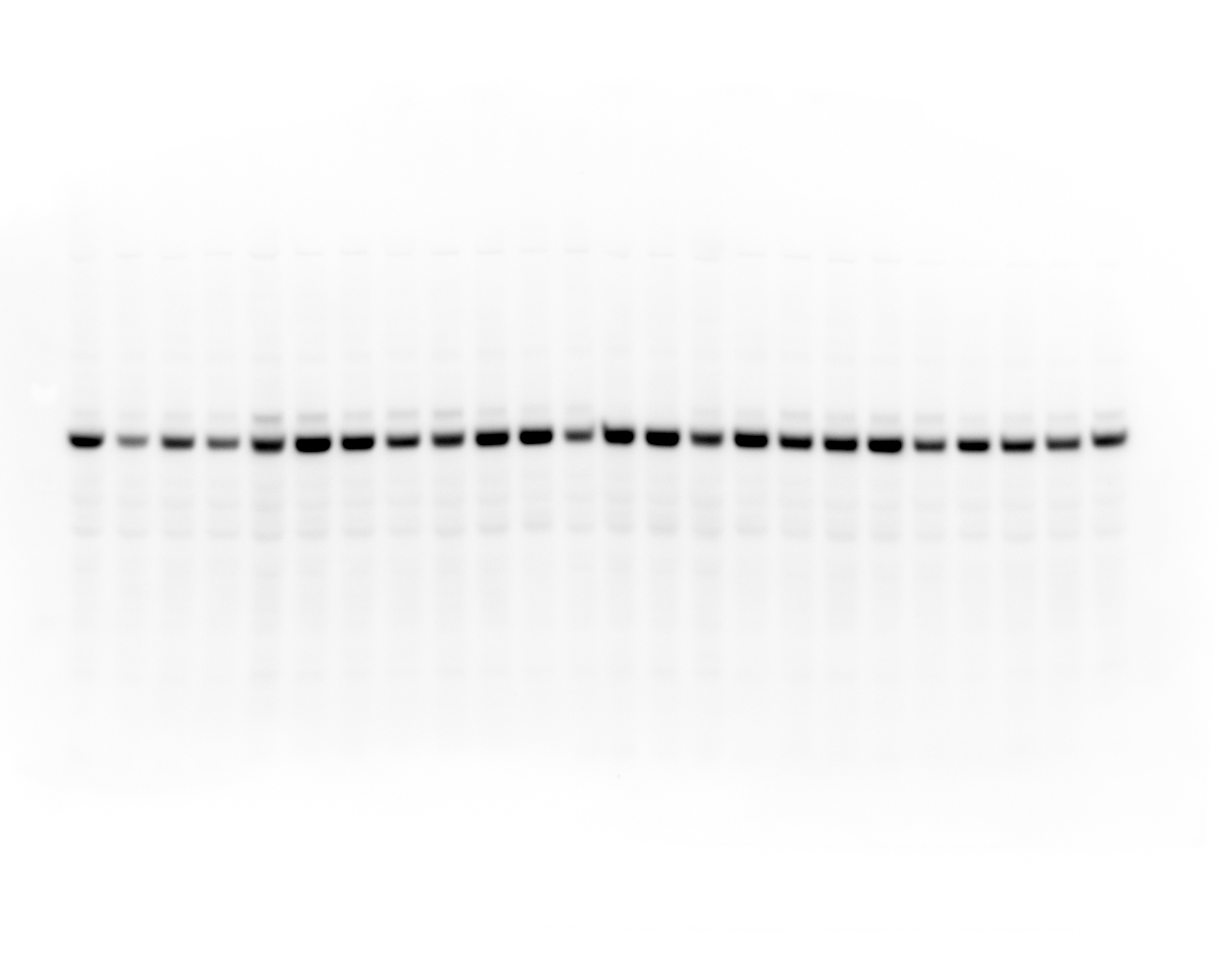

Supplement: Figure 5—figure supplement 1—source data 1. [file elife-90419-fig5-figsupp1-data1.zip › Figure 5-figure supplement 1_raw images/Fig5s1 HFFD M pAKT.jpg]

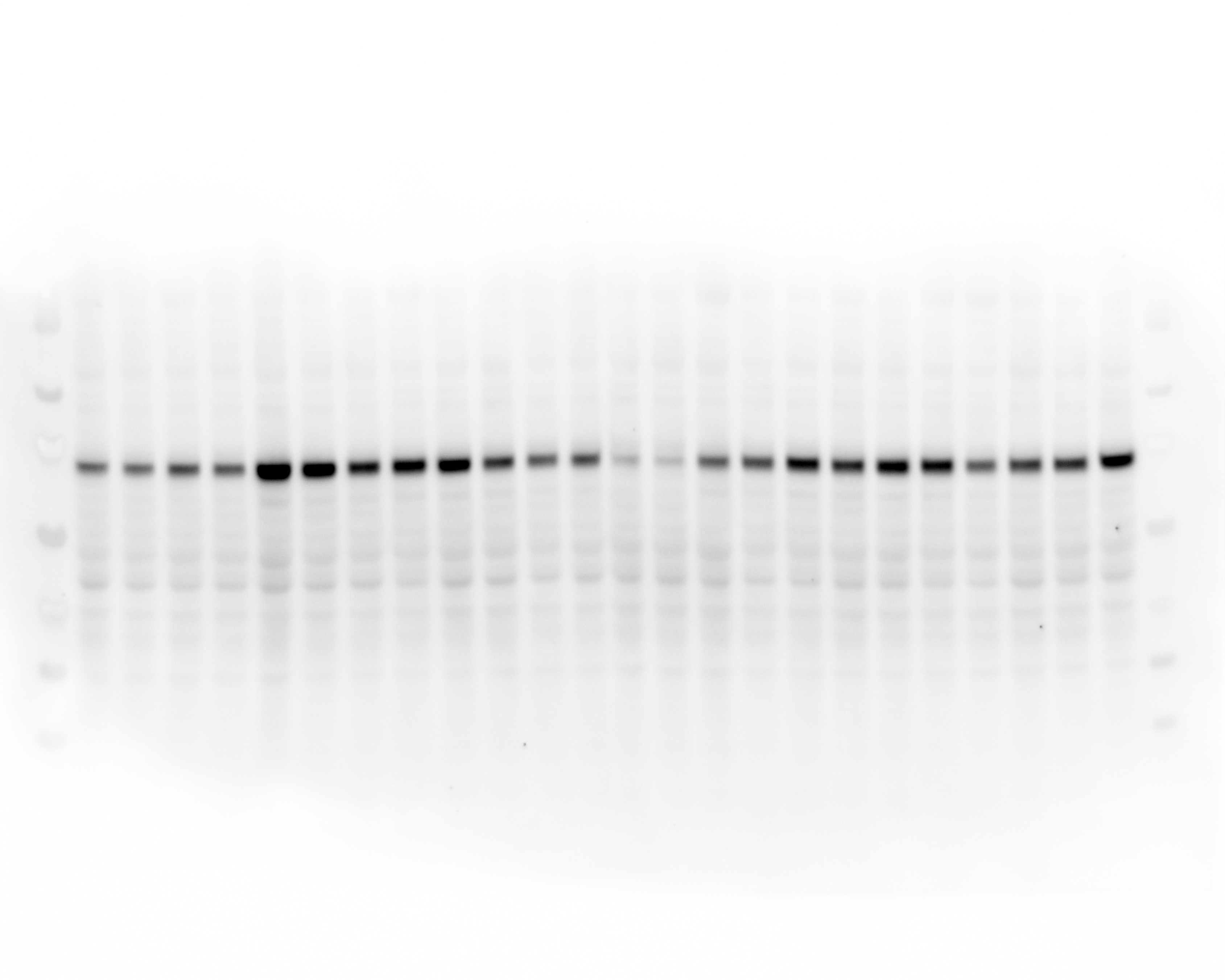

Supplement: Figure 5—figure supplement 1—source data 1. [file elife-90419-fig5-figsupp1-data1.zip › Figure 5-figure supplement 1_raw images/Fig5s1 HFFD M pAMPK.jpg]

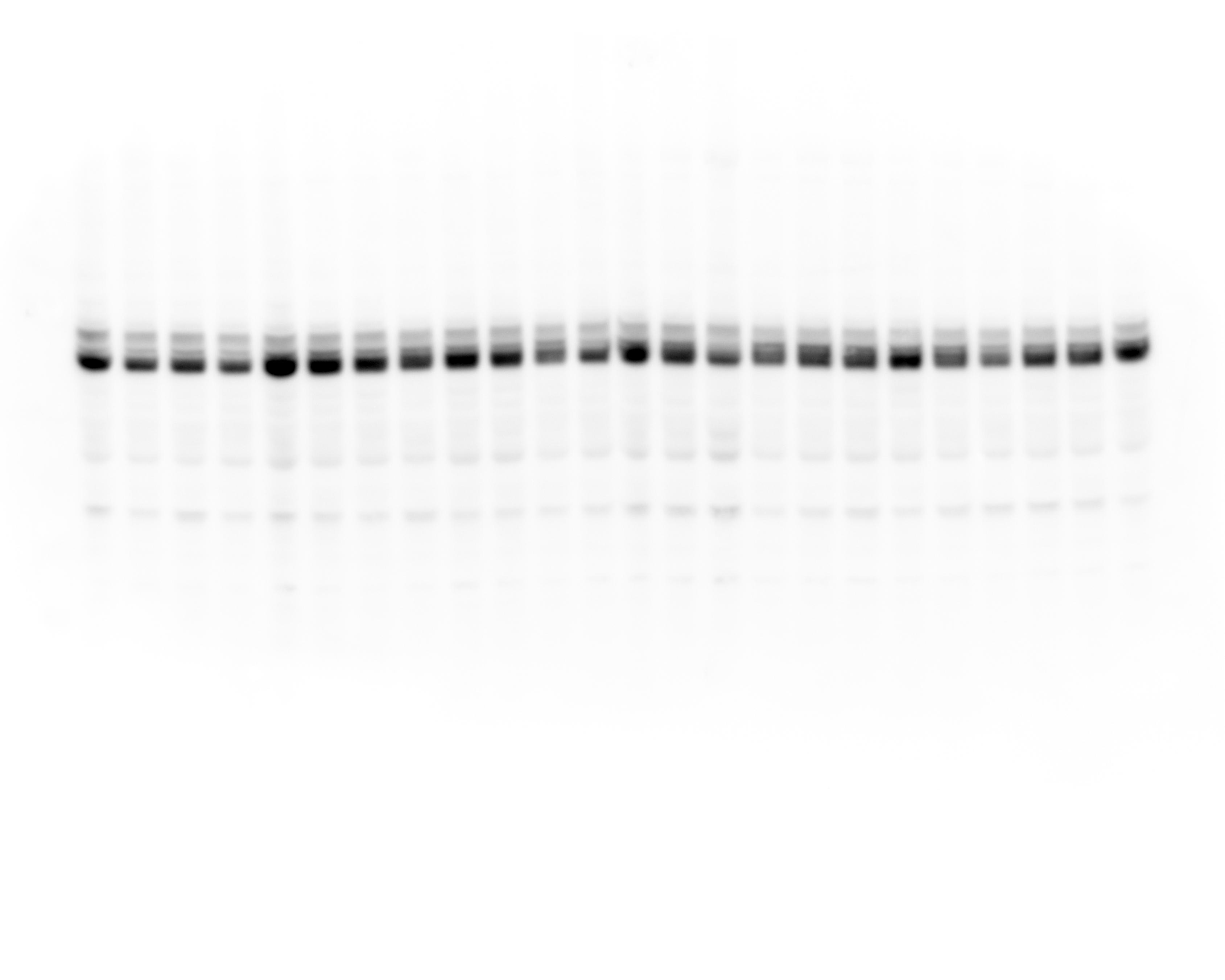

Supplement: Figure 5—figure supplement 1—source data 1. [file elife-90419-fig5-figsupp1-data1.zip › Figure 5-figure supplement 1_raw images/Fig5s1 HFFD M tAKT.jpg]

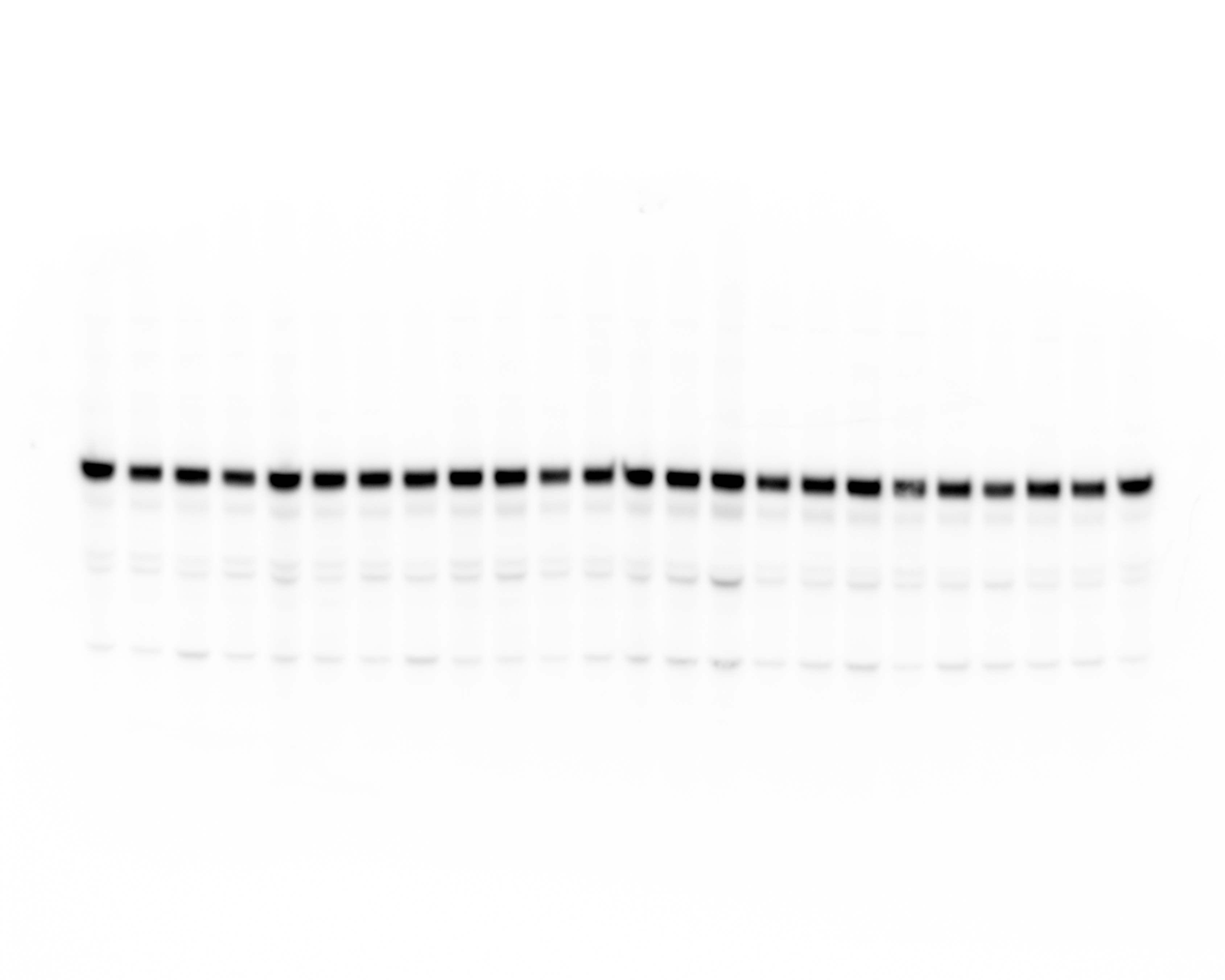

Supplement: Figure 5—figure supplement 1—source data 1. [file elife-90419-fig5-figsupp1-data1.zip › Figure 5-figure supplement 1_raw images/Fig5s1 HFFD M tAMPK.jpg]

Figure 5-figure supplement 1

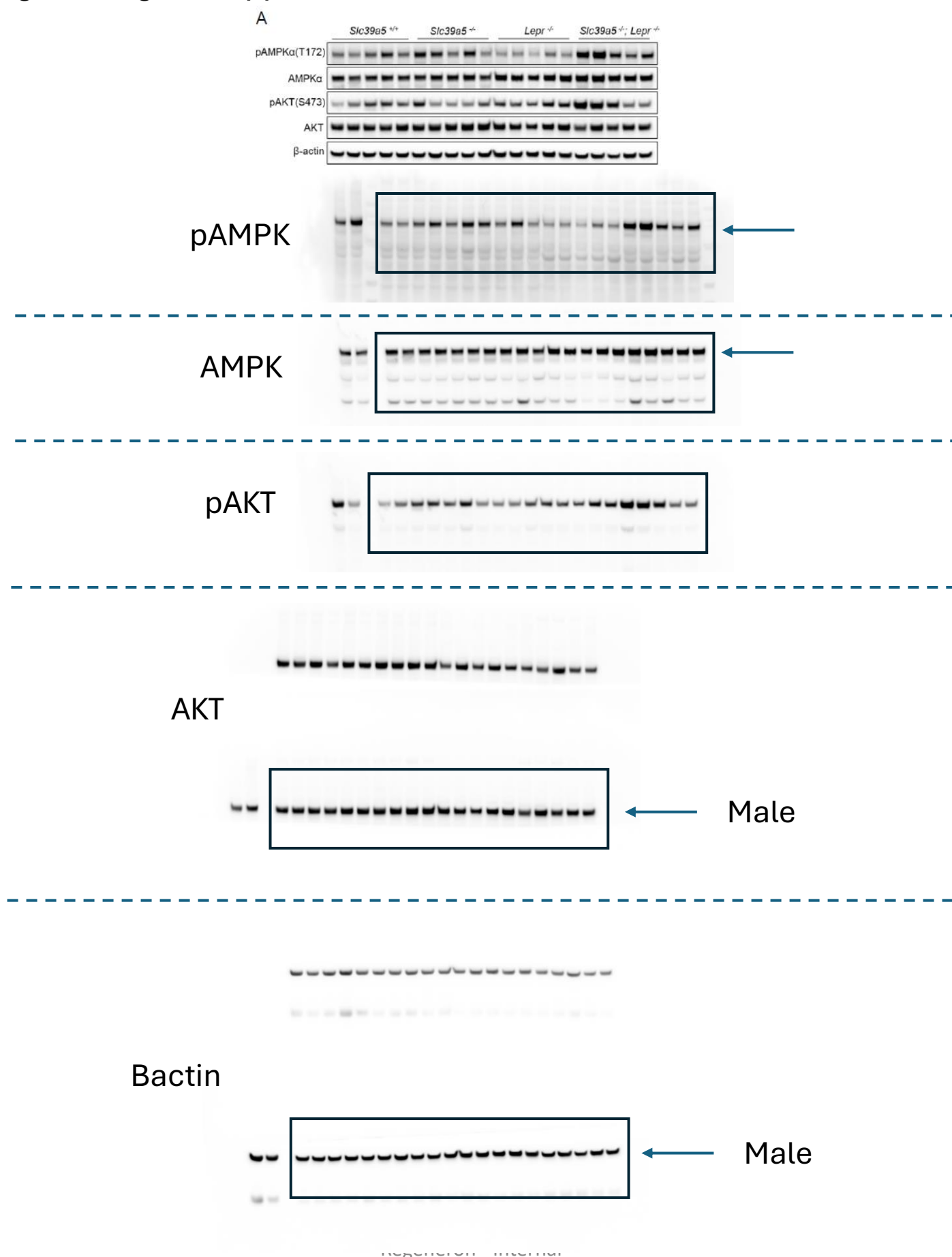

Supplementary Figure 10

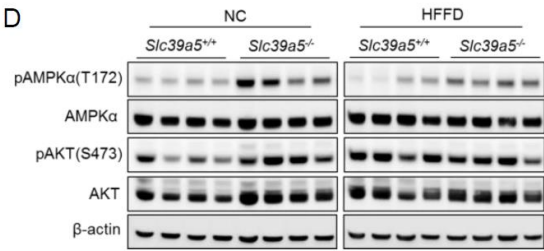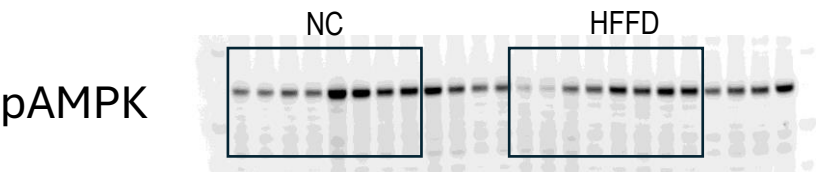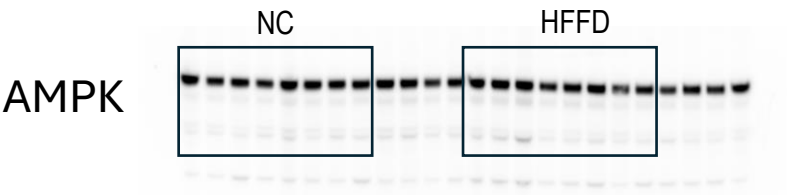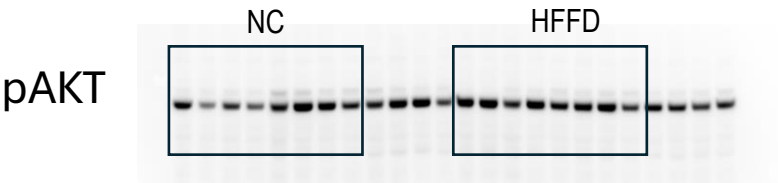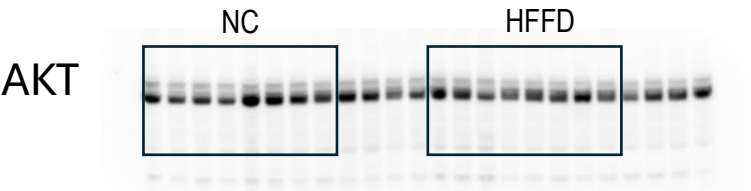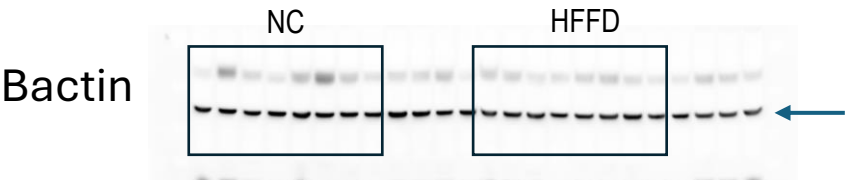

Supplement: Figure 5—figure supplement 1—source data 2. [file elife-90419-fig5-figsupp1-data2.zip › Figure 5-figure supplement 1_uncropped_labelled_images/Fig5s1_uncropped_labelled_images.pdf]

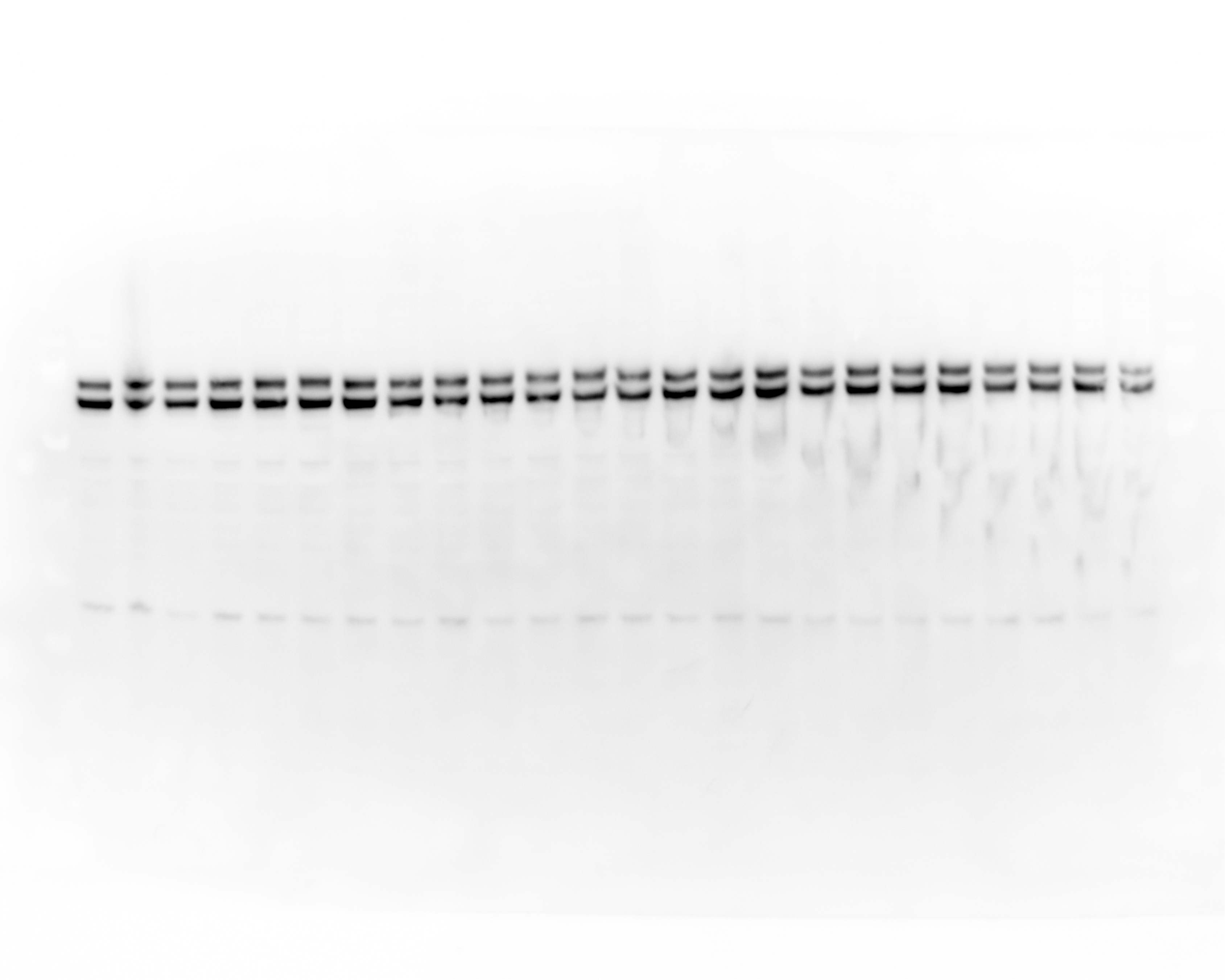

Supplement: Figure 5—figure supplement 3—source data 1. [file elife-90419-fig5-figsupp3-data1.zip › Figure 5-figure supplement 3_raw images/Fig5s3 tAKT.jpg]

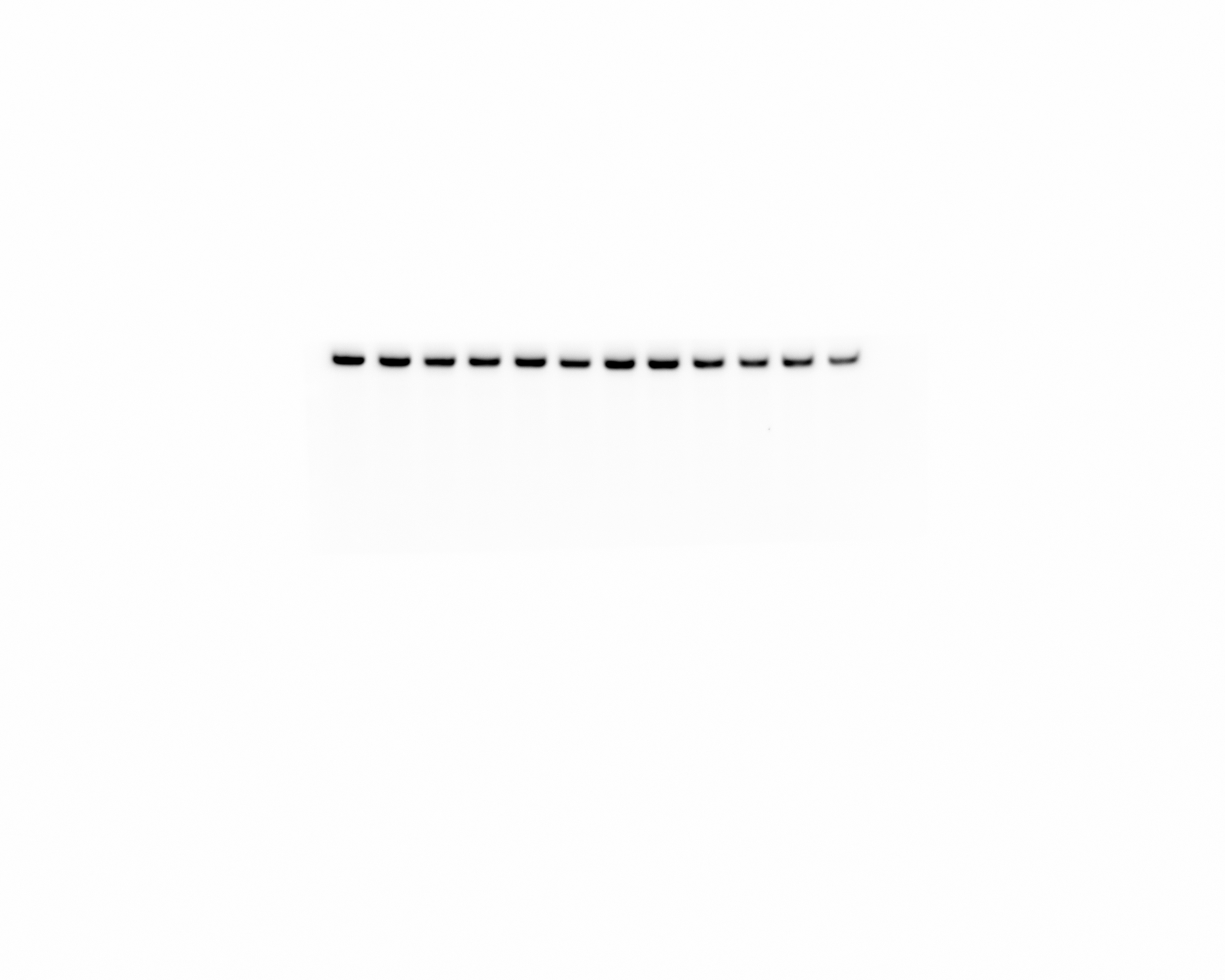

Supplement: Figure 5—figure supplement 3—source data 1. [file elife-90419-fig5-figsupp3-data1.zip › Figure 5-figure supplement 3_raw images/Fig5s3B Bactin.jpg]

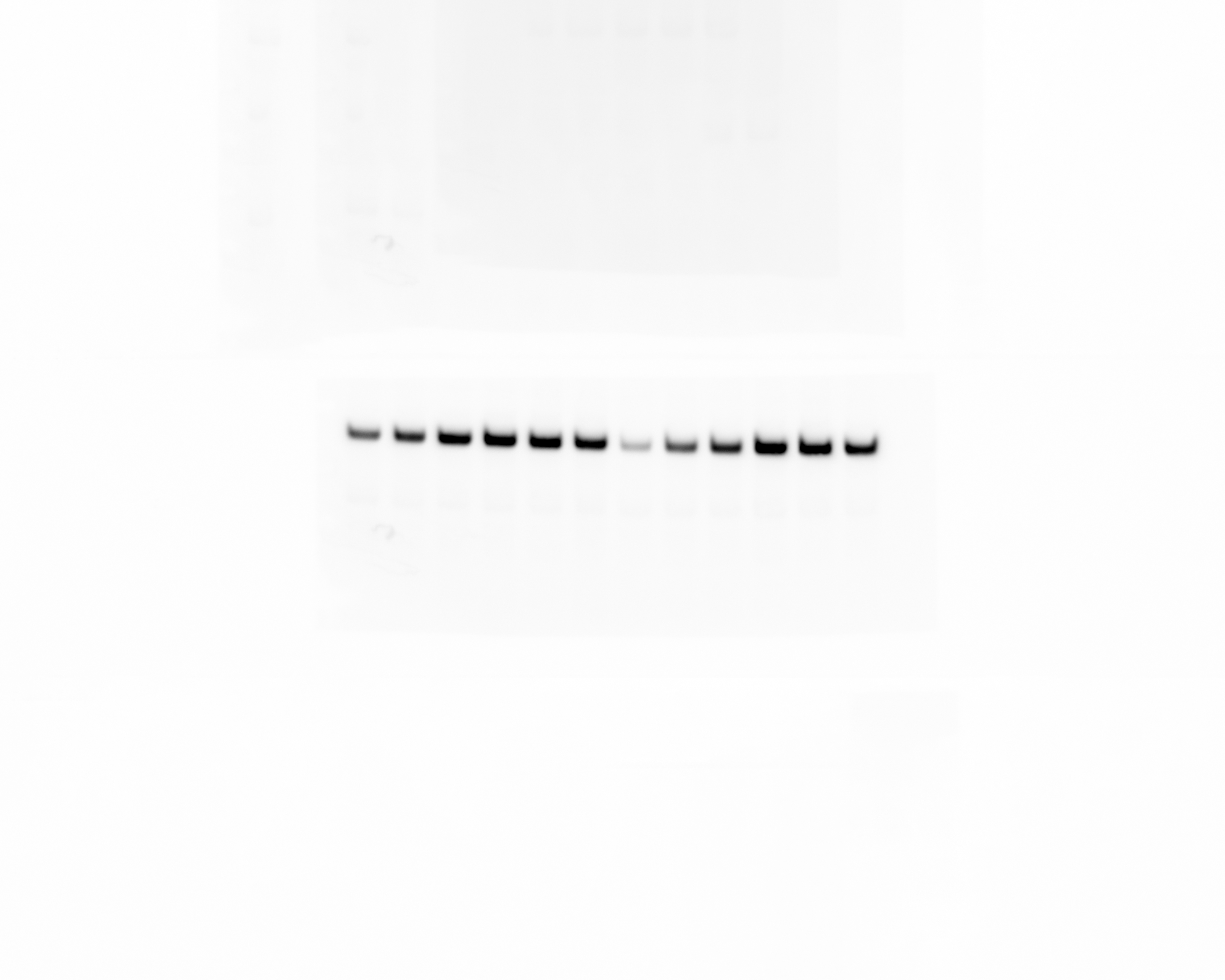

Supplement: Figure 5—figure supplement 3—source data 1. [file elife-90419-fig5-figsupp3-data1.zip › Figure 5-figure supplement 3_raw images/Fig5s3B pAKT.jpg]

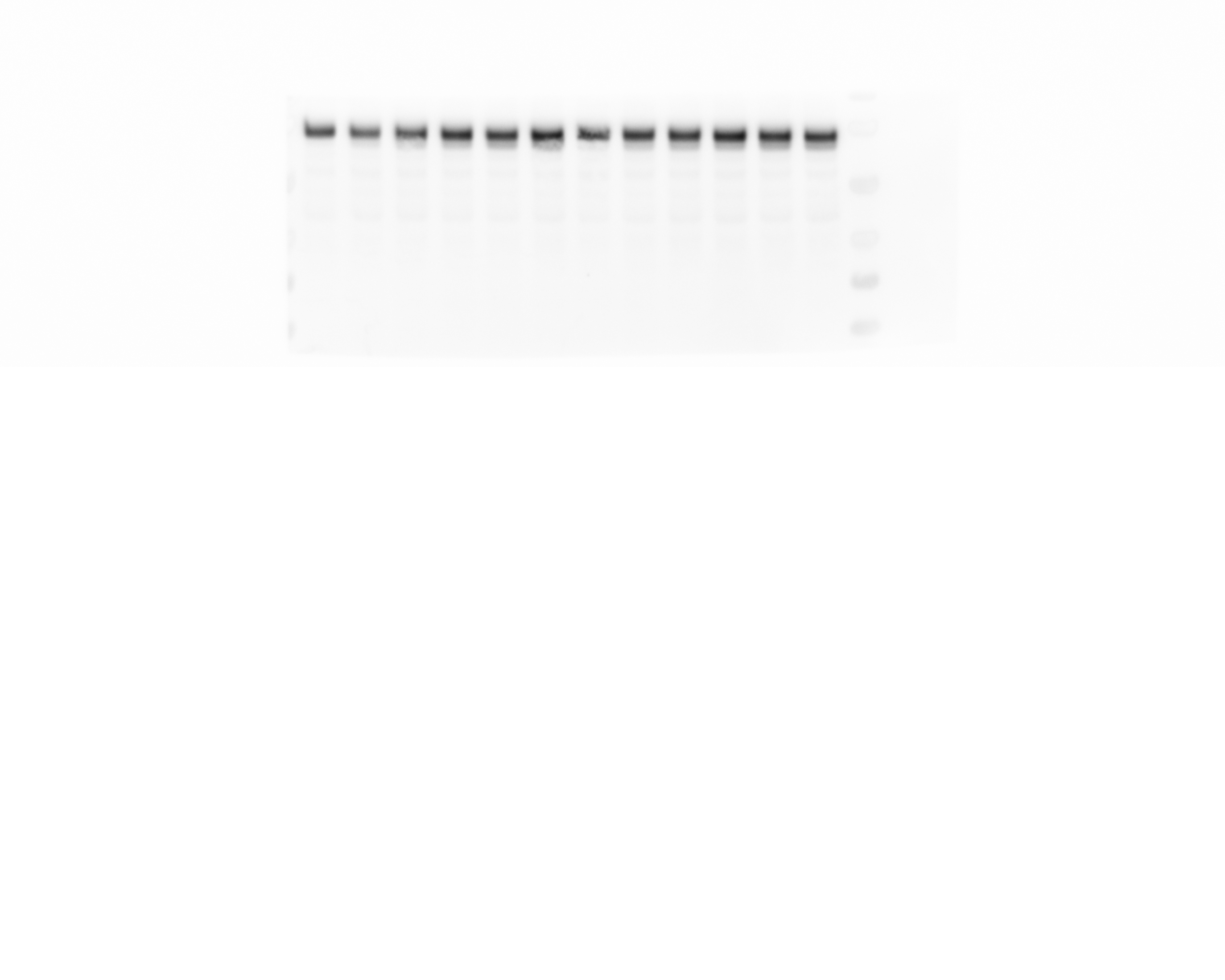

Supplement: Figure 5—figure supplement 3—source data 1. [file elife-90419-fig5-figsupp3-data1.zip › Figure 5-figure supplement 3_raw images/Fig5s3B pAMPK.jpg]

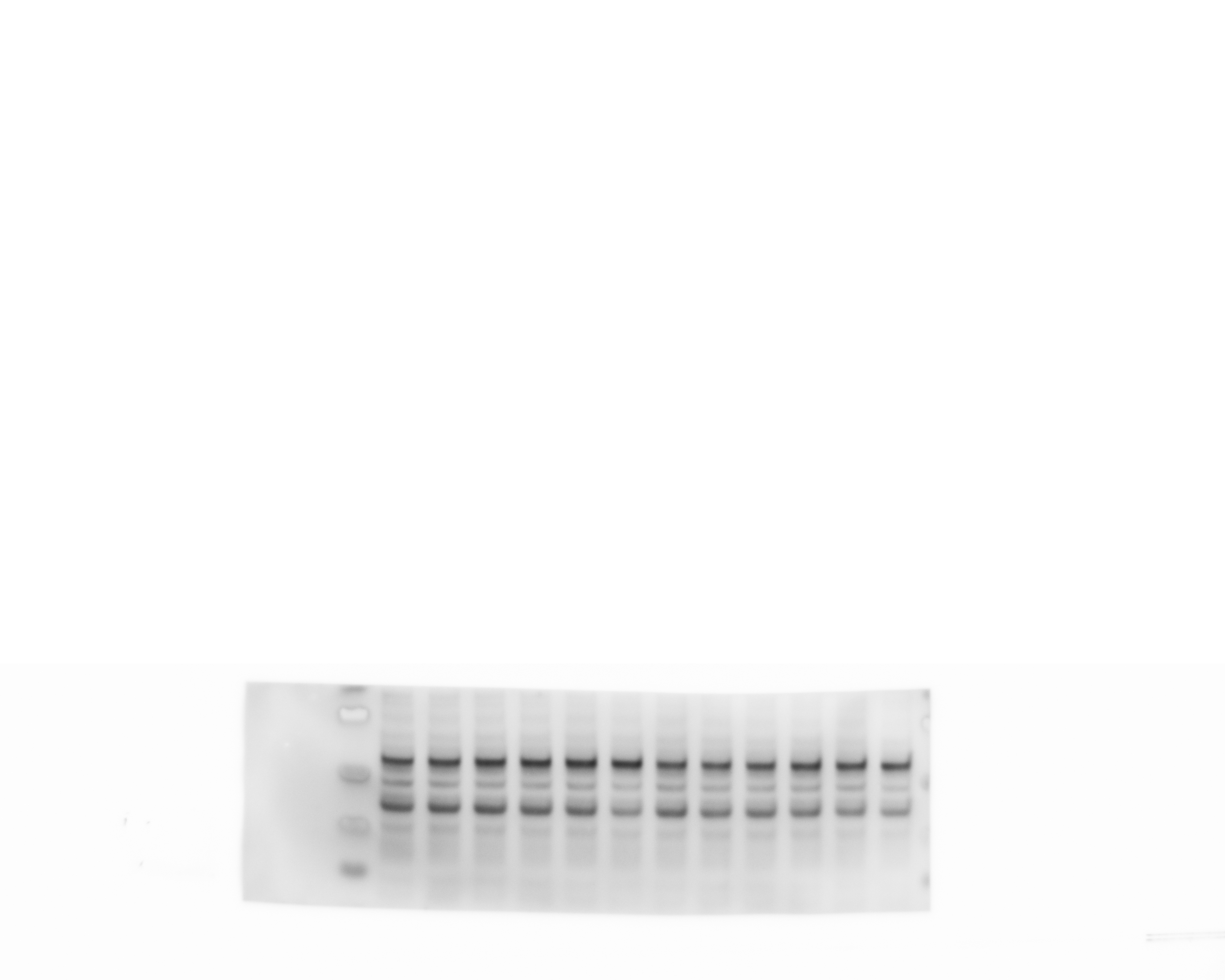

Supplement: Figure 5—figure supplement 3—source data 1. [file elife-90419-fig5-figsupp3-data1.zip › Figure 5-figure supplement 3_raw images/Fig5s3B pLKB1.jpg]

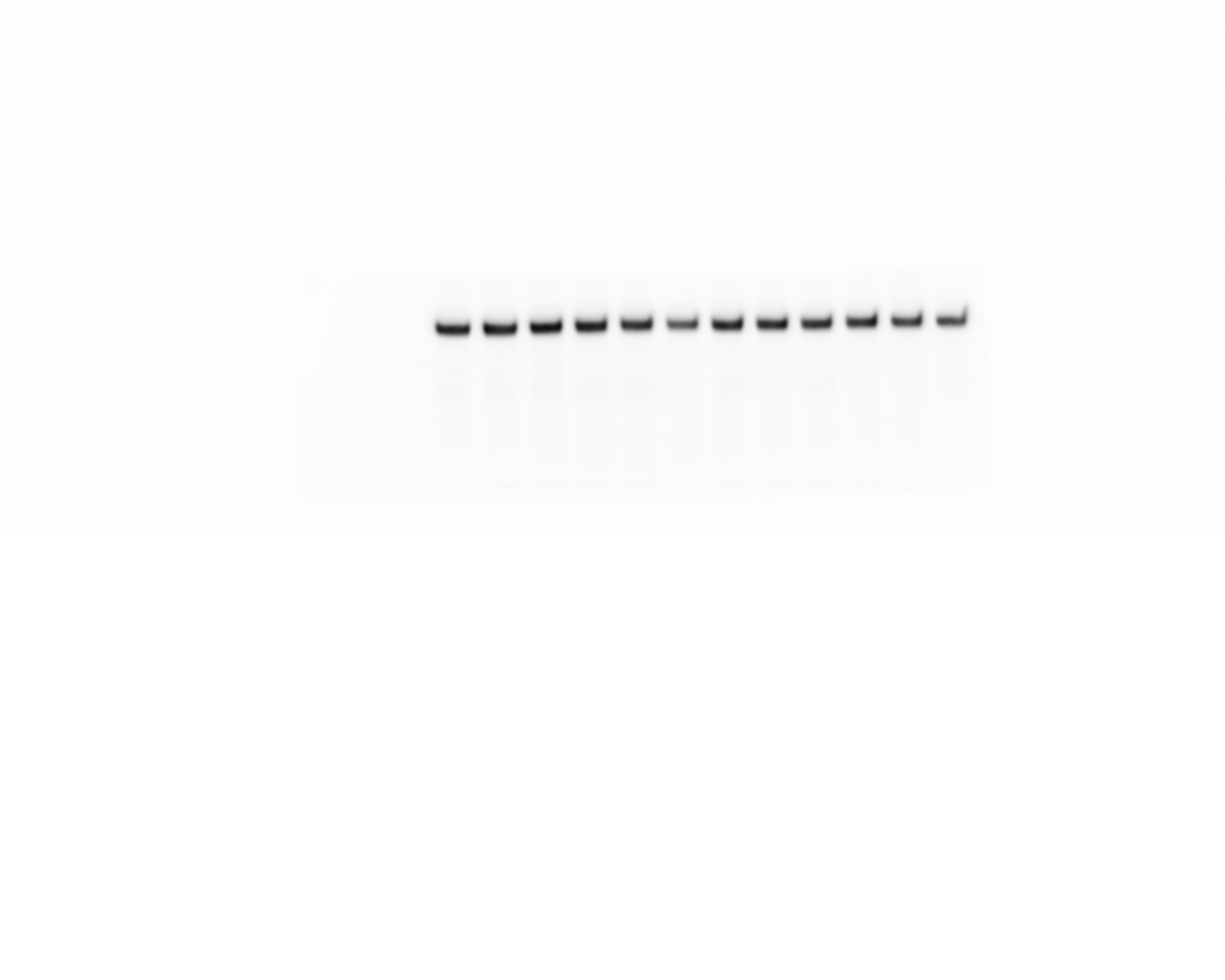

Supplement: Figure 5—figure supplement 3—source data 1. [file elife-90419-fig5-figsupp3-data1.zip › Figure 5-figure supplement 3_raw images/Fig5s3B tAKT.jpg]

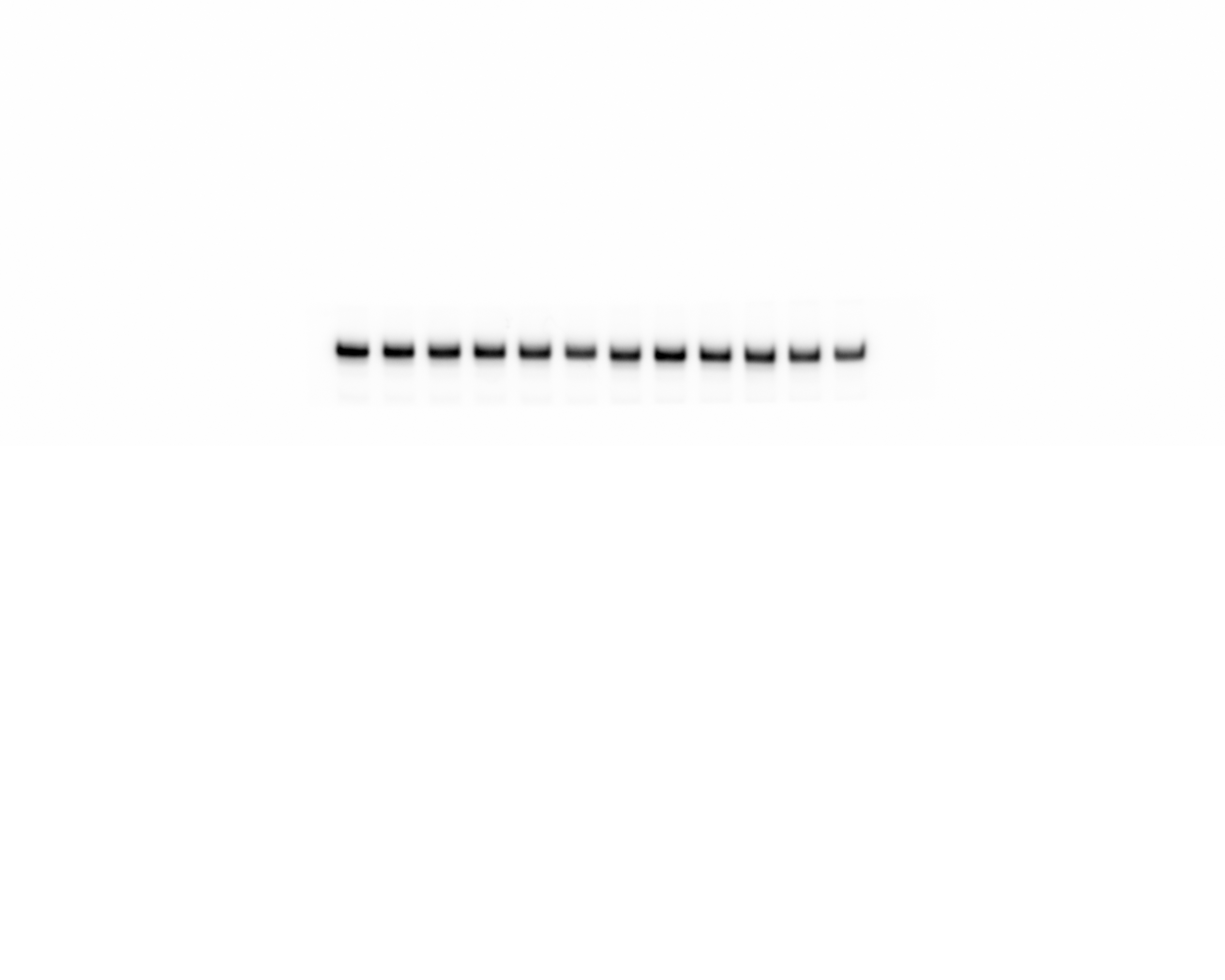

Supplement: Figure 5—figure supplement 3—source data 1. [file elife-90419-fig5-figsupp3-data1.zip › Figure 5-figure supplement 3_raw images/Fig5s3B tAMPK.jpg]

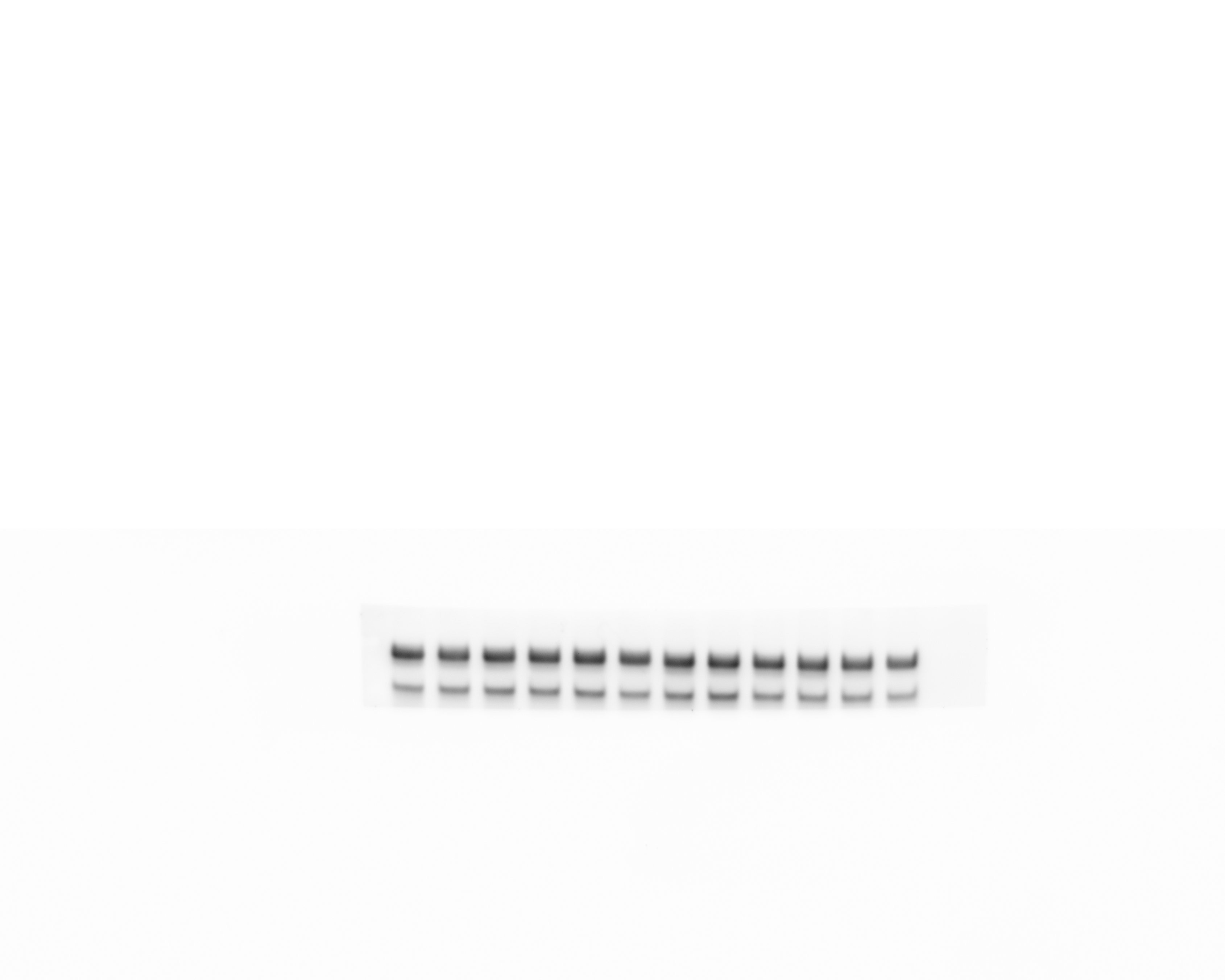

Supplement: Figure 5—figure supplement 3—source data 1. [file elife-90419-fig5-figsupp3-data1.zip › Figure 5-figure supplement 3_raw images/Fig5s3B tLKB1.jpg]

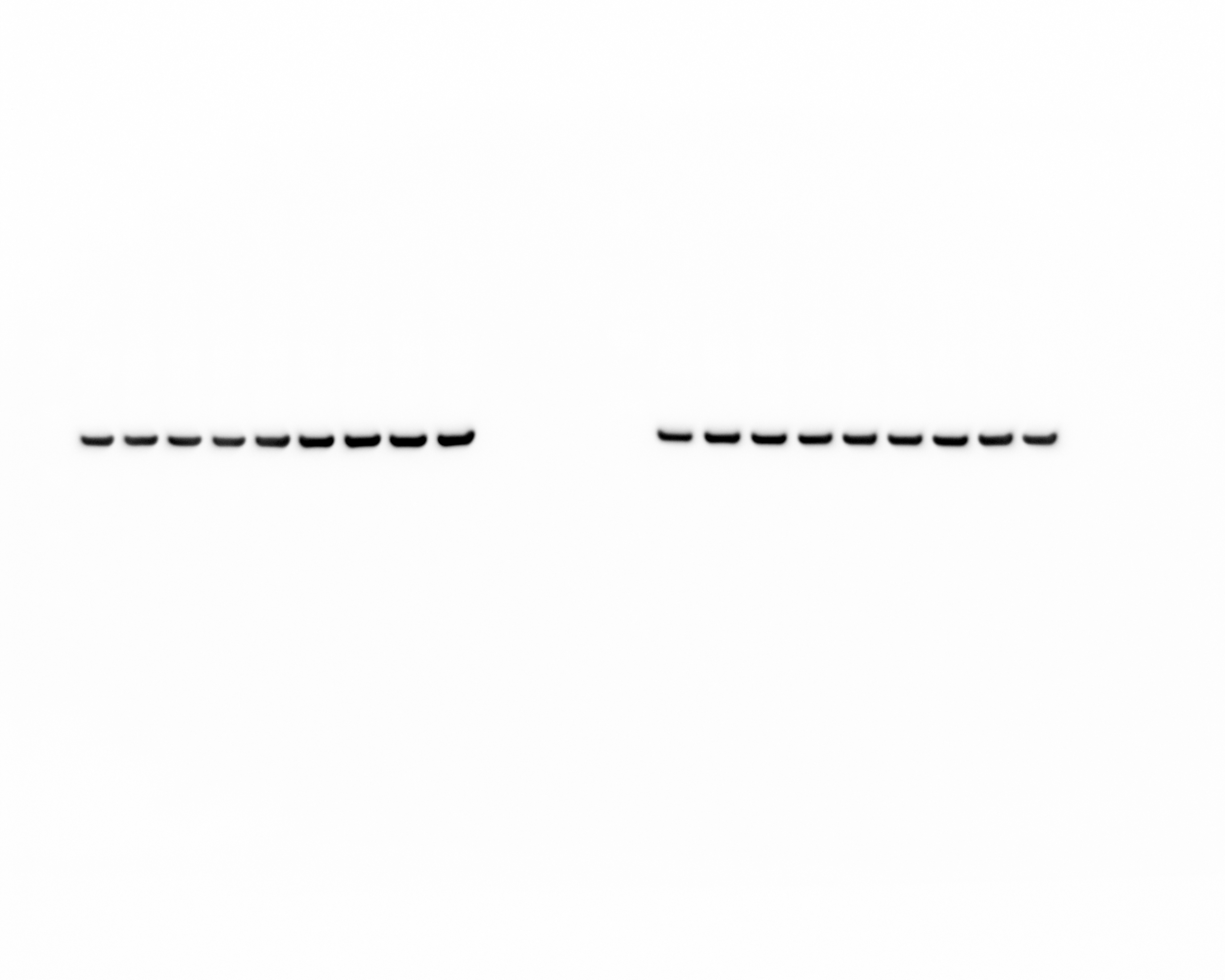

Supplement: Figure 5—figure supplement 3—source data 1. [file elife-90419-fig5-figsupp3-data1.zip › Figure 5-figure supplement 3_raw images/Fig5s3C Bactin.jpg]

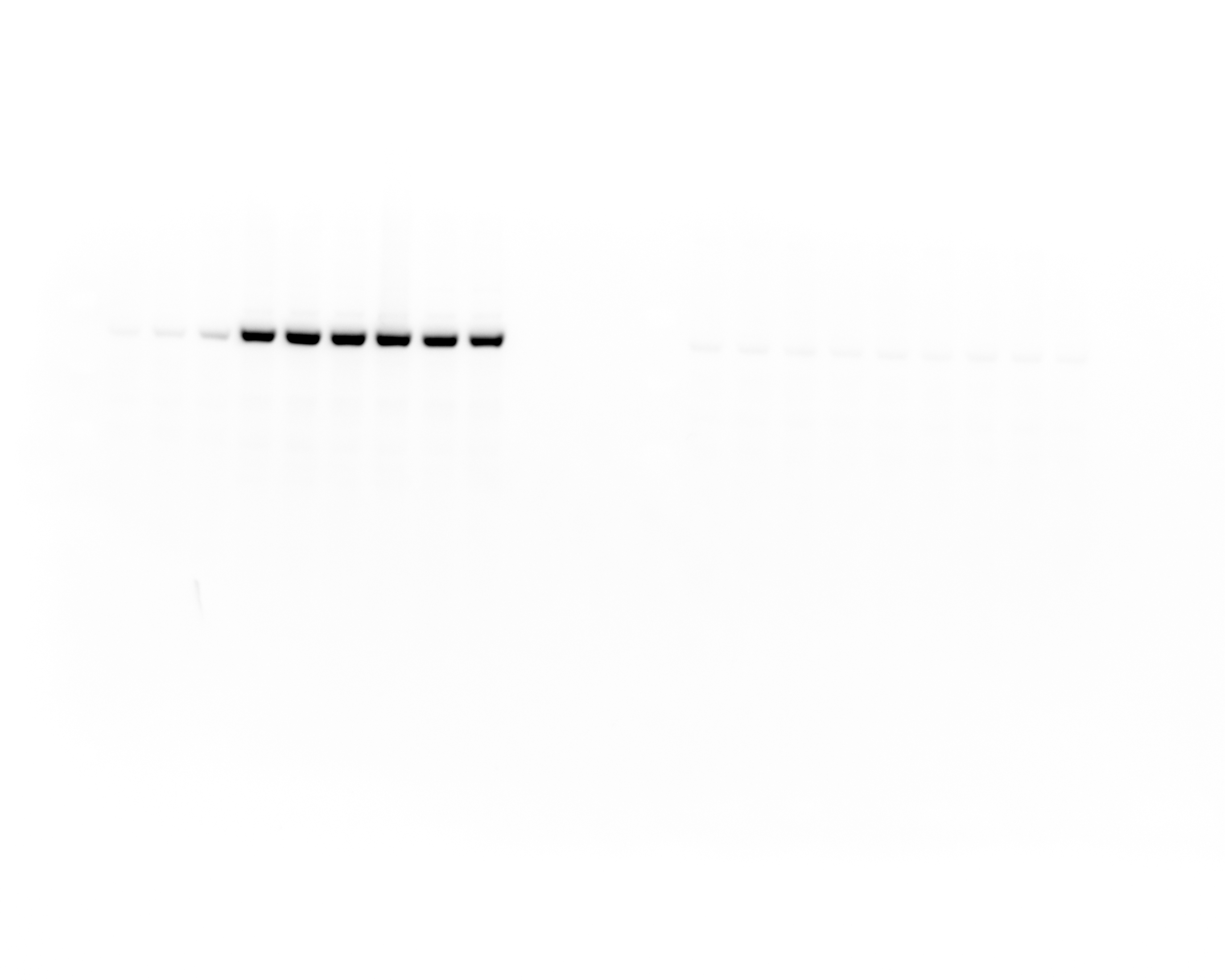

Supplement: Figure 5—figure supplement 3—source data 1. [file elife-90419-fig5-figsupp3-data1.zip › Figure 5-figure supplement 3_raw images/Fig5s3C pAKT.jpg]

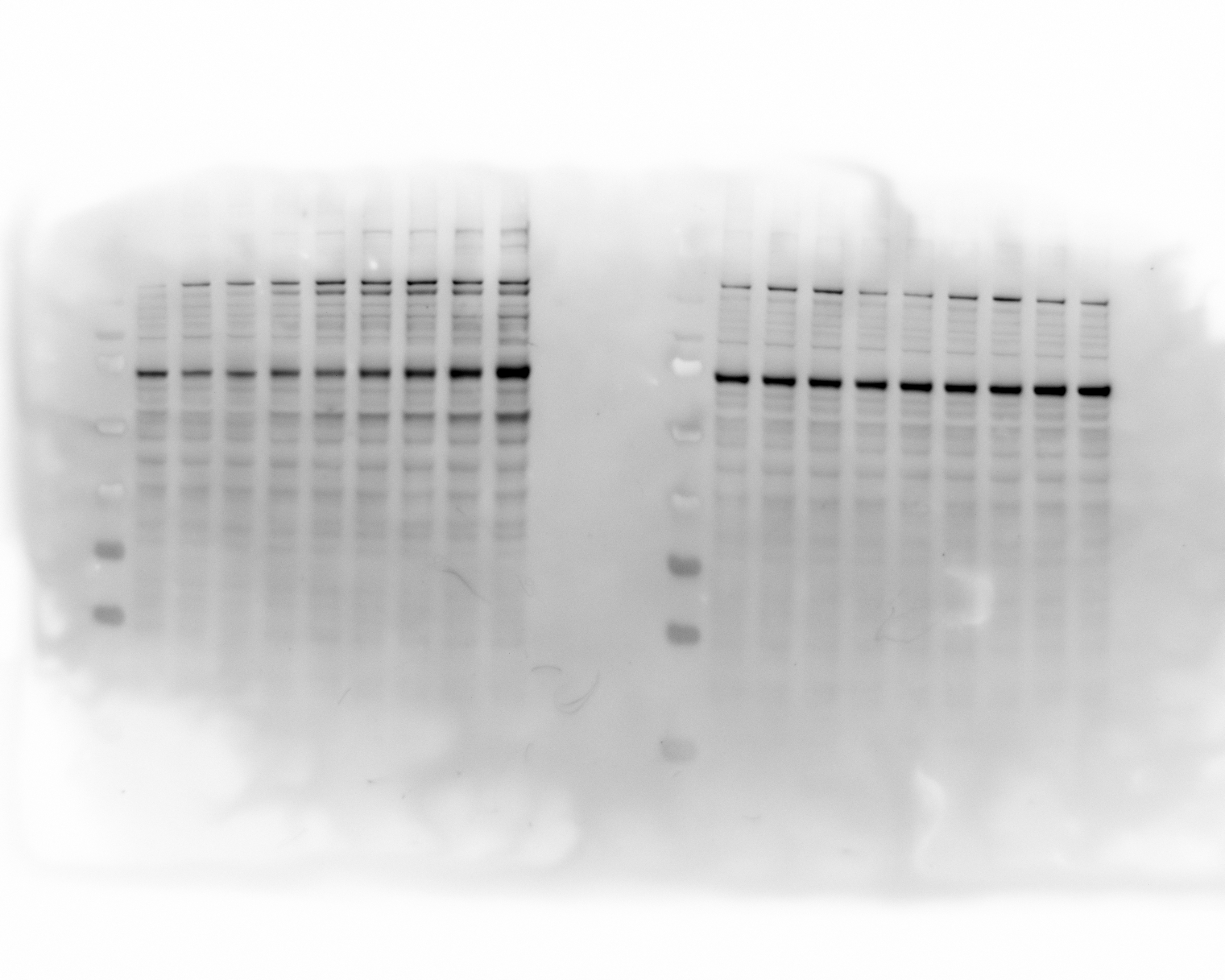

Supplement: Figure 5—figure supplement 3—source data 1. [file elife-90419-fig5-figsupp3-data1.zip › Figure 5-figure supplement 3_raw images/Fig5s3C pAMPK.jpg]

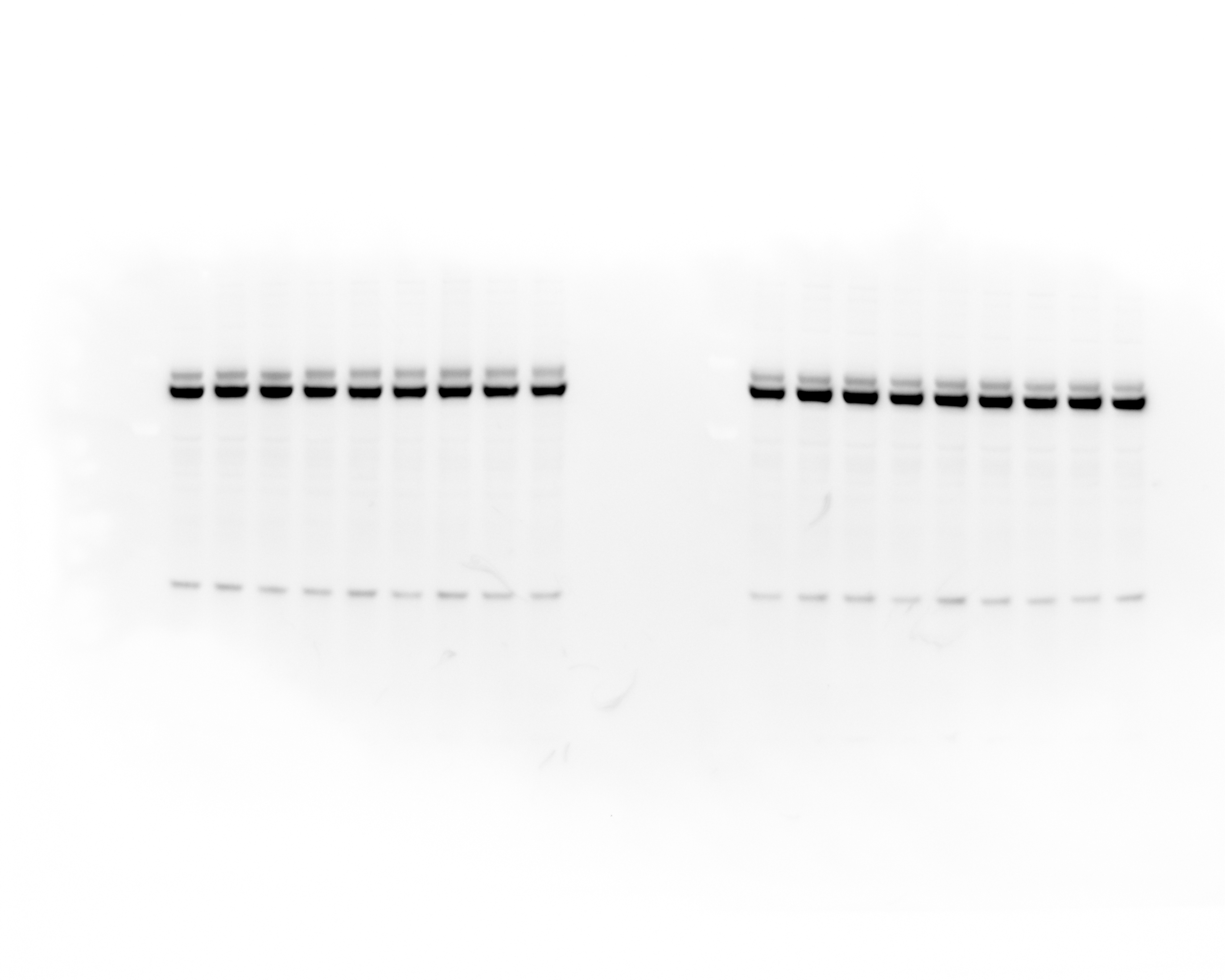

Supplement: Figure 5—figure supplement 3—source data 1. [file elife-90419-fig5-figsupp3-data1.zip › Figure 5-figure supplement 3_raw images/Fig5s3C tAKT.jpg]

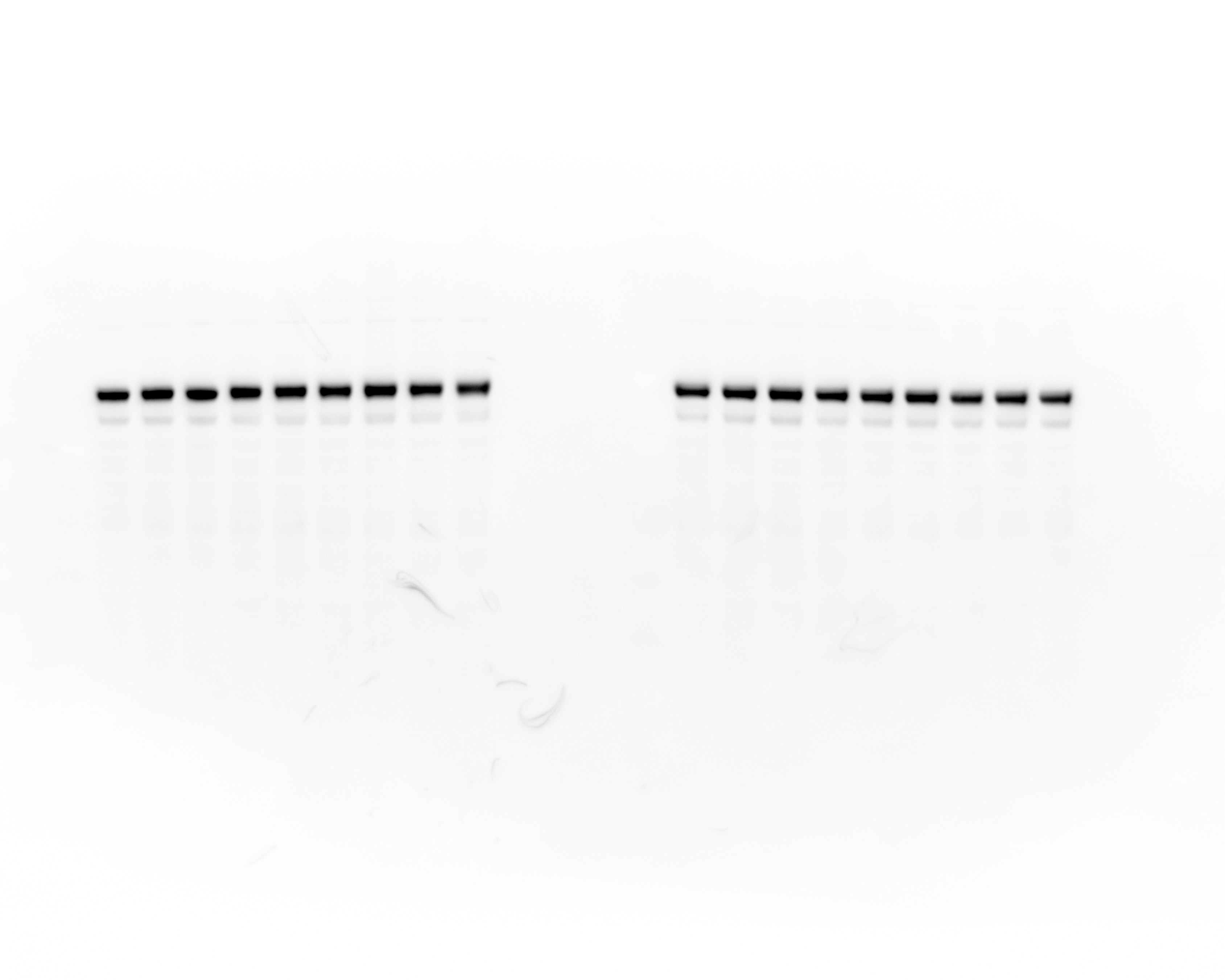

Supplement: Figure 5—figure supplement 3—source data 1. [file elife-90419-fig5-figsupp3-data1.zip › Figure 5-figure supplement 3_raw images/Fig5s3C tAMPK.jpg]

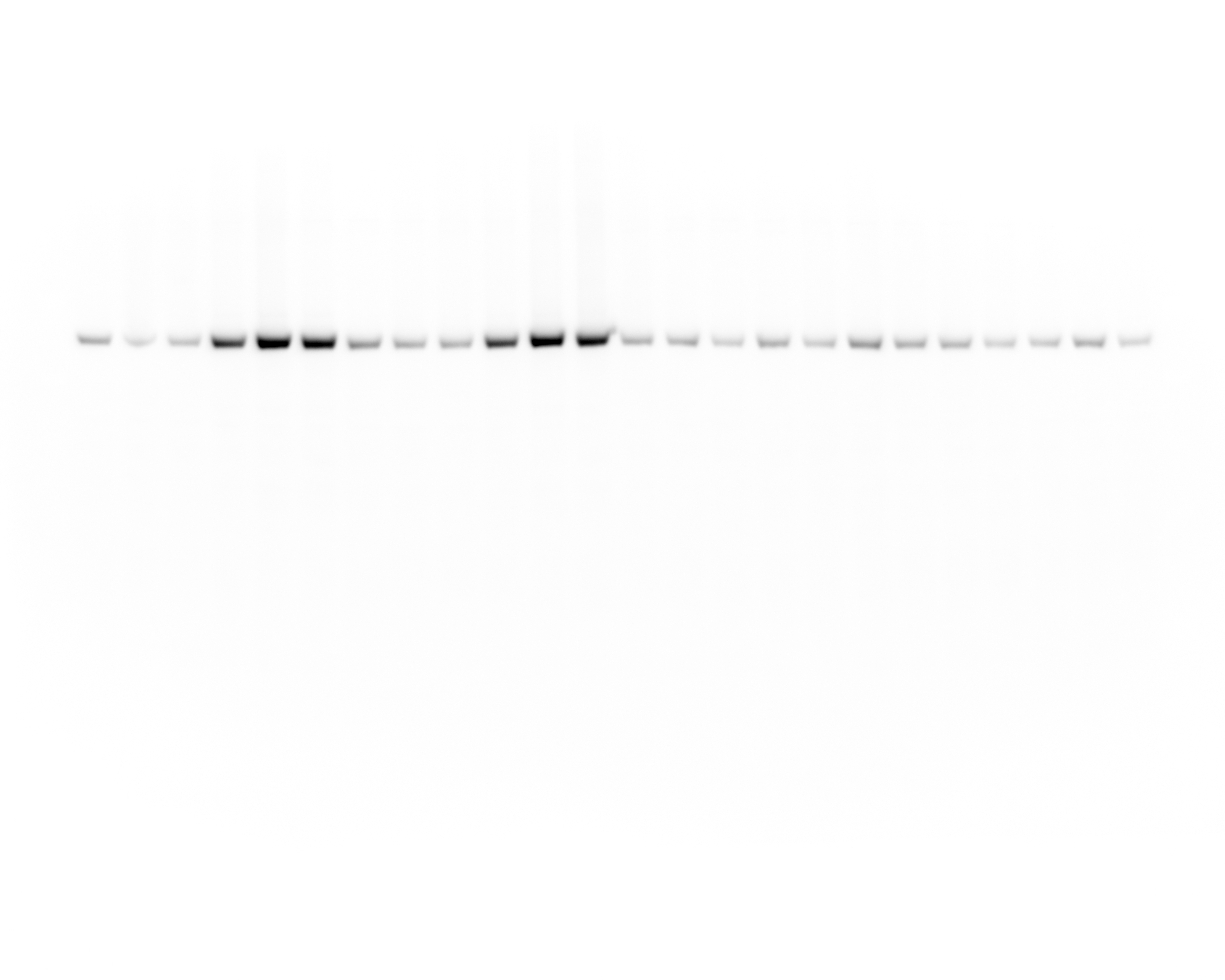

Supplement: Figure 5—figure supplement 3—source data 1. [file elife-90419-fig5-figsupp3-data1.zip › Figure 5-figure supplement 3_raw images/Fig5s3D pAKT.jpg]

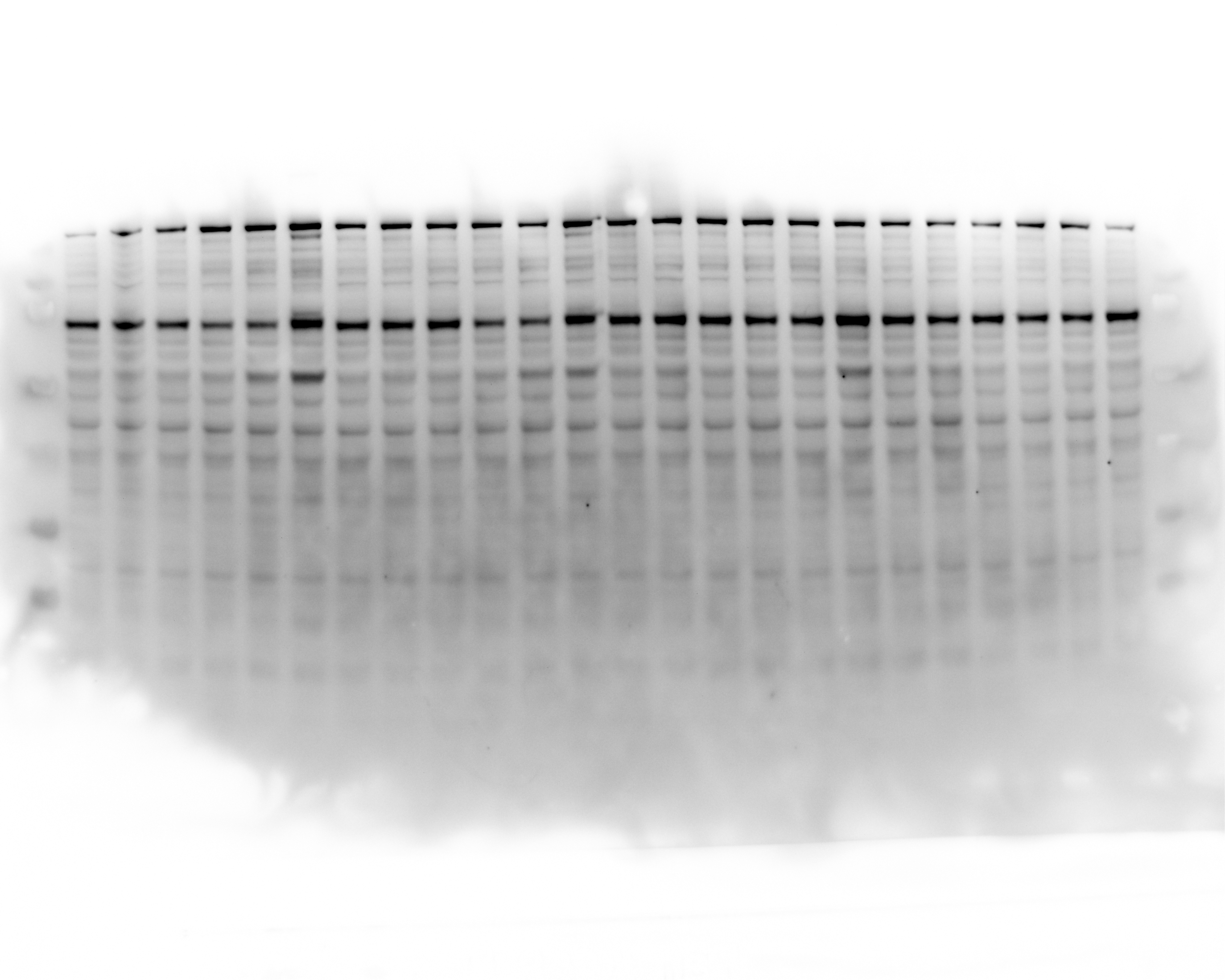

Supplement: Figure 5—figure supplement 3—source data 1. [file elife-90419-fig5-figsupp3-data1.zip › Figure 5-figure supplement 3_raw images/Fig5s3D pAMPK.jpg]

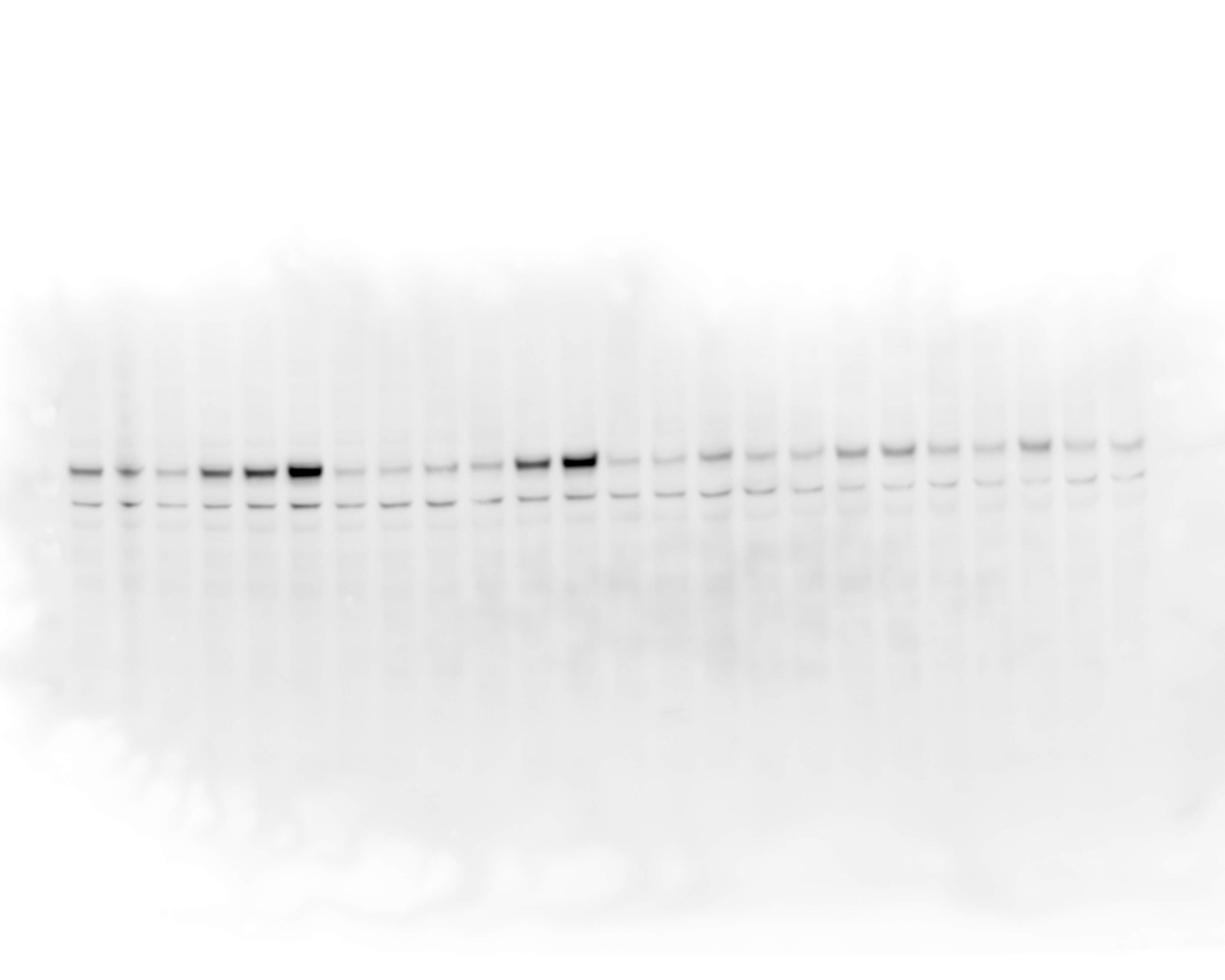

Supplement: Figure 5—figure supplement 3—source data 1. [file elife-90419-fig5-figsupp3-data1.zip › Figure 5-figure supplement 3_raw images/Fig5s3D pLKB1.jpg]

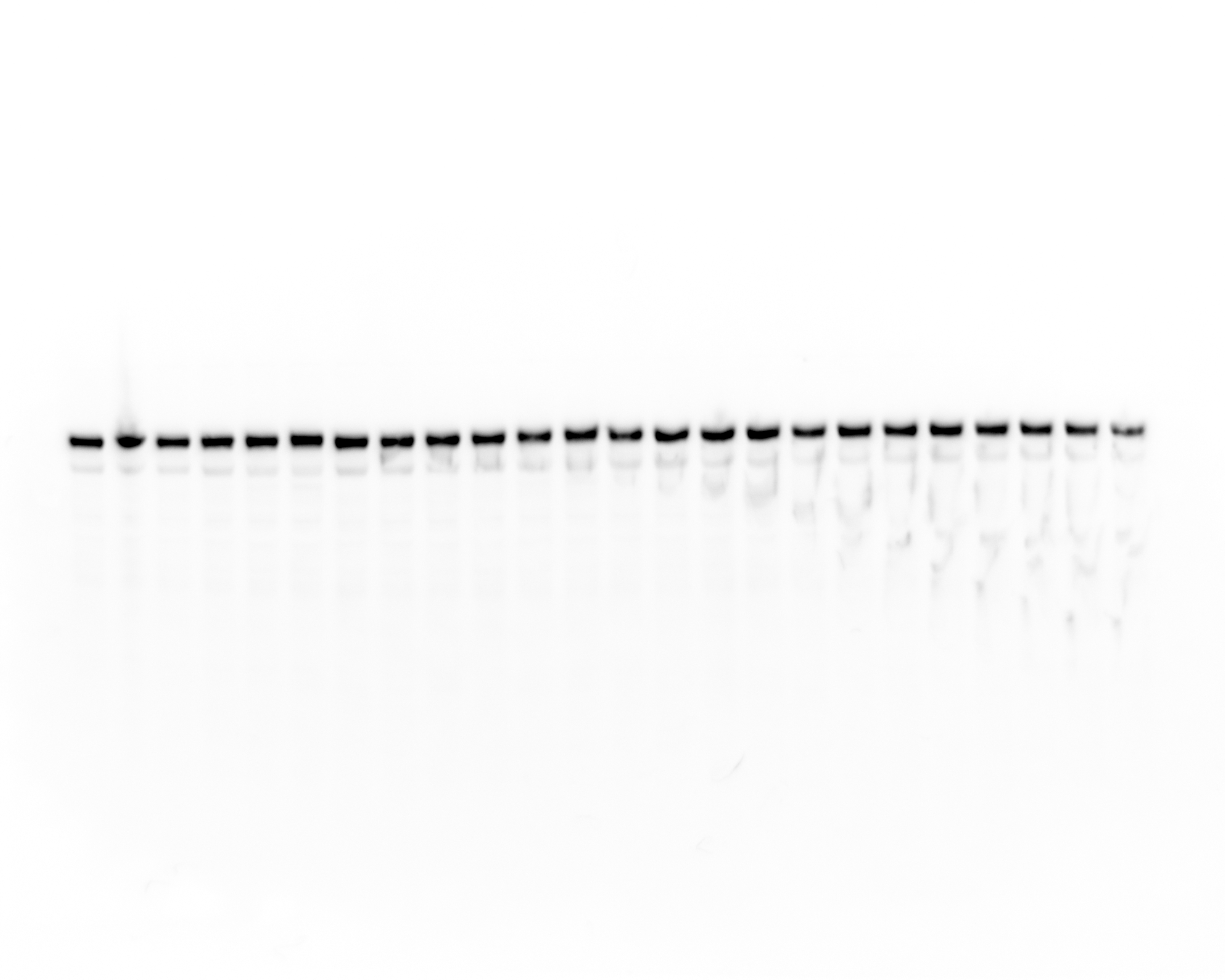

Supplement: Figure 5—figure supplement 3—source data 1. [file elife-90419-fig5-figsupp3-data1.zip › Figure 5-figure supplement 3_raw images/Fig5s3D tAMPK.jpg]

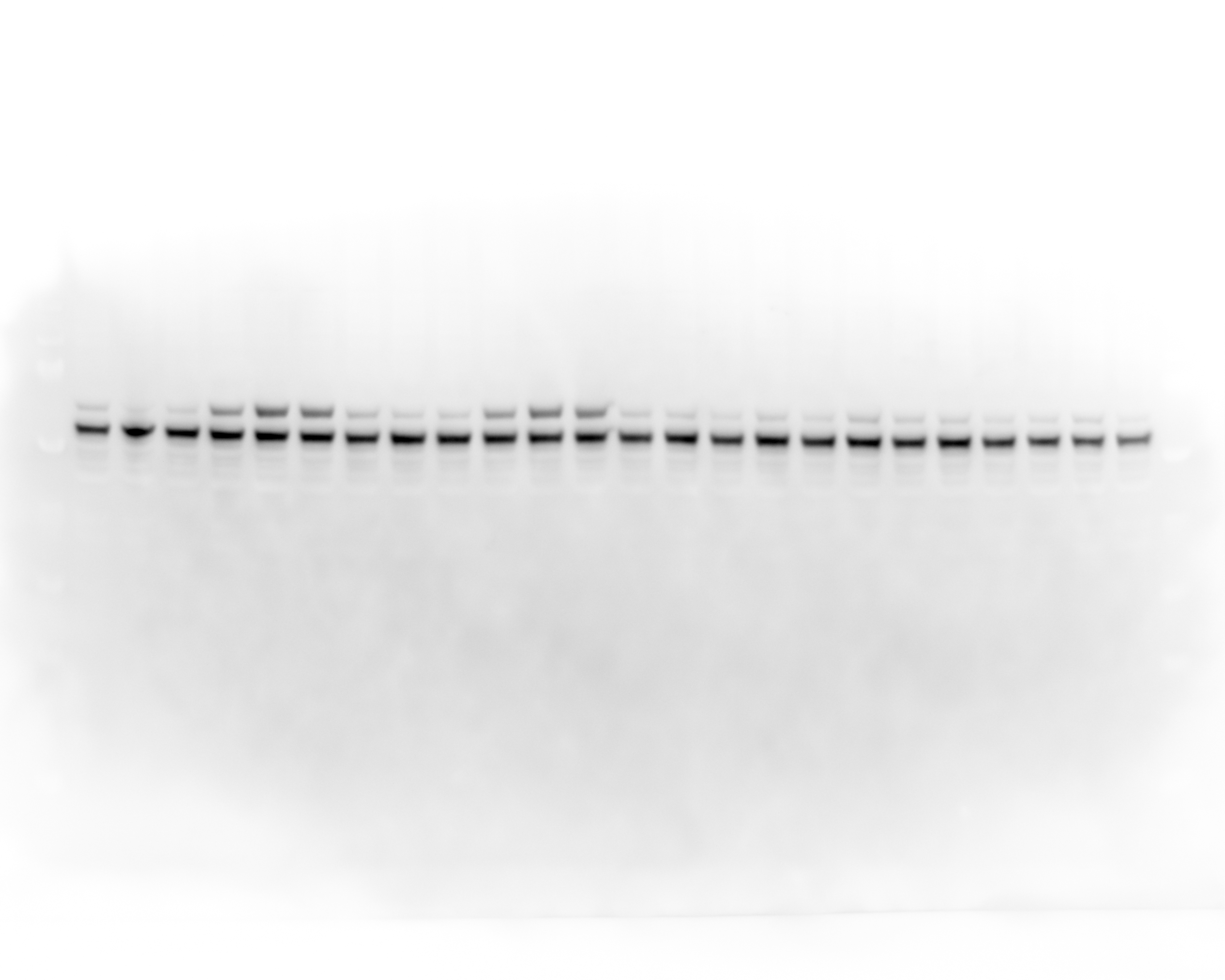

Supplement: Figure 5—figure supplement 3—source data 1. [file elife-90419-fig5-figsupp3-data1.zip › Figure 5-figure supplement 3_raw images/Fig5s3D tLKB1.jpg]

B

B

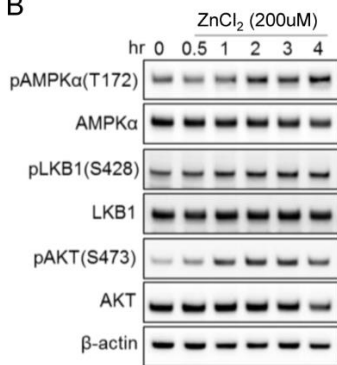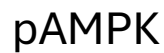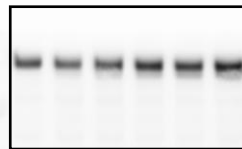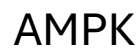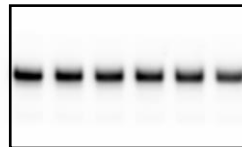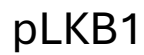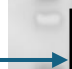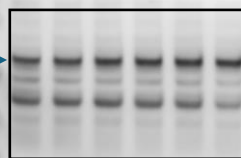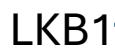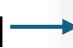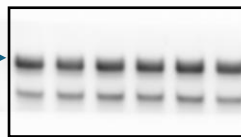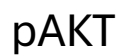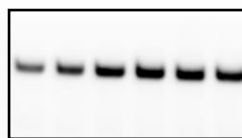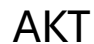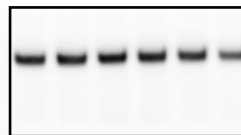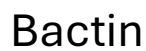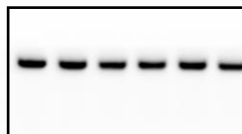

# Supplementary Figure 13

C

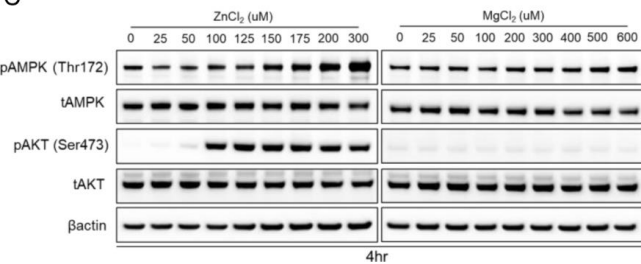

pAMPK

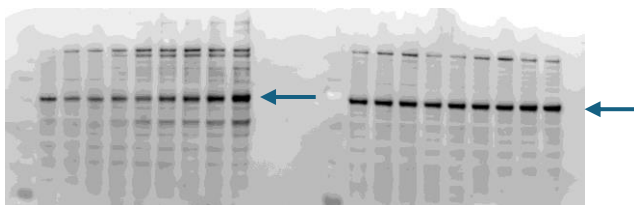

AMPK

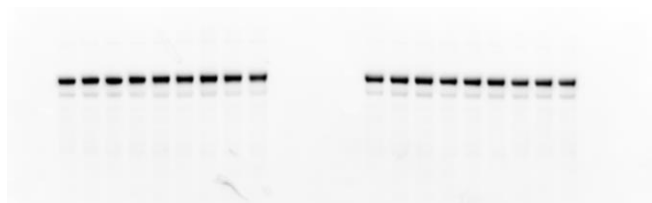

pAKT

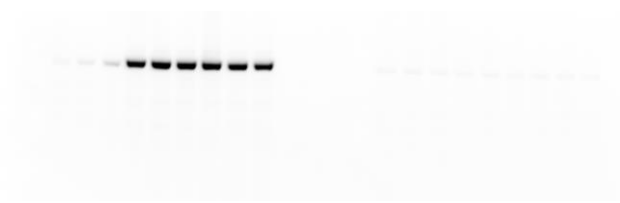

AKT

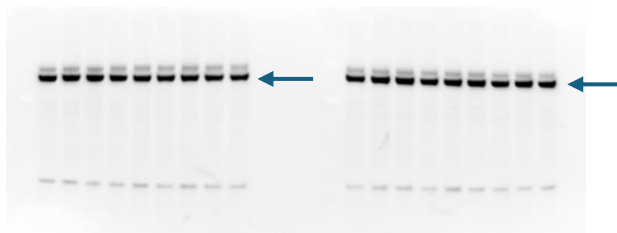

Bactin

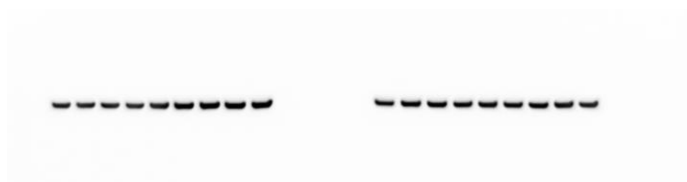

# Supplementary Figure 13

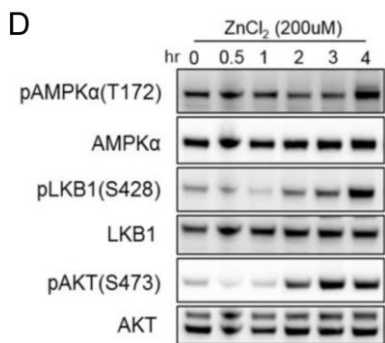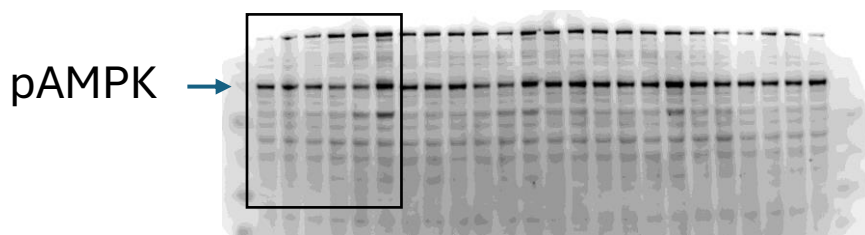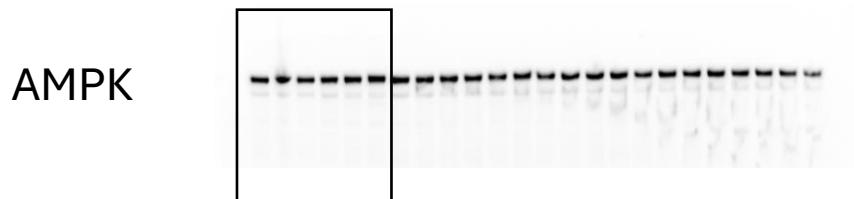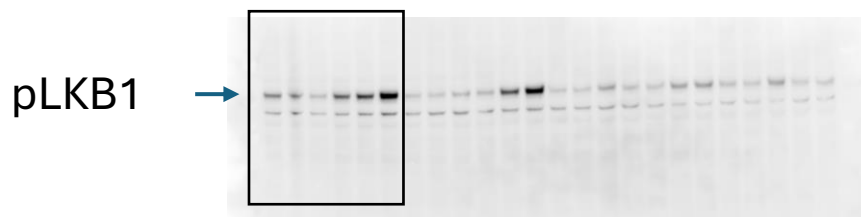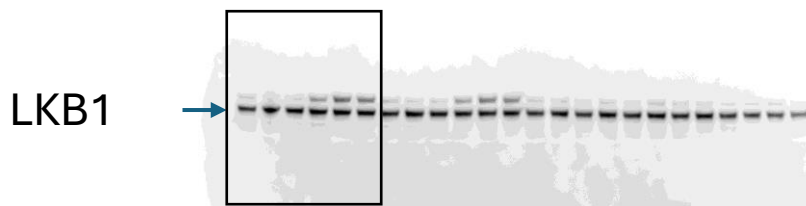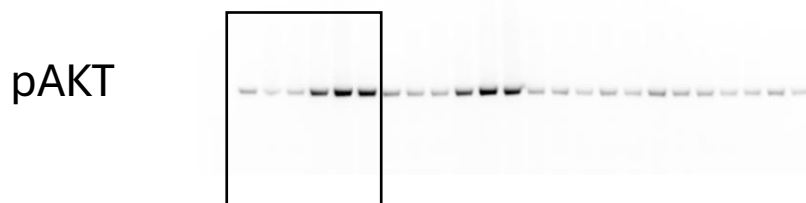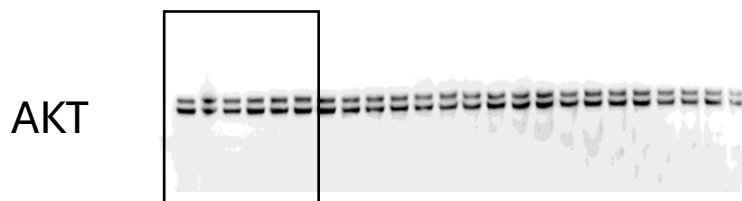

Supplement: Figure 5—figure supplement 3—source data 2. [file elife-90419-fig5-figsupp3-data2.zip › Figure 5-figure supplement 3_uncropped_labelled images/Fig5s3_uncropped_labelled_images.pdf]
