# Supplementary material for: Genetic inactivation of zinc transporter SLC39A5 improves liver function and hyperglycemia in obesogenic settings
Source: eLife. 2024 Dec 13;12:RP90419. doi: 10.7554/eLife.90419 (PMC11648992; doi:10.7554/eLife.90419)
Supplement: Figure 5—source data 2. [file elife-90419-fig5-data2.zip › Fig5_uncropped_labelled_images/Fig5_uncropped_labelled_images.pdf]

Figure 5

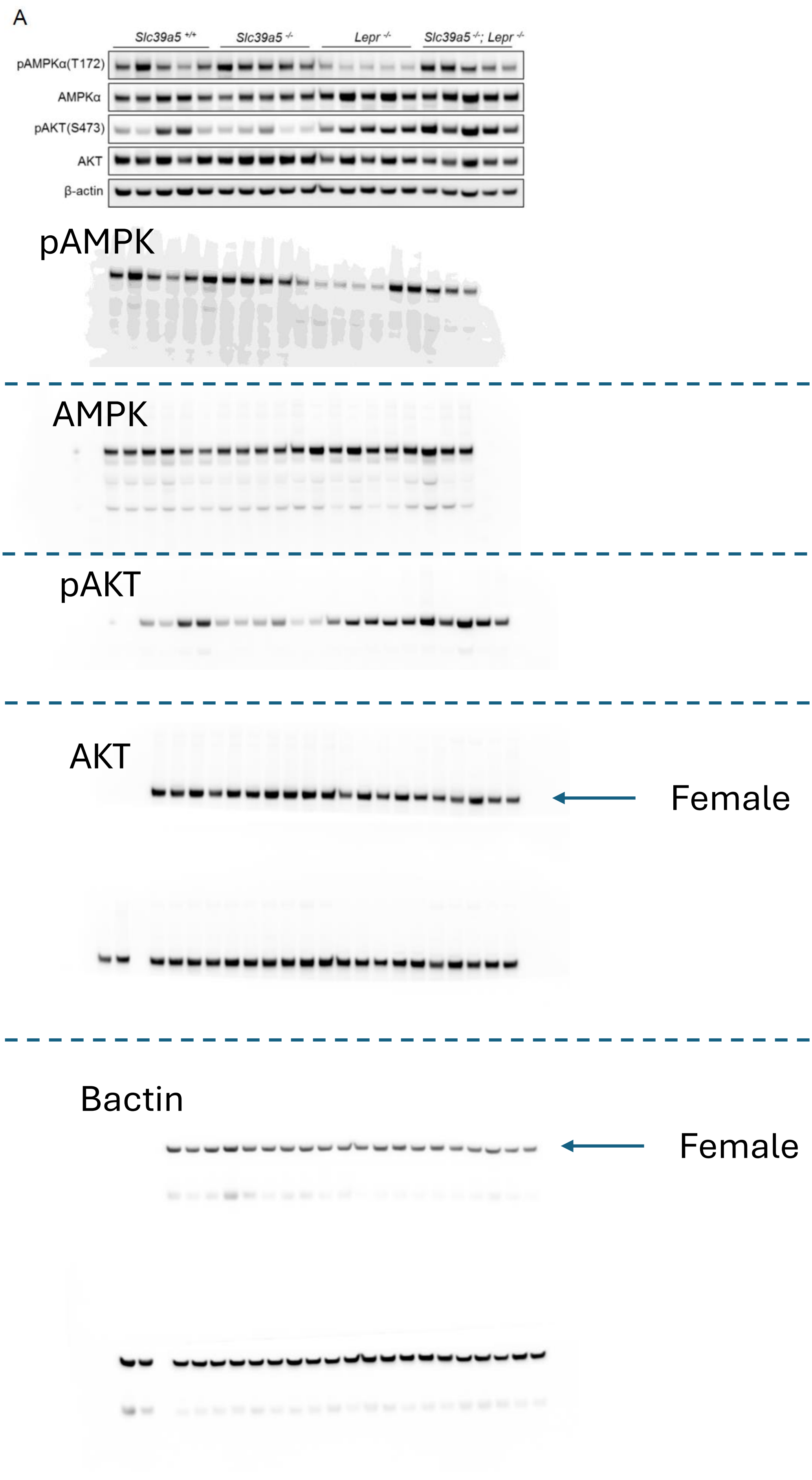
A
Slc39a5 +/+
Slc39a5 -/-
Lepr -/-
Slc39a5 -/-; Lepr -/-
pAMPKα(T172)
AMPKα
pAKT(S473)
AKT
β-actin
pAMPK
AMPK
pAKT
AKT
Female
Bactin
Female

Figure 5

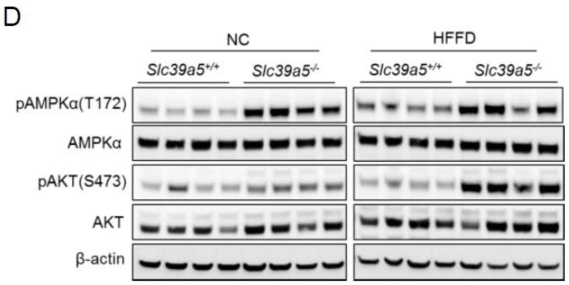
D
NC
HFFD
Slc39a5+/+
Slc39a5-/-
Slc39a5+/+
Slc39a5-/-
pAMPKα(T172)
AMPKα
pAKT(S473)
AKT
β-actin

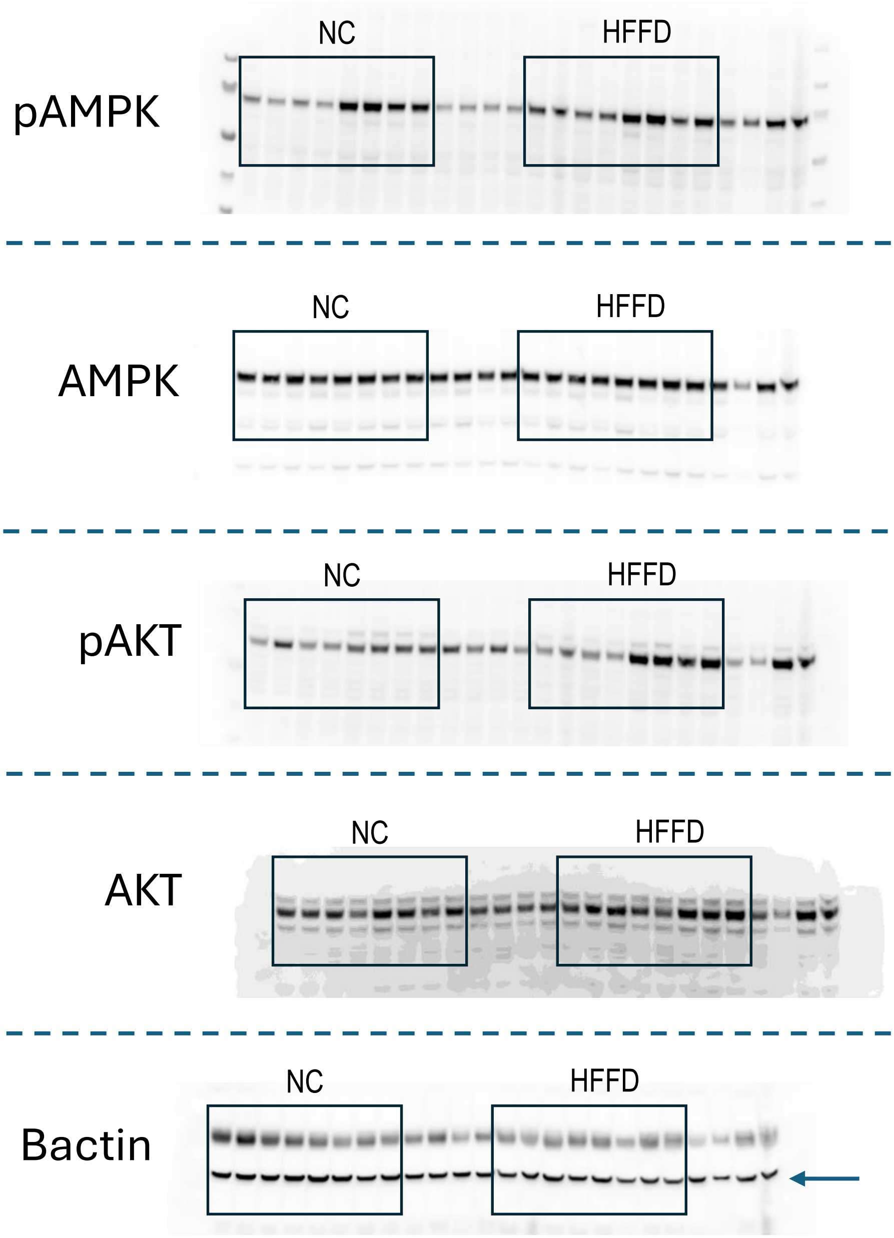
pAMPK
NC
HFFD
AMPK
NC
HFFD
pAKT
NC
HFFD
AKT
NC
HFFD
Bactin
NC
HFFD

Figure 5

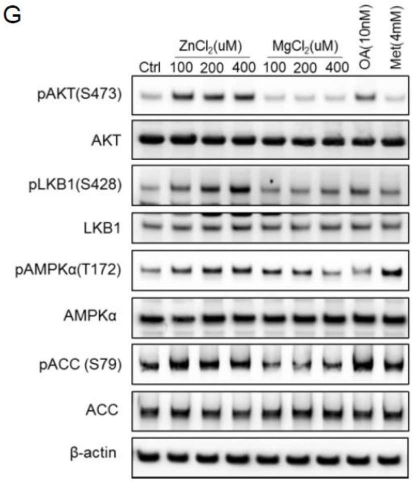

pAKT

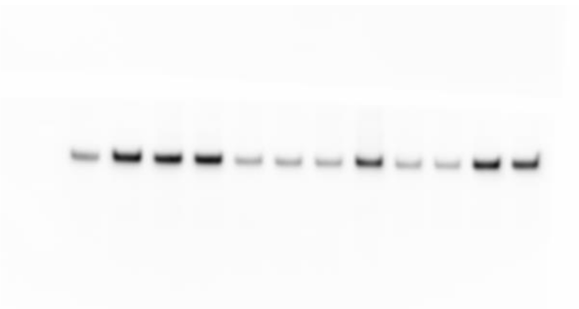

AKT

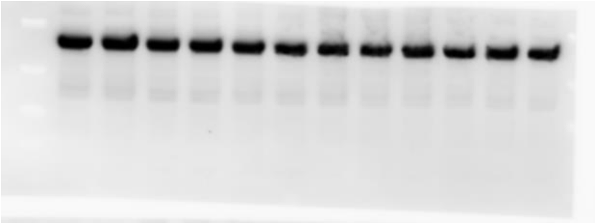

pLKB1

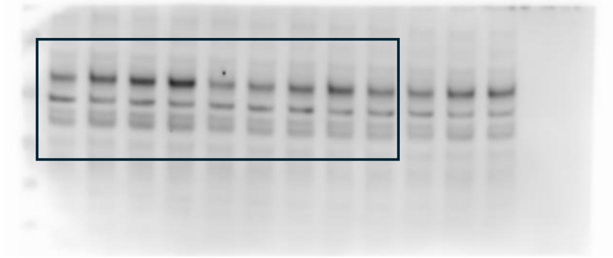

LKB1

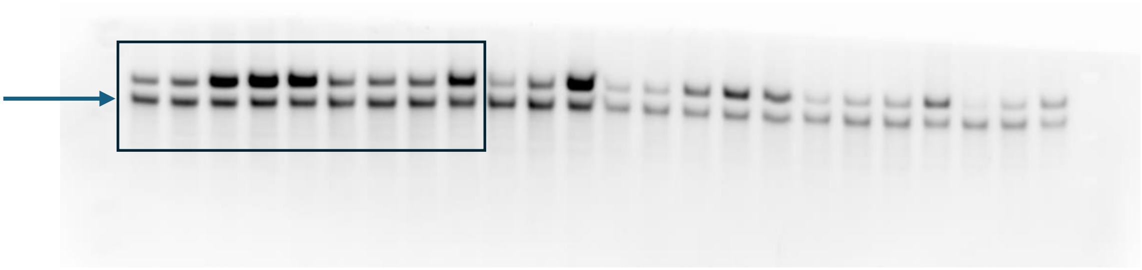

Figure 5

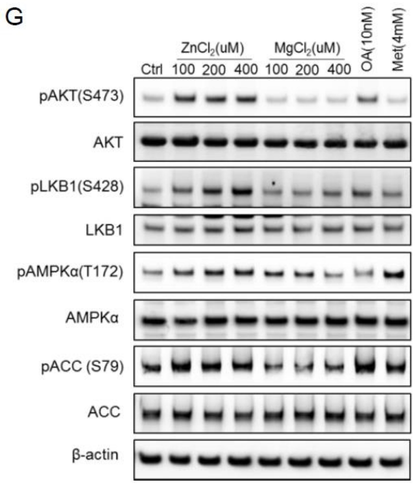
G
ZnCl2(uM)
MgCl2(uM)
Ctrl 100 200 400 100 200 400
OA(10nM)
Met(4mM)
pAKT(S473)
AKT
pLKB1(S428)
LKB1
pAMPKα(T172)
AMPKα
pACC (S79)
ACC
β-actin

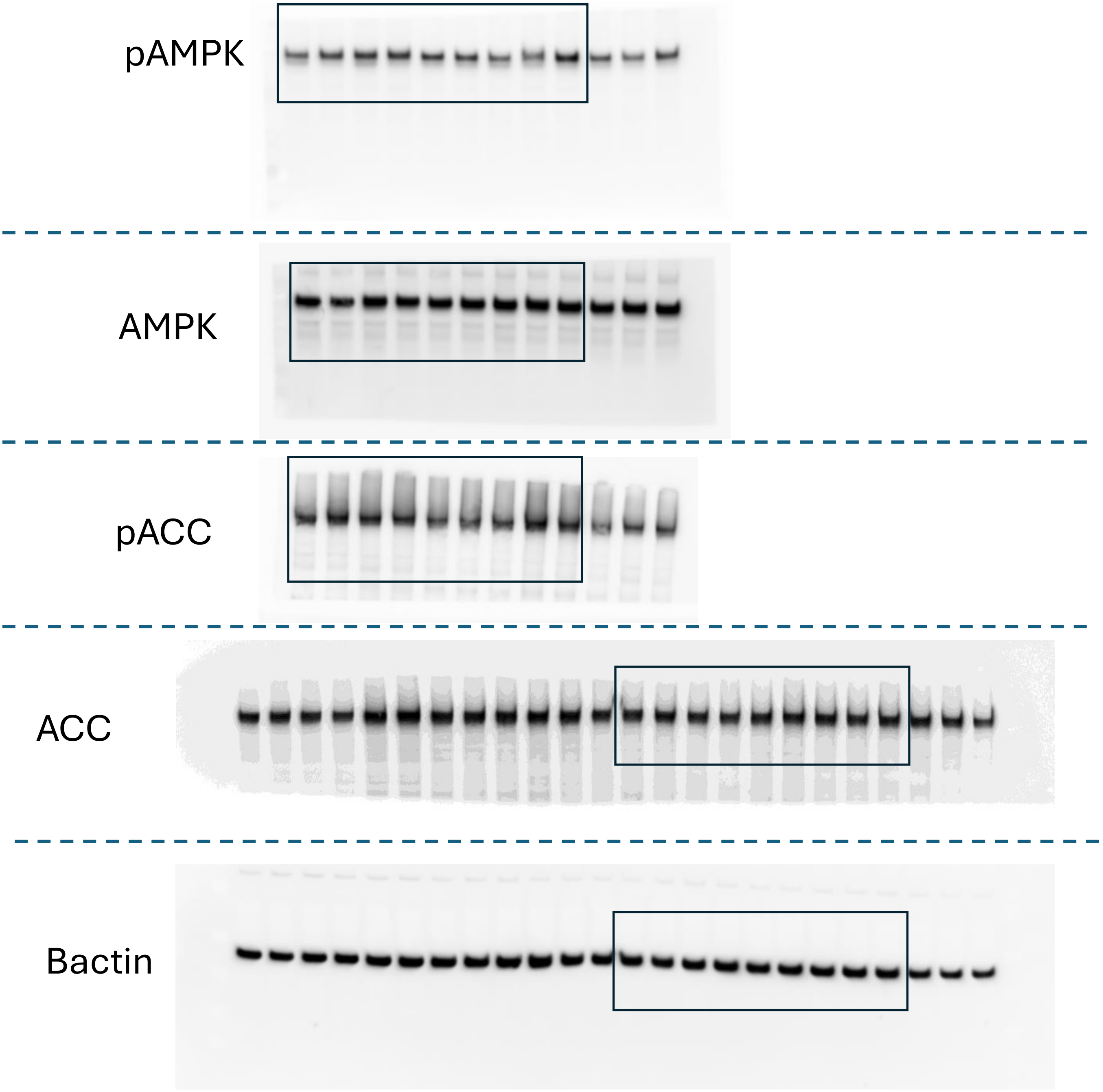
pAMPK
AMPK
pACC
ACC
Bactin
